# *CsMIKC1* regulates inflorescence development and grain production in *Cannabis sativa* plants

**DOI:** 10.1093/hr/uhae161

**Published:** 2024-06-12

**Authors:** Gencheng Xu, Yongbei Liu, Shuhao Yu, Dejing Kong, Kailei Tang, Zhigang Dai, Jian Sun, Chaohua Cheng, Canhui Deng, Zemao Yang, Qing Tang, Chao Li, Jianguang Su, Xiaoyu Zhang

**Affiliations:** Institute of Bast Fiber Crops, Chinese Academy of Agricultural Sciences, Changsa, Hunan 410205, China; State Key Laboratory of Crop Genetics & Germplasm Enhancement and Utilization, Jiangsu Engineering Research Center for Plant Genome Editing, Nanjing Agricultural University, Nanjing 210095, China; School of Pharmacy, Hunan Vocational College of Science and Technology, Changsa, Hunan 410004, China; Department of Horticulture and Landscape Architecture, Oklahoma State University, Stillwater, OK 74078, USA; College of Food Science and Biology, Hebei University of Science and Technology, Shijiazhuang, Hebei 050018, China; The College of Agriculture, Yunan University, Kunming, Yunnan 650504, China; Institute of Bast Fiber Crops, Chinese Academy of Agricultural Sciences, Changsa, Hunan 410205, China; School of Life Sciences, Nantong University, Nantong, Jiangsu 226019, China; Huazhi Biotech Co., Ltd, Changsha, Hunan 410128, China; Institute of Bast Fiber Crops, Chinese Academy of Agricultural Sciences, Changsa, Hunan 410205, China; Institute of Bast Fiber Crops, Chinese Academy of Agricultural Sciences, Changsa, Hunan 410205, China; Institute of Bast Fiber Crops, Chinese Academy of Agricultural Sciences, Changsa, Hunan 410205, China; Institute of Bast Fiber Crops, Chinese Academy of Agricultural Sciences, Changsa, Hunan 410205, China; State Key Laboratory of Crop Genetics & Germplasm Enhancement and Utilization, Jiangsu Engineering Research Center for Plant Genome Editing, Nanjing Agricultural University, Nanjing 210095, China; Institute of Bast Fiber Crops, Chinese Academy of Agricultural Sciences, Changsa, Hunan 410205, China; Institute of Bast Fiber Crops, Chinese Academy of Agricultural Sciences, Changsa, Hunan 410205, China

## Abstract

Female inflorescence is the primary output of medical *Cannabis*. It contains hundreds of cannabinoids that accumulate in the glandular trichomes. However, little is known about the genetic mechanisms governing *Cannabis* inflorescence development. In this study, we reported the map-based cloning of a gene determining the number of inflorescences per branch. We named this gene *CsMIKC1* since it encodes a transcription factor that belongs to the MIKC-type MADS subfamily. Constitutive overexpression of *CsMIKC1* increases inflorescence number per branch, thereby promoting flower production as well as grain yield in transgenic *Cannabis* plants. We further identified a plant-specific transcription factor, CsBPC2, promoting the expression of *CsMIKC1*. *CsBPC2* mutants and *CsMIKC1* mutants were successfully created using the CRISPR-Cas9 system; they exhibited similar inflorescence degeneration and grain reduction. We also validated the interaction of CsMIKC1 with CsVIP3, which suppressed expression of four inflorescence development-related genes in *Cannabis*. Our findings establish important roles for *CsMIKC1* in *Cannabis*, which could represent a previously unrecognized mechanism of inflorescence development regulated by ethylene.

## Introduction

*Cannabis sativa* L. is a diploid species with a chromosome count of 2*n* = 20, comprising nine pairs of chromosomes and a pair of sex-determining chromosomes (XY/XX) [1]. Since early Neolithic times in East Asia, it has been utilized in diverse domains, including cosmetics, textiles, food, and medicinal purposes [2]. *Cannabis* female inflorescences accumulate hundreds of specialized metabolites within their glandular trichomes [3]. While *Cannabis* is often steeped in controversy due to the synthesis of Δ9-tetrahydrocannabinol (THC), which produces psychoactive effects in humans, it also contains phytocannabinoids with therapeutic potential. These compounds have shown promise in treating complex neurological disorders and cancer [4, 5]. These phytocannabinoids include, for example, cannabidiol (CBD), cannabigerol (CBG), cannabichromene (CBC), and cannabinol (CBN) [6, 7]. *Cannabis* seeds are recognized as a functional food due to beneficial compositions of essential fatty acids, proteins, and antioxidants [8]. The global medical *Cannabis* market was valued at $3.5 billion at retail prices in 2019, and significant future growth is expected, with a $20.2 billion market value forecast from 2020 to 2025 [9]. As of 2020, *Cannabis*-based products for medicinal use have been legalized in over 50 countries, including Australia, Canada, Germany, Israel, China (Yunnan and Heilongjiang provinces), and most US states.

During the process of domestication of wild *Cannabis* into cultivated *Cannabis*, remarkable morphological transitions have been observed, such as compact plant architecture accompanied by increased flower production resulting from increased inflorescence number per branch [10]. The inflorescence of *Cannabis* consists of a highly branched compound raceme, comprising several higher-order condensed branchlets. The condensed branchlet develops at the apex of the main stem, as well as on second- and third-order branches. [10]. The inflorescence number per branch identified in this article is the number of phytomers developed at second-order branches, equivalent to the number of nodes at second-order branches. Since increasing yield has been the primary goal of *Cannabis* improvement, understanding the genetic mechanisms underlying female flower development is crucial. However, the key genes that regulate inflorescence number had not been characterized in *Cannabis*, which significantly hindered progress in the genetic improvement of flower production. In this study, we mapped a quantitative trait locus (QTL) determining the number of inflorescences per branch in *Cannabis* and subsequently cloned the gene *CsMIKC1* for the QTL. *CsMIKC1* increased both inflorescence number per branch and grain production, a process regulated transcriptionally by CsBPC2*.* We also found that CsVIP3 interacted with CsMIKC1 and repressed expression of four genes related to flower development. Furthermore, we validate a hypothesis that ethylene functions as a key signal in *Cannabis* inflorescence development. Our findings not only provide insights into the molecular mechanism for increasing inflorescence number in *Cannabis* but also provide a basis for proposing prospective strategies for the enhancement of yield production.

## Results

### A major quantitative trait locus associated with inflorescence number per branch

**Figure 1:**
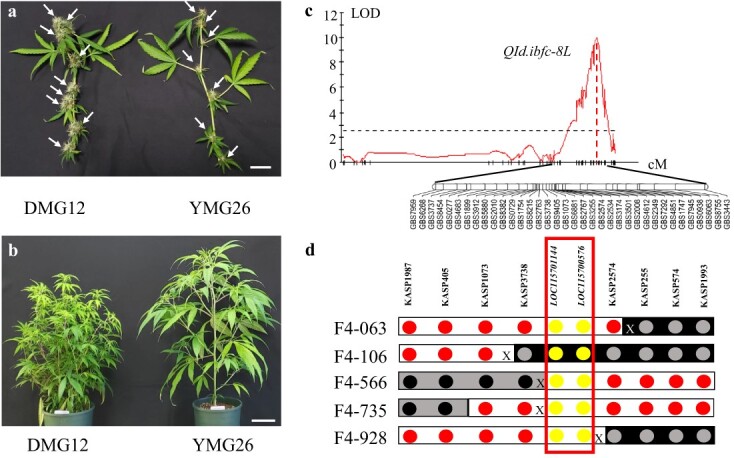
Mapping and positional cloning of *QId.ibfc-8 L*. **a** Typical branches of the two parental lines, DMG12 (left) and YMG26 (right). White arrows indicate inflorescences in each cultivar. The scale bar represents 1 cm. **b** Typical DMG12 and YMG26 plants grown in a greenhouse. The scale bar represents 10 cm. **c** Mapping of *QId.ibfc-8 L*, associated with inflorescence development. The GBS markers of a linkage group on chromosome arm 8 L are integrated with the data on inflorescent number from the *F*_2_ population. The physical locations of the GBS markers are provided in Supplementary Data S1. The horizontal dashed line represents the threshold log of the odds (LOD) value of 3.0. **d** Physical map of the crossovers detected in five critical recombinant plants. The primers used for KASP markers are listed in Supplementary Data Table S1. The genotype of each marker for the YMG26 allele, DMG12 allele, or heterozygotes is indicated above the circle representing the marker: red circles for the genotype of YMG26, gray circles for the genotype of DMG12, black circles for the genotype of heterozygotes, and yellow circles for the two candidate genes, *LOC115701144* and *LOC115700576.* X indicates a crossover between markers. *CsMIKC1* was delimited between two flanking markers, KASP3738 and KASP2574, in a 138 836-bp genomic region.

We performed a single cross between the two *Cannabis* accessions, DMG12 and YMG26, with distinct inflorescence morphologies (Fig. 1a and b). Subsequently, a population of 181 *F*_2_ plants was genotyped with the genotyping-by-sequencing approach. Phenotyping was carried out in the greenhouse. A major QTL associated with inflorescence development was mapped to the long arm of chromosome 8 (hereafter referred as *QId.ibfc-8 L*). The LOD (log of the odds) value of this QTL was 11.8, accounting for 35.7% of the total phenotypic variation (Fig. 1c). According to the NCBI *Cannabis sativa*.cs10 reference genome, *QId.ibfc-8 L* was mapped within a 3.1-Mb genome region between markers GBS7959 and GBS3443. To fine-map *QId.ibfc-8 L*, we developed eight internal markers using Kompetitive Allele-Specific PCR (KASP) (Supplementary Data Table S1 and S2) to screen 431 *F*_2:4_ individuals developed from the cross DMG12 × YMG26, and identified five new crossovers between KASP1987 and KASP1993 (Fig. 1d). Compared with recombinants carrying the YMG26 allele, those recombinants carrying the DMG12 allele at *QId.ibfc-8 L* had a significantly increased inflorescence number per branch (Supplementary Data Fig. S1). The candidate gene was narrowed down to a 138 836-bp genomic region flanked by two markers, KASP3738 and KASP2574. Based on the NCBI *Cannabis sativa*.cs10 reference genome, there are two candidate genes in this genomic region: *LOC115701144* and *LOC115700576*. To clone the gene responsible for *QId.ibfc-8 L*, alleles derived from DMG12 and YMG26 were sequenced for these candidate genes. The candidate gene *LOC115701144* encodes a truncated transcription factor, CAULIFLOWER A, and no difference was observed in the coding region between the DMG12 and YMG26 alleles (Supplementary Data Fig. S1). The transcript levels of *LOC115701144* did not show a significant difference between the DMG12 and YMG26 alleles (Supplementary Data Fig. S2). So *LOC115701144* was excluded as a candidate gene for *QId.ibfc-8 L*, and the other one, *LOC115700576*, was the sole candidate gene for *Qid.ibfc-8 L*.

**Figure 2:**
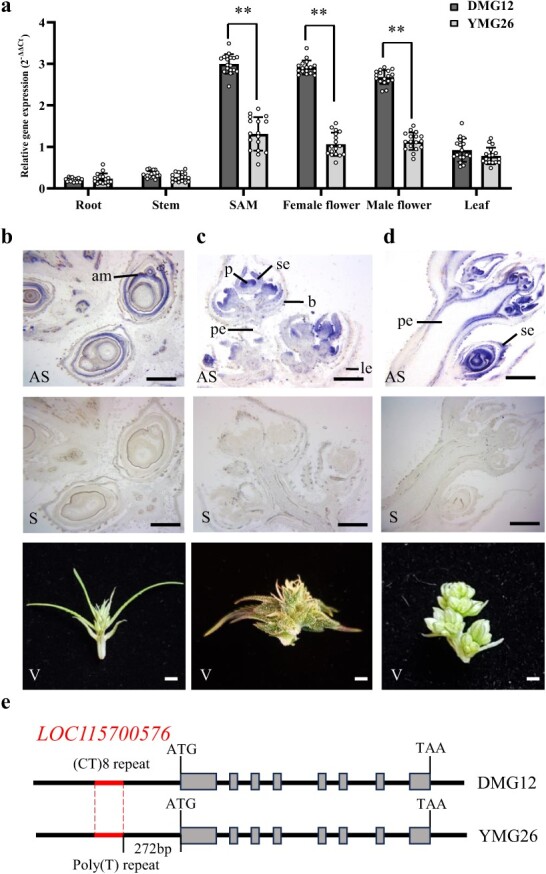
Identification of *CsMIKC1*. **a** Transcript levels of *CsMIKC1* in different tissues in DMG12 and YMG26. The RNA samples were collected 1 week after anthesis from different plant tissues, including root, stem, SAM, female flower, male flower, and leaf. Transcript levels of *CsMIKC1* were determined by qRT–PCR. The transcript value was calculated with the 2^–ΔΔCT^ method. We used a two-tailed unpaired Student’s *t* test to evaluate the mean transcript level between two alleles. Primers can be found in Supplementary Data Table S1. The bars in (**a**) indicate the standard error. **P < 0.001. **b**–**d**  *In situ* hybridization to detect the expression patterns of *CsMIKC1* in SAM (**b**), female flower (**c**), and male flower (**d**) with antisense *CsMIKC1* probe (AS). Control hybridization used the sense probe (S). Plant tissues are shown (V). b, bract; p, pistil; am, apical meristems; pe, peduncle; se, sepal; le, leaf. The experiments were repeated more than three times with similar results. Scale bars represent 100 μm. **e** Allelic variation among the DMG12 and YMG26 *CsMIKC1* alleles.

According to the NCBI *Cannabis sativa*.cs10 reference genome, *LOC115700576* encodes a transcription factor with 269 amino acids, which belongs to the MIKC-type MADS-domain subfamily. We therefore named this gene *CsMIKC1*. Using the *CsMIKC1* protein sequence to query the protein databases in NCBI GenBank, all the hits were hypothetical proteins (Supplementary Data Table S4). To date, *CsMIKC1* or its homologous gene has not been reported to participate in the regulation of inflorescence development. The *CsMIKC1* transcript levels in different tissues were evaluated using quantitative RT–PCR. In DMG12, the *CsMIKC1* gene was highly expressed in shoot apical meristems (SAMs) and female and male flowers compared with the expression levels in leaf, stem, and root (Fig. 2a). *Csmikc1* in YMG26 showed a similar expression pattern to *CsMIKC1* in DMG12, but no significantly higher expression in the SAM or flowers compared with other organs. *In situ* hybridization during flower development further revealed that *CsMIKC1* mRNA distribution was more abundant in floral organs and apical meristems. The signal intensity was higher on the adaxial sides of the SAM, as well as the floral meristems and young bracts, but, by contrast, the signal was weak in the peduncle, older bracts, and leaves (Fig. 2b–d). The expression patterns of *CsMIKC1* suggest that this gene may have a general role in promoting cell proliferation and differentiation in floral organs and apical meristems, leading to a hypothesis that the phenotypic differences were probably determined by *CsMIKC1* at the transcript level.

### A key regulatory DNA element differentiated transcript levels of the two *CsMIKC1* alleles

**Figure 3:**
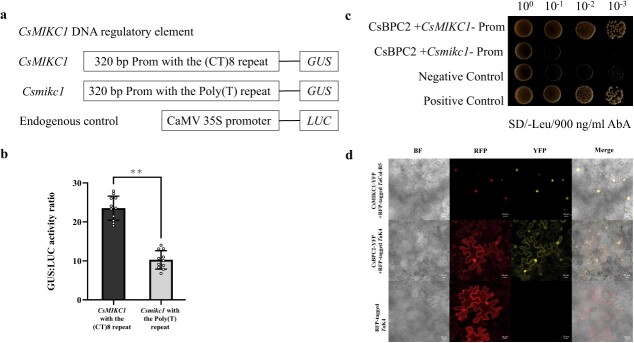
CsBPC2 binds to the *CsMIKC1* promoter region. **a** The constructs are designed for the regulatory elements in *CsMIKC1*. The 320-bp region of *CsMIKC1* promoter from DMG12 with the (CT)_8_ repeat fragment, and from YMG26 with the poly(T) fragment. For the endogenous control, the 35S promoter was used to drive the LUC gene. **b** Comparison of GUS/LUC activity ratio. The GUS and LUC activities were tested in transient expression assays in *Cannabis* protoplasts. Data are presented from 11 independent reactions for each construct (*n* = 11). **P < 0.001. **c** Y1H assay for binding of *CsBPC2* directly to the promoter region of DMG12 and YMG26 *CsMIKC1* alleles. The 320-bp promoter regions of *CsMIKC1* from DMG12 and YMG26 were inserted into the pAbAi vector as a bait, and *CsBPC2* was inserted into the pGADT7 vector as prey. Positive control (pGADT7-p53 + pAbAi-p53) and negative control (pGADT7 empty vector+ pAbAi-*CsMIKC1*-Prom) were set on the plate together. Co-transformed cells were grown on SD/−Leu plates with 900 ng/ml aureobasidin A (AbA). Colony solutions diluted to four levels were incubated on the plate. **d** Subcellular locations of CsBPC2 and CsMIKC1 in tobacco leaves. Enriched yellow fluorescent signals associated with CsBPC2 were detected predominantly in the nucleus and on the membranes. CsMIKC1 was detected predominantly in the nucleus. Leaves infiltrated with *A. tumefaciens* were imaged with an LSM780 laser scanning confocal microscope in the bright field or with red fluorescent protein (RFP) or yellow fluorescent protein (YFP) filter. The RFP fluorescence marker under a constitutive promoter was included in each negative control infiltration mixture as an indicator of successful infiltration. To identify the nuclear localization signal, RFP-tagged *Ta*Col-B5 was used as a marker for the nucleus and RFP-tagged *Ta*K4 was used as a marker for the nucleus and plasma membrane, whose locations were detected in our previous work [40]. Scale bars represent 20 μm.

We observed that the DMG12 *CsMIKC1* allele contained a (CT)_8_ repeat sequence in the promoter region compared with a poly(T) sequence of the YMG26 *Csmikc1* allele (Fig. 2e). To test whether the (CT)_8_ repeat is involved in regulation of transcription, we developed two constructs to investigate the expression of the reporter gene in the GUS expression system transiently. The *CsMIKC1*-Prom construct includes 320 bp before the start codon from the DMG12 allele, and the *Csmikc1*-Prom construct includes the same region of the YMG26 allele (Fig. 3a). The two constructs had the identified promoter region (320 bp), the only difference being the (CT)_8_ repeat in *CsMIKC1*-Prom and poly(T) sequence in *Csmikc1*-Prom instead. As an internal control, the luciferase (*LUC*) gene provided an estimate of the expression efficiency. *CsMIKC1*-Prom and *Csmikc1*-Prom were co-transformed with the construct of *LUC* into *Cannabis* protoplasts. In the transformed protoplasts, *CsMIKC1*-Prom showed a higher expression level of GUS than *Csmikc1*-Prom, which lacks the (CT)_8_ repeat sequence (Fig. 3b). This result was the evidence that the (CT)_8_ repeat sequence played an essential role in regulating the transcription of *CsMIKC1*. To identify transcription factors that interacted with the *CsMIKC1* promoter region, the promoter fragment (320 bp) was used to screen a yeast one-hybrid (Y1H) cDNA library prepared from the cultivar DMG12 (female flowers). Twenty-eight positive clones were obtained. These clones were sequenced by BLAST (Supplementary Data Table S2). A cDNA ORF encoding a putative BASIC PENTACYSTEINE2 (BPC2) protein (183 amino acids) was identified with the C-terminal conserved region of the BPC members. The interaction between CsBPC2 and the 320 bp *CsMIKC1* fragment was confirmed using a full-length *CsBPC2* clone (855 bp, *LOC115722185*) in an independent Y1H assay with the 320 bp *CsMIKC1* promoter fragment (Fig. 3c). When the 320 bp *Csmikc1* promoter fragment was used as a bait, *CsBPC2* did not show interaction. These results indicated that *CsBPC2* binds to the promoter region of *CsMIKC1* but not *Csmikc1* due to the presence of the (CT)_8_ repeat. To observe their subcellular locations, the *CsBPC2* and *CsMIKC1* proteins were expressed in tobacco leaf cells using pEG101-YFP (YFP, yellow fluorescent protein) vector (Fig. 3d). Significant yellow fluorescent signals of *CsMIKC1* and *CsBPC2* were predominantly observed on the membrane and also in the nucleus.

### Genetic effects of *CsMIKC1* on inflorescence architecture in transgenic *Cannabis*

**Figure 4:**
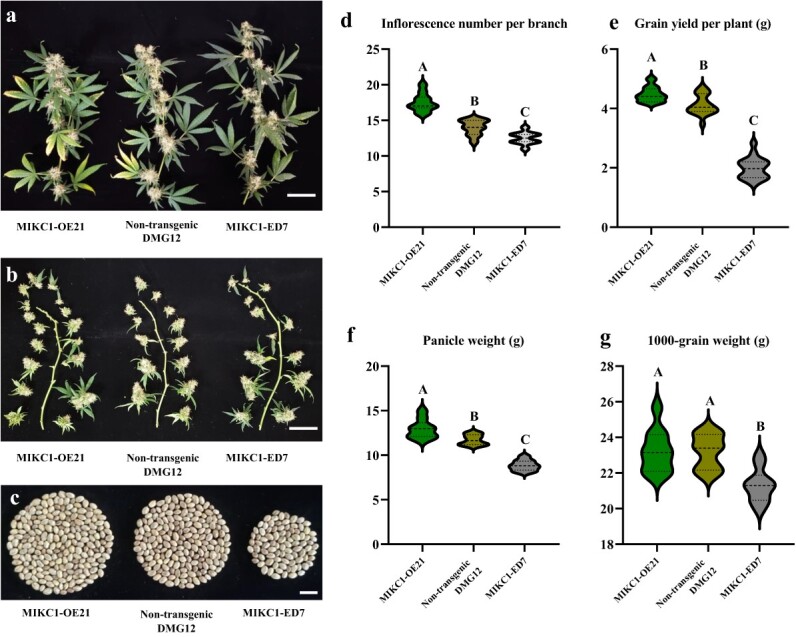
Performance of *CsMIKC1*-overexpressing transgenic plants and *CsMIKC1* mutants in the greenhouse. **a**, **b** Dissected branches of MIKC1-OE21 with *CsMIKC1* overexpressed, and MIKC1-ED7 with *CsMIKC1* edited. **c** Grains per plant harvested from (left to right) MIKC1-OE21, non-transgenic DMG12, and MIKC1-ED7 plants. Scale bars represent 1 cm. **d**–**g** Average effects of *CsMIKC1* on inflorescence number per branch (**d**), grain yield per plant (**e**), panicle weight (average weight of all inflorescences collected from one branch) (**f**), and 1000-grain weight (**g**) over the transgenic families. Each transgenic event was designed to have 19 independent plants for testing. There were 19 non-transgenic plants set as control. The mean value was statistically analyzed, and ANOVA with Tukey’s HSD test was used to determine the significance level between non-transgenic and transgenic plants. Uppercase letters indicate significant differences (*P* < 0.001). Bars indicate the standard error.

We cloned the cDNA of the *CsMIKC1* allele into the pNC-Cam3304-MCS35S vector containing the constitutively expressed CaMV 35S promoter and transformed this construct into DMG12 as the host plant but not into YMG26, because it is currently not transformable. We obtained three female *T*_0_ plants, designated MIKC1-OE21, MIKC1-OE33, and MIKC1-OE56. By crossing with the male DMG12 plants, each of these *T*_0_ plants generated a *T*_1_ family. Positive *T*_1_ plants were screened with a pair of primers, OE-TEST-F1 and OE-TEST-R3. The *T*_1_ family showed a 1:1 segregation ratio between non-transgenic and transgenic plants. This result indicated the presence of *CsMIKC1* expression cassette CaMV 35S-*CsMIKC1-NOS* integrated at a single locus. qRT–PCR was used to confirm the expression of *CsMIKC1* in the transgenic *Cannabis* individuals (Supplementary Data Fig. S3). We also designed constructs using CRISPR-Cas9, enabling us to edit the sequence in DMG12. Two independent transgenic events (*T*_0_ plants) were obtained and designated MIKC1-ED7 and MIKC1-ED22. The MIKC1-ED7 editing event contained a 5-bp deletion. The MIKC1-ED22 editing event contained a 1-bp insertion. Each of the two editing events resulted in a frameshift (Supplementary Data Fig. S4). The MIKC1-ED7 sequence was found to have a predicted loss of 242 amino acids starting at position 27, including a loss of 51 amino acids in the MADS-MEF2-like domain, and the MIKC1-ED22 sequence had a predicted 240 amino acid loss starting at position 29, with a 49 amino acid loss in the MADS-MEF2-like domain. Transgenic plants in the greenhouse were observed with clear phenotypic segregation from the non-transgenic individuals in *T*_1_ families. Compared with the non-transgenic ones, transgenic ones overexpressing *CsMIKC1* produced more inflorescences and grains, while the null *CsMIKC1* mutants exhibited less compact architecture and significant reductions in inflorescence number and grain production (Fig. 4a–c). In the null *CsMIKC1* mutants, the inflorescence number per branch decreased by 1.6 on average compared with the non-transgenic plants. The average weight of all the inflorescences collected from one branch (panicle weight) reduced by 24.5% in *CsMIKC1* mutants (Fig. 4d–f). Plants overexpressing *CsMIKC1* set 3.1 more inflorescences per branch and provided an 8.5% increase in grain yield as well as a 10.4% increase in panicle weight, compared with non-transgenic ones. The 1000-grain weight was similar between non-transgenic and transgenic plants overexpressing *CsMIKC1*, while the *CsMIKC1* mutants exhibited a significant reduction of 8.2% compared with the non-transgenic plants (Fig. 4g).

### *CsBPC2* mutants exhibit inflorescence degeneration and ethylene insensitivity

**Figure 5:**
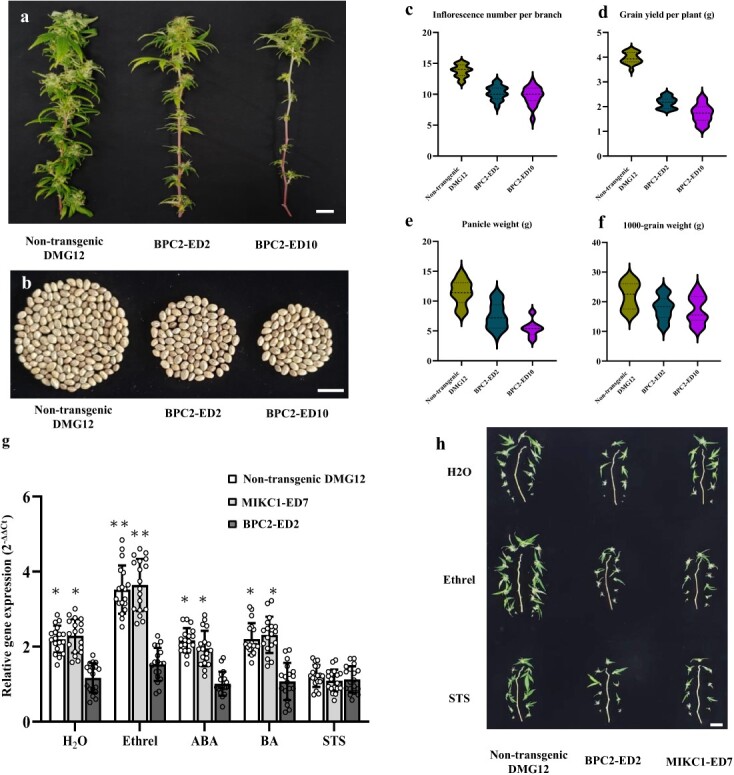
The *CsBPC2* mutants show decreased sensitivity to ethylene. **a** Image of inflorescence degeneration on branches of BPC2-ED2 and BPC2-ED10. Branches were sampled from non-transgenic DMG12, BPC2-ED2, and BPC2-ED10 (left to right) grown in a greenhouse. The scale bar represents 1 cm. **b** Grains per plant harvested from non-transgenic DMG12, BPC2-ED2, and BPC2-ED10 (left to right) plants. The scale bar represents 1 cm. **c**–**f**. Average effects of *CsBPC2* on inflorescence number per branch (**c**), grain yield per plant (**d**), panicle weight (**e**), and 1000-grain weight (**f**) over the transgenic families. The mean value was statistically analyzed, and ANOVA with Tukey’s HSD test was used to determine the significance level between non-transgenic (18 plants) and transgenic plants (18 plants for each of the mutation lines). Uppercase letters indicate significant differences (*P* < 0.05). Bars indicate the standard error. **g** Relative expression levels of *CsMIKC1* in non-transgenic DMG12, MIKC1-ED7, and BPC2-ED2 plants treated with H_2_O, Ethrel, ABA, BA, and STS. Gene expressions were quantified by qRT–PCR with 18 biological samples and the transcription level was calculated with the 2^–ΔΔCT^ method, where CT is the threshold cycle. Two-tailed unpaired Student’s *t*-test was used to evaluate the mean transcript level between the two alleles (Supplementary Data Table S1). The comparison was performed between BPC2-ED2 and non-transgenic/MIKC1-ED7 in each treatment. ^*^P < 0.05; ^**^P < 0.01. **h** Ethrel and STS applications on CsBPC2 mutants, CsMIKC1 mutants, and non-transgenic plants to show the effect of ethylene and STS on the inflorescence number. The scale bar represents 1 cm.

To validate if *CsMIKC1* expression is regulated by *CsBPC2*, we cloned the *CsBPC2* alleles derived from DMG12 and YMG26. The sequences of the coding region and 1.5 kb before the start codon were the same in DMG12 and YMG26. Next, the transcript levels of *CsBPC2* did not show significant differences in different plant tissues in DMG12 and YMG26 (Supplementary Data Fig. S5). To further examine the function of *CsBPC2*, we developed *CsBPC2* knockout mutants in DMG12 using CRISPR-Cas9. We successfully created two *CsBPC2* loss-of-function mutants (BPC2-ED2 and BPC2-ED10) using CRISPR-Cas9 genome editing. The BPC2-ED2 editing event caused a predicted loss of 163 amino acids starting at position 121, and the BPC2-ED10 editing event caused a loss of 172 amino acids starting at position 112 (Supplementary Data Fig. S6). Compared with the non-transgenic plants, the *CsBPC2* mutants exhibited significant basipetal inflorescence degeneration and reduced grain production (Fig. 5a–c). Lower flower survival at the heading stage decreased the final flower and grain production in edited plants. The null *CsBPC2* mutants exhibited significant reductions in inflorescence number per branch, grain yield per plant, panicle weight, and 1000-grain weight compared with the non-transgenic plants (Fig. 5d–g).

Results of previous investigations showed that BPCs are part of a complex network of transcription factors that are involved in the response to ethylene, abscisic acid (ABA), or cytokinin [11, 12]. To elucidate if *CsBPC2* and *CsMIKC1* were linked to signaling pathways of these phytohormones, we investigated the response of *CsBPC2* and *CsMIKC1* to treatments with Ethrel (a kind of synthetic ethylene), silver thiosulfate (STS, an ethylene inhibitor), ABA and benzyladenine (BA, a kind of synthetic cytokinin). Solutions of these phytohormones were sprayed on the inflorescence of *CsBPC2* mutants, *CsMIKC1* mutants, and non-transgenic plants. Treatment with demineralized water was used as control. The qRT–PCR analysis demonstrated that *CsBPC2* expression did not show significant differences after spraying these phytohormones (Supplementary Data Fig. S7). Surprisingly, after spraying Ethrel solution the transcript level of *CsMIKC1* increased in the *CsMIKC1* mutants and non-transgenic plants, while in the STS treatment the expression level of *CsMIKC1* was significantly decreased compared with the control (Fig. 5h). The application of ABA or BA did not affect the expression level of *CsMIKC1* significantly compared with the control. In the Ethrel, ABA, and BA treatments, the transcript levels of *CsMIKC1* in *CsBPC2* mutants were always lower than in *CsMIKC1* mutants and non-transgenic plants. The application of STS mitigated the effects of ethylene on *CsMIKC1* transcript in the *CsMIKC1* mutants and non-transgenic plants. Compared with the water treatment, spraying Ethrel increased panicle weight by 7.96%, and the flowering dates were 4.23 days earlier, while STS greatly reduced panicle weight and caused an average delay of 3.22 days in flower initiation (Supplementary Data Table S3). In the CsBPC2 mutants and CsMIKC1 mutants, spraying phytohormones did not increase or decrease the inflorescence number compared with the water treatment. The loss of function of CsBPC2 led to reduced *CsMIKC1* expression and decreased sensitivity to ethylene, and therefore supported the involvement of the *CsBPC2* and *CsMIKC1* genes in ethylene signaling. This study provides an example showing that spraying exogenous ethylene can be used to promote inflorescent growth and shorten the period of growing in *Cannabis* commercial production.

### CsMIKC1 interacts with CsVIP3 *in vitro* and *in vivo*

**Figure 6:**
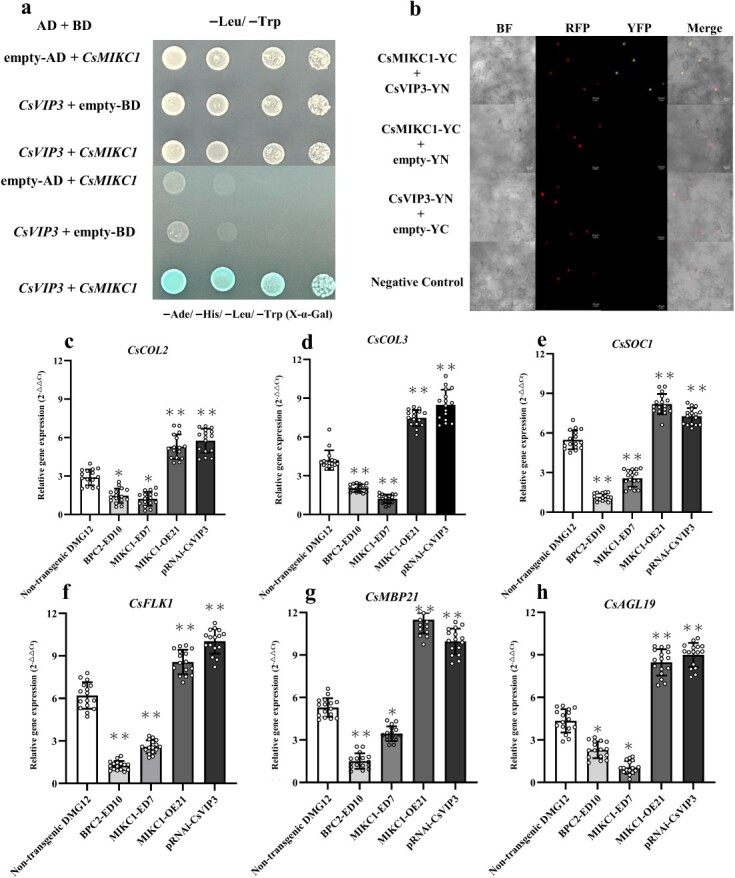
Interactions of CsMIKC1 proteins with CsVIP3 and their effects on inflorescence development-related gene expression. **a** Y2H interactions of CsMIKC1 proteins with CsVIP3. Full-length CsMIKC1 proteins were tested for interactions with TaK4 in comparative Y2H interaction studies. Co-transformed cells were incubated on the same plates without two amino acids (−Leu/−Trp) as well as plates without four amino acids (−Leu/−Trp/−His/−Ade). The colony solutions were diluted 4-fold and incubated on the same plate for the protein pair. The CsMIKC1-bait and empty prey combination did not grow on the plate lacking Leu/Trp/His/Ade (negative control). **b** Interaction of CsMIKC1 fused with YC and CsVIP3 fused with YN. YN is the N-terminal fragment of YFP; YC is the C-terminal end fragment of YFP. Negative controls included the co-expression of CsMIKC1-YC with an empty-YN, CsVIP3-YN with an empty-YC, and an empty-YN with an empty-YC. In the YFP filter, no interaction was observed in the negative control treatments. The RFP fluorescence marker under a constitutive promoter was included in each negative control infiltration mixture as an indicator of successful infiltration. To identify the nuclear localization signal, RFP-tagged *Ta*Col-B5 was used as a marker for the nucleus, as mentioned in our previous publication [43]. Leaves infiltrated with *A. tumefaciens* were imaged by the LSM780 laser scanning confocal microscope using the bright field, red fluorescent protein (RFP) filter, or yellow fluorescent protein (YFP) filter (scale bars represent 20 μm). **c**–**h** Comparison of gene expression in non-transgenic DMG12, BPC2-ED10, MIKC1-ED7, MIKC1-OE21, and pRNAi-CsVIP3 plants. After anthesis, samples of female flowers from 16 different plants (*n* = 16) of each transgenic line were used to study the expressions of *CsCOL2* (**c**), *CsCOL3* (**d**), *CsSOC1* (**e**), *CsFLK1* (**f**), *CsAGL19* (**g**), and *CsMBP21* (**h**) under short-day conditions. Gene expressions were quantified by qRT–PCR with 18 biological samples. Two-tailed unpaired Student’s *t*-test was used to evaluate the mean transcript level between two alleles (Supplementary Data Table S1). ^*^P < 0.05, ^**^P < 0.01 vs non-transgenic DMG12 plants.

Yeast two-hybrid (Y2H) screens were performed to identify candidate genes that interact with *CsMIKC1*. Using a full-length *CsMIKC1* cDNA as bait, 12 interacting clones were identified, rescued from yeast, and transformed into *Escherichia coli*. All of these clones were from the same gene, *LOC115707890*, located on chromosome 1, which is an ortholog of *Arabidopsis VERNALIZATION INDEPENDENCE 3* (*VIP3*), encoding a WD40 repeat protein (GenBank accession number Q9SZQ5). The *Cannabis* ortholog of *AtVIP3* is referred to as *CsVIP3*. To confirm the specificity of the observed interaction, the whole sequence of *CsVIP3* cDNA was transformed back into a yeast strain containing the *CsMIKC1* bait. Strains containing the *CsMIKC1* bait tested positive for both X-α-Gal activity and HIS prototrophy. Strains containing the empty bait vector were negative, as they were not able to grow on plates lacking histidine and the yeast colonies were completely white in the X-α-Gal assay (Fig. 6a). We further explored the interactions between *CsVIP3* and *CsMIKC1* by the transient expression system in leaves of *Nicotiana benthamiana* (Fig. 6b). *CsMIKC1* was independently fused to the C-terminal amino acid portion of YFP. *CsVIP3* was fused to the N-terminal of YFP. To test their *in vivo* interaction, the pEarleyGate202-YC vector containing *CsMIKC1* was co-transformed into the leaves of *N. benthamiana* with the pEarleyGate201-YN vector containing *CsVIP3*. Enriched yellow fluorescent signals were detected mainly in the nucleus. These results confirmed the specificity of the observed interactions between *CsVIP3* and *CsMIKC1*.

### Downstream targets regulated by CsMIKC1

Previous studies showed that MADS-box transcription factors can directly target genes by binding to the CArG box motif, and a set of genes controlling flower development were prominent members of the MADS-box network [13]. To establish a signaling network of inflorescent development, we detected which genes were affected in the MIKC1-OE21, BPC2-ED10, and MIKC1-ED7 *T*_2_ families (non-transgenic plants as control). We cloned 17 homologous genes which were commonly demonstrated to promote inflorescence development in other dicot plant species [13–15] (Supplementary Data Fig. S8), and tested their transcript levels. Compared with the expression of non-transgenic individuals, the transcript levels of *CsCOL2*, *CsCOL3*, *CsSOC1*, *CsFLK1*, *CsMBP21*, and *CsAGL19* all increased in transgenic DMG12 plants overexpressing *CsMIKC1*, while the expression of these genes decreased significantly in the BPC2-ED10 and MIKC1-ED7 mutats (Fig. 6c–g). To elucidate the role of *CsVIP3* in this pathway, a recombinant *Agrobacterium* strain carrying a RNAi construct was infiltrated in stipule segments (a component of the female flower) of DMG12 plants, which downregulated *CsVIP3* expression. From the qPCR data, pRNAi-CsVIP3 treatment saw a 63% reduction in *CsVIP3* transcript level compared with the plants infiltrated with disarmed *Agrobacterium* (Supplementary Data Fig. S9). Furthermore, qPCR results demonstrated that the expression levels of the six genes regulated by *CsMIKC1* were significantly upregulated when *CsVIP3* was silenced, indicating that *CsVIP3* may function as a negative regulator of inflorescence development in *Cannabis*. This result provided experimental evidence that expressions of the six inflorescence development-related genes could be associated with the functions of *CsBPC2*, *CsMIKC1*, and *CsVIP3*, leading to an overall manipulation of the inflorescence architecture in *Cannabis*.

## Discussion

We cloned the gene *CsMIKC1*, and elucidated the signaling networks governing the number of inflorescences per branch in *Cannabis*. The dominant *CsMIKC1* allele contained a (CT)_8_ insertion in the promoter region, which is a binding site for CsBPC2, a transcription factor involved in the ethylene signaling pathway. We successfully created null *CsBPC2* mutants and null *CsMIKC1* mutants, which exhibited similar inflorescence growth arrest and floral degeneration. The expression level of *CsMIKC1* in *CsBPC2* mutants was significantly lower compared with the non-transgenic plants, providing evidence that *CsBPC2* works as an upstream regulator of *CsMIKC1* expression. Furthermore, we identified a flowering repressor, CsVIP3, validated its interaction with CsMIKC1 in living cells, and identified tentative target genes whose transcription could be affected.

Numerous MADS-box transcription factors have been shown to regulate flower development and growth regulation in plant species such as in rice, maize, and *Arabidopsis* [16, 17]. *OsMADS34* mutants exhibited an altered inflorescence phenotype characterized by an increased branch number, reduced spikelet count, and changes in spikelet morphology [18]. Loss of function of maize *ZAG3*, a homologous gene of *OsMADS6*, led to spikelets producing an increased number of florets with additional sterile ovaries and lemma-like organs [19]. Overexpression of the SVP-group MADS-box genes produced floral reversion and flower deformities in *Arabidopsis* [20]. To date, *CsMIKC1* is the first MADS-box gene that has been identified to have significant effects on *Cannabis* inflorescence development. The cloning of *CsMIKC1* provides an alternative strategy to develop new cultivars with more inflorescences and higher grain production through transformation of *CsMIKC1* as a single gene in *Cannabis* with various genetic backgrounds. In this study, transformation of *CsMIKC1* in DMG12, which has a specific genetic background adapted to the local environments, increased the inflorescence number per branch and led to a 10.4% increase in flower production as well as an 8.5% increase in grain yield. The panicle morphology of the transgenic plants indeed showed dramatic changes, but due to the limitation of available grains the *CsMIKC1* effects were characterized in a 2-year experiment under controlled environmental conditions with sufficient irrigation and fertilizers. An overall evaluation of *CsMIKC1* effects on inflorescence number with various genetic backgrounds and different environments is needed in future studies.

Moreover, the results indicate that both *CsBPC2* and *CsMIKC1* are involved in the ethylene signaling pathway to affect inflorescence development in *Cannabis*. *BPC* genes can be directly regulated by ethylene signaling in *Arabidopsis*, which was observed in previous studies [12, 21]. Similarly, the ethylene response is diminished in *CsBPC2* frameshift mutants. Foliar spraying of exogenous ethylene promoted *CsMIKC1* expression in *CsMIKC1* mutants and non-transgenic plants. However, the expression level of *CsMIKC1* was not significantly increased or decreased in the *CsBPC2* frameshift mutants, which supported the hypothesis that loss of function of CsBPC2 led to reduced ethylene sensitivity in these mutants. The effect of applications of ethylene and its inhibitor (STS) supported the biological relevance of ethylene in *Cannabis* flowering and inflorescence development. Prior research has elucidated the functional role of ethylene in floral promotion in fruits, such as pineapple, mango, and lychee [22]. The data suggest that spraying exogenous ethylene promoted inflorescence growth and shortened the period of growth in *Cannabis*. This provides an example of how exogenous ethylene can increase flower production in commercial *Cannabis* cultivation.

It is worth noting that CsVIP3 may function as a negative regulator of inflorescence development in *Cannabis*. *VIP* genes define a mechanism involved in multiple developmental processes, including flowering and floral development [23]. In *Arabidopsis*, the *VIP* loci encoded a group of flowering repressors previously unreported [24]. While the relationships among these genes remain largely unclear, the evidence suggested that these genes encode defined components of a protein complex. It has been demonstrated that the *VIP3* gene encodes an SKI (cytoplasmic Superkiller) complex component that affects the stability of mRNA and leads to late flowering and aberrant flower development in *Arabidopsis* [25, 26]. Our results also confirmed the specificity of the observed interactions between CsVIP3 and CsMIKC1 *in vivo* and *in vitro*, and hence CsVIP3 and CsMIKC1 may function as components of a protein complex to regulate inflorescence development in *Cannabis*. If this were the case, then loss of CsVIP3 function would not be expected to suppress the inflorescence development-related genes. To test this, we evaluated the expression of 17 homologous genes related to floral development in other plant species. Meanwhile, we investigated their expression in *CsVIP3*-silenced plants, *CsMIKC1*-overexpressing transgenic plants, *CsMIKC1* mutants, and *CsBPC2* mutants.

**Figure 7:**
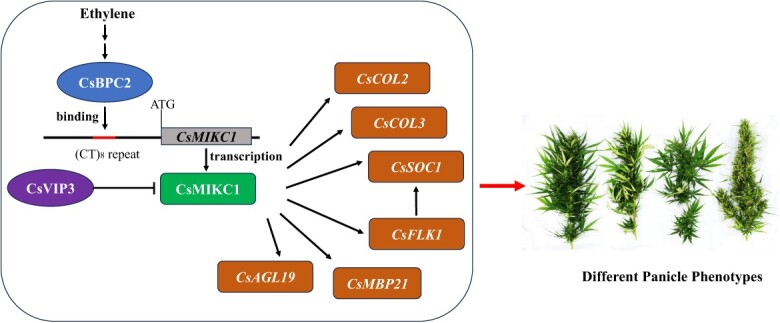
Model for inflorescence development regulation by the ethylene pathway in *Cannabis*. ⊣ indicates repression. Arrows indicate promotion. Non-italic names indicate proteins. Italic names indicate genes.

Notably, the expression of six genes (*CsCOL2*, *CsCOL3*, *CsSOC1*, *CsFLK1*, *CsMBP21*, and *CsAGL19*) was promoted in the lines overexpressing *CsMIKC1* or silencing *CsVIP3*, while their expression was suppressed significantly in the *CsMIKC1* mutants and *CsBPC2* mutants. These genes may function in the same network to prevent or induce inflorescence development, regulated by *CsBPC2*, *CsMIKC1*, and *CsVIP3* (Fig. 7). The *CONSTANS-like* (*COL*) gene family was predicted to play a core role in regulating flowering time in *Cannabis* [27]. As a member of the MADS-box gene family, *SUPPRESSOR OF OVEREXPRESSION OF CO 1* (*SOC1*) homologs in *Arabidopsis* are known to play significant roles in regulating floral development and controlling flowering time [28]. In *Rosa odorata*, *SOC1* represses the expression of *GID1B*, a gibberellin (GA) receptor involved in regulating flower development, while activating expression of *FRUITFULL* (*FUL*) and *poly(A) binding* (*PAB*), and enhancing flower initiation and seed production [29]. It is worth mentioning that *FLOWERING LOCUS C* (*FLC*), a suppressor of flower initiation, can directly repress *SOC1* in the inflorescence meristem, while *FLOWERING LOCUS K* (*FLK*) primarily acts as an inhibitor of *FLC* expression, and hence promotes *SOC1* expression [30], which explain the significant upregulation of both *CsSOC1* and *CsFLK1* in plants overexpressing *CsMIKC1* or with silenced *CsVIP3.* In *Arabidopsis*, the genes exhibiting reduced expression in the *flk* mutant, such as *FLOWERING LOCUS T* (*FT*), *PSEUDO-RESPONSE REGULATOR5* (*PRR5*), and *PRR7*, are recognized as positive regulators of floral initiation [31]. Silencing of *MADS-box protein 21* (*MBP21*) can activate the expression of ten *1-aminocyclopropane-1-carboxylate synthase* (*ACS*) genes and eight *1-aminocyclopropane-1-carboxylate oxidase* (*ACO*) genes, and affect the ethylene and auxin levels in tomato sepals [32]. Moreover, the chloroplast content and Rubisco activity in SlMBP21 mutants are detected dramatically higher than in the wild type the Chl contents, which can improve photosynthetic efficiency in sepals [33]. *AGAMOUS-LIKE 19* (*AGL19*), together with *AGL24* and *SOC1*, coordinately activates the expression of floral meristem identity genes, including *LEAFY* (*LFY*)*, APETALA1* (*AP1*), and *FT*, to promote flowering [34]. AGL19 participates in the regulation of the flowering process through the *HISTONE DEACETYLASE 9* (*HDA9*)-AGL19-FT model [35]. We hypothesize that the inflorescence development-related genes mentioned above could be potential components in the ethylene signaling pathway to regulate inflorescence development in *Cannabis* and finally contribute to diversity in yield production. Verification of their functions will be done to further understand the molecular mechanisms and regulatory network of inflorescence development in *Cannabis*.

In conclusion, our findings reveal the molecular mechanisms driving the development of female flowers in *Cannabis*. The cloning of *CsMIKC1* serves as a starting point for elucidating the functions of numerous orthologous genes involved in inflorescence development. This understanding can facilitate the modification of inflorescence architecture and maximize plant productivity in *Cannabis*. In addition, our findings suggest that ethylene plays a role in positively regulating *Cannabis* inflorescence production, and could be widely used in its commercial cultivation.

## Materials and methods

### Mapping and cloning of *QId.Ibfc-8 L*

DMG12 and YMG26 grains were provided by the national germplasm bank, Chinese Academy of Agriculture Sciences (CAAS). By inducing sex change and self-pollination, we have purified the two cultivars in eight generations separately. In addition, we have randomly genotyped 1000 individuals of each cultivar using a 20 K SNP chip that includes 20 626 SNPs (Huazhi Biotech, Changsha, China). Data showed that, in each cultivar, the heterozygosity ratio of 20 626 SNPs was <0.3%, which provided strong evidence that DMG12 and YMG26 are not highly heterozygous plants. The *F*_1_ hybrid was created by crossing DMG12 as the male parent with YMG26 as the female parent. DMG12 parental line hermaphrodites were generated by GA treatment. The concentration of GA solution was 50 μg/l. Female plants were sprayed three times before flowering and the whole treatment lasted 10 days [36] . GA solutions were applied to the *F*_1_ female plants on the first, fifth, and tenth days, after which the plants began producing male flowers at the newly formed nodes. Self-pollination of the resulting *F*_1_ plants generated 181 *F*_2_ plants, which were used for phenotyping and genotyping. The phenotypic traits of the plants were correlated with genotyping-by-sequencing (GBS) markers, leading to the mapping of *QId.ibfc-8 L* to the long arm of chromosome 8. This region had not been previously associated with inflorescence development. Based on the primers for KASP markers (Supplementary Data Table S1), the positional cloning approach was used to clone *QId.ibfc-8 L*. DMG12 and YMG26 were auto-flowering *Cannabis* varieties. Under long-day photoperiod conditions, DMG12 and YMG26 automatically switched from vegetative growth to the flowering stage ~70 days after planting. To benefit vegetative growth, the plants were cultivated under long-day conditions with day/night temperatures of 26/23°C and a photoperiod of 16 h light followed by 8 h of darkness prior to anthesis. Since *Cannabis* inflorescence can develop under short days after anthesis and cultivation under long days can get quite expensive, it was changed to short-day conditions – a photoperiod of 8 h light followed by 16 h of darkness after flowering – which was also the preferable choice in *Cannabis* commercial production. Plants were individually potted in a greenhouse, with each pot measuring 10 cm in diameter and 12 cm in height. We characterized the productivity and traits associated with yield components of these individual plants, as well as the number of flower nodes on each second-order branch. Since producing seeds can decrease cannabinoid contents, plants for panicle weight measurement were grown in another greenhouse to prevent cross-pollination, and the flowers at each branch were collected and weighed. Panicle weight was represented by the mean of the weight of the dried flowers in each branch plus 15% standard moisture. We calculated grain weight per plant as the average weight of dried grains adjusted to 15% standard moisture content. The 1000-grain weight was determined three times for each treatment. Morphological traits were assessed manually.

### Generation of transgenic plants

The cDNA of *CsMIKC1* was cloned using the primers CsMIKC1-CDNAF1 and CsMIKC1-CDNAR1 (Supplementary Data Table S1), and then transferred into the pNC-Cam3304-MCS35S vector from the laboratory of Dr Yan (ITBB, CATAS) [37]. The construct was transformed into DMG12 as the host plant but not into YMG26, because it has a low regeneration rate and is currently not transformable [9]. The expression level of *CsMIKC1* in the positive transgenic plants was measured using qRT–PCR with primers named MIKC1-rt-F1 and MIKC1-rt-R1 in Supplementary Data Table S1. The pG41sg (harboring the developmental regulator genes *CsGRF3* and *CsGIF1*) constructs were used for genome editing [9]. The gRNAs targeting *CsMIKC1* and *CsBPC2* were designed with CHOPCHOP (online software, https://chopchop.cbu.uib.no/). The gRNA primers were designed based on *Cannabis* genomic characters as well as the scores of potential off-target sites (Supplementary Data Table S1). Two pairs of primers (CsCAS9F2/R2 and CAS-TEST-F6/R6; Supplementary Data Table S1) were employed to detect positive plants harboring the genome-editing construct integrated into the *Cannabis* genome. Subsequently, positive plants underwent sequencing to identify deletions or insertions in the targeted region. Considering that developmental regulators may have some effect on inflorescence morphology, we modified the methodology and selected successful mutants without developmental regulators in the progeny. In the generation of transgenic plants overexpressing *CsMIKC1*, developmental regulators were not combined into the vector, and hence the positive individuals did not contain these developmental regulators. *T*_2_ transgenic plants of Cas9-mutants were stabilized as homozygotes by inducing sex change and self-pollination, and these homozygotes were used in relevant analyses. The genomic sequences of *CsMIKC1* and *CsBPC2* were amplified using PCR. Mutations were detected in the two alleles by deep sequencing. In addition, *T*_2_ transgenic plants overexpressing *CsMIKC1* were confirmed by qRT–PCR and evaluated in the analysis.

### Transient promoter activity assays

The *CsMIKC1*-Prom construct includes 320 bp before the start codon of the DMG12 allele. The *Csmikc1*-Prom sequence included 320 bp of promoter sequence with a poly(T) sequence instead of the (CT)_8_ repeat from the YMG26 allele (Fig. 3a). The *CsMIKC1*-Prom and *Csmikc1*-Prom constructs contained the 320 bp sequence, differing only in a 16-bp sequence. The CaMV 35S promoter was used to drive the LUC gene, serving as a control to estimate transient expression efficiency. The 320-bp fragments from DMG12 and YMG26 promoters were fused separately to the uidA gene, which encodes GUS as a reporter. These constructs were then cloned into the pNC-Cam3304-MCS35S vector. Primers used for cloning are listed in Supplementary Data Table S1. The *CsMIKC1* promoter includes the (CT)_8_ repeat from the DMG12 allele and the *Csmikc1* promoter includes a poly(T) sequence from the YMG26 allele, which was the only difference between the two fragments. Internal control for the CsMIKC1::GUS assay comprised the pNC-Cam3304-MCS35S construct. This construct contained the 35S promoter fused to the LUC gene. The LUC gene was cloned using primers LUC-F1/LUC-R1. We transformed the 35S::LUC construct together with the *CsMIKC*1-GUS or *Csmikc1*-GUS construct into *Cannabis* protoplasts. These protoplasts were isolated from SAMs using an enzyme solution that consisted of 30 mM 2-(N-morpholino) ethanesulfonic acid (Sigma), 3% cellulose (R-10, Yakult), 20 mM CaCl2 (Sigma), 15 mM KCl (Sigma), and 0.6 M d-mannitol (Sigma). The meristems were incubated with shaking at 160 rpm for 10 h at 26°C. PEG solutions were prepared for transfection, and consisted of 0.3 M mannitol, 35% PEG, and 200 mM CaCl_2_ [38]. The ratios of GUS to LUC were utilized to determine relative promoter activities. After transformation, the protoplasts were incubated at 26°C for 36 h in a 2-ml centrifuge tube containing lysis buffer with 0.8 mM 4-methylumbelliferyl-β-d-glucuronide (Thermo Fisher Scientific Inc.). To terminate the reaction, 0.15 M Na_2_CO_3_ was added to the reaction after 40 min. LUC activity was assessed using the Luciferase Assay System E4550 (Promega), while GUS activity was measured with a Synergy H1 reader (Agilent Technologies, CA, USA). The GUS/LUC ratio was calculated as (GUS_40 min_ − GUS_0 min_) × 10/LUC.

### Quantification of gene transcript levels

We extracted RNA samples from transgenic and non-transgenic plants cultivated in the greenhouse, including root, stem, SAM, female flower, male flower, and leaf tissue from adult plants. Samples were processed to extract total RNA using the EASYspin Plus RNA kit (Aidlab Biotech, Beijing, China). The extracted RNA was utilized to synthesize cDNA employing the SuperScript II Reverse kit (Thermo Fisher Scientific, CA, USA). The gene transcript levels were determined by qRT–PCR with specific primers (Supplementary Data Table S1) in a CFX Opus 384 Real-Time PCR System (Bio-Rad, CA, USA). The level of *TUB* expression was measured as an endogenous control for normalization of qRT–PCR data. The primers to detect the expression of the *Cannabis TUB* gene can be found in Supplementary Data Table S1 [1]. Gene transcript levels were quantified utilizing the 2^−ΔΔCT^ method, with CT representing the threshold cycle [39]. The cycle difference between the target gene and *TUB* gene was first calculated and then the second difference of the first calculated ΔCT value between a sample and the selected control sample was calculated, which was the ΔΔCT value.

### *In situ* RNA hybridization

The cDNA encoding the complete CsMIKC1 protein was amplified with primers CsMIKC1-CDNAF1 and CsMIKC1-CDNAR1 and cloned into pGEM-T Easy vector. This plasmid was sequenced to verify identity. For *in situ* hybridization, digoxigenin-labeled RNA was produced based on the instructions (Roche). The plant tissues were fixed in 0.15 M sodium phosphate buffer containing 0.15% Tween-20, 0.15% Triton X-100, 4% paraformaldehyde and 0.25% glutaraldehyde. SAM, female flower, and male flower tissues were fixed and embedded in Paraplast Plus (Sigma), sectioned at a thickness of 15 μm, and mounted on poly-l-lysine-treated slides (Thermo Fisher Scientific Inc.). Hybridization together with immunological detection were conducted following previously described methods [40].

### Yeast one-hybrid screen

The tests were conducted following the method described in reference [41]. Promoter fragments of *CsMIKC1* and *Csmikc1* were amplified from the genomic DNA samples prepared from DMG12 and YMG26 (Supplementary Data Table S1). We fused these fragments into the pAbAi vector to generate the bait constructs. The plasmids were then digested with the restriction enzyme BstBI and integrated into the yeast strain Y1H Gold using Yeast One-Hybrid kits (Clontech). Bait constructs were used to screen the Y1H prey library constructed from the Chinese commercial cultivar YUNMA 8, which has been widely cultivated in the Yunnan Province in China. The transformants were cultured on selective medium (SD/−Lue + 1100 ng/ml AbA). The growth ability on medium can be judged by detecting the positive binding between prey and bait colonies. The positive colonies were confirmed and sequenced following previously described procedures [41]. Positive control colonies were generated by transforming yeast cells with both pAbAi-p53 and pGADT7-p53 vectors (Clontech). Negative control colonies were established by transforming the yeast cells with the pAbAi-*CsMIKC1*-Prom vector together with the empty pGADT7 vector.

### Yeast two-hybrid screen

The full-length cDNA which encoded the complete CsMIKC1 protein was amplified by primers CsMIKC1-CDNAF1 and CsMIKC1-CDNAR1 (Supplementary Data Table S1), then cloned into the pGBKT7 vector. The vector was utilized in screening the Y2H library. The cDNA sequence was used to test autoactivation. We transformed the MIKC1-Y2H construct into the yeast strain Y187, which was used to screen a Y2H prey library constructed from YUNMA 8 as a bait, then incubated for 4 days at 32°C. Positive colonies on the culture medium were screened, confirmed, and sequenced. Fragments of the positive cDNA clones were queried against the NCBI database using BLAST to identify the proteins interacting with CsMIKC1. The interaction between CsMIKC1 and CsVIP3 was confirmed through co-transformation three times in the Y2H system [42].

### *In vivo* protein interaction between CsMIKC1 and CsVIP3

The subcellular locations of CsMIKC1 and CsVIP3 proteins were observed in tobacco leaf cells by expressing them using a pEG101-YFP vector. To analyze subcellular localization, cDNAs were cloned into the pDONR207 vector and then transferred into pEarleygate101 vector (pEG101) using the BP and LR cloning kits (Thermo Fisher Scientific). *CsMIKC1* was cloned from the pDONR207 vector and fused into the pEG202-YC vector. pEG202-YC encoded the C-terminal region of YFP. *CsVIP3* was fused to the sequence in the pEG201-YN vector encoding the N-terminal portion of YFP. We co-transformed the *CsMIKC1*-fused pEG202-YC vector with *CsVIP3*-fused pEG201-YN vector into tobacco leaves to test the *in vivo* interaction between the two proteins. Primers for amplifying cDNAs are listed in Supplementary Data Table S1. Tobacco leaf disks were prepared and imaged following a reported protocol [42].

### Silencing of *CsVIP3* via transient RNAi expression

The pNC-Cam1304-RNAi vector from the laboratory of Dr Yan (ITBB, CATAS) was used within this study. The cDNA fragment of CsVIP3 was amplified with CsVIP3-RNAi-F1/CsVIP3-RNAi-R1 and cloned into the pNC-Cam1304-RNAi vector following the protocols reported previously [37]. We transformed these vectors into *Agrobacterium tumefaciens* strain AGL1 (Biomed^®^, Beijing, China) using a previously reported protocol [9]. Stipule segments (a component of the female flower) were taken from fully expanded flowers and immersed in an AGL1 suspension for 4 min at 500 mbar under vacuum pressure. The stipule material was washed with sterile water and placed on moist filter paper in a Petri dish. The Petri dish was then placed in a controlled environment room at 26°C with a 16-h photoperiod for 2 days. We extracted total RNA from the stipules and synthesized cDNA following established protocols [1].

### Design of the phytohormone experiment

One week after anthesis, the BPC2-ED2 mutation line, the MIKC1-ED7 mutation line, and non-transgenic DMG12 plants were selected for the phytohormone experiment. Eighteen plants were selected for each phytohormone treatment (six plants from each line). Solutions of Ethrel (synthetic ethylene, 100 ppm), ABA (100 ppm), and BA (100 ppm) were prepared with demineralized water. Plants treated with demineralized water served as the control group. Spraying was performed in the early morning, with each plant receiving three sprays of equal solution volume at 8-day intervals. Three flowers from each plant were sampled randomly, and the relative expression level of *CsMIKC1* in each data set contained a total of 18 biological samples. The flowers of each DMG12 plant were harvested 4 weeks after anthesis and panicle weight was represented by the mean of the weight of the dried flowers in each branch plus 13% standard moisture. Flowering time was defined as the time the first solitary flower developed in the axils of leaf petioles. Phytohormones were applied when the first foliar bud emerged, which was 8–10 days earlier than flowering for DMG12 and YMG26.

## Supplementary Material

Web_Material_uhae161

## Data Availability

The data supporting this article are accessible within both the article itself and its online supplementary material.
