# Supplementary material for: CsMIKC1 regulates inflorescence development and grain production in Cannabis sativa plants
Source: Hortic Res. 2024 Jun 12;11(8):uhae161. doi: 10.1093/hr/uhae161 (PMC11298619; doi:10.1093/hr/uhae161)
Supplement: Web_Material_uhae161 [file web_material_uhae161.zip › supplementary figures V4-clean copy.pdf]

>a. *LOC115700576,* genomic DNA

GTCCAGAAACTTGAAGTCCAATTCTTTTTTAATTATCTCCACATCAATATTTACACAAACAAAGCACAAAGCTAGCTAAGTACACAGTAAAGC
AGCTGCAATTTTTTGTGGACAGTACATATTTATATATCTATATTTATATACATGAAATTACAAACAACAAATGAGAAATTCAAAGATTGATTAACA
AAAAAATATTAGAGTTTTTAATAAATATGATCGTATGTGCTGCATGCATAATACTATTATAACCACACTTATTCAACATTGAAGTTAAAGTTTAAG
TCCTGTTAAGGTAATTAAATAAAAAGGAAAACATCAAATAATAAATAACAAATATGAAAGTAATAAAATATTCTGGTGGCTAATTAAGTAGAGT
TCTTTGAAGACATAATTTAACTAAAATTTTCTTGTAATAAGATGTTAATTTTTTTAAGGGGAATACCATATGGATGTTTGTACGTCACTGATTATT
TATTGATCTACTATTATAGCTAGAATTTAATGAACAATAATAAACGTTTTTAAAGAGTAAAGAGTACATAATGATATCGTTAAAGGAAGGGAAG
GTATAAGATTGAGGAAGAAAGTAAAAAATGAGGTTATTCGAAATGGCAAGAGGGTCCCATCACCATTCTTATATCGACACGTACATGGCCCTA
AATGACAGGAATGTATAGGACTAGACTTGTCGTACTAAATCATTTTGTAGTAAATGAGTTTTAAAATAATCTTATCAGAACTAAAAAAGATTTAT
AATAAAAGATCAAAGGAAACTTTTTTATTAATGTGATCTTACGTGATGATTTAGTCAGAAAAACTGAGATTAATTACTAATAGTATAACTATATA
TATGACATCTCTTTATATATCAGCAACTATCAATAGGACACATTTCATTACATAAAAAAATCCTATACCAATAGGACAAAACAGAATTTAGTAAC
TTTGTTTTTCCTTCTA[CTCTCTCTCTCTCTCT/TTTTTTTTTTTTTT]TCTCAAAATAAAATTATCTATTATACATAAATATATGCGTTGGTTAGGGT
AAAAGTACGGCTGGGATAAGTAAAAGAGGATTGGAAACTTTACCCTAAAGAGGAGGAAGAAGAAGGAAGAAGGGGGAATATATAAGGAAC
TATATTTTATATATATAGTAGTTTTGCAGTAGAAGATTGGTGGTGTTAATTAATATATATATTTCATACTTAATTTGGAAATATATATACTATTTTTTAT
TTTGAAAGAACTATATAATATATATTTTATATATGGGAAGAGGAAAGGTGGAACTGAAGAGGATTGAAAACAAGATAAATCGACAAGTGACCT
TTGCTAAAAGAAGGAATGGGTTGCTTAAGAAGGCTTATGAGCTCTCCGTTTTATGTGATGCTGAAGTCGCTCTTATCATTTTCTCAGCTCGAGG
CAAGCTCTATGAGTTTTGCAGCGGTCCTAGGTATTGATATTTACATATCTATACATTCATTTTTTAAAAAAAATATATATCTTCTGGTTTTTCAATC
TTTGTGCTCCCCGCGGAGATAAGTTTTCTTATAAGTGATTCTACGTTTTAGGAATTAATCTGTTCGATCTGAAGATAAGAACTTCTCTCAGTTCC
CAATTTTGTCATCCATCGTATATCAACTCAGATGATTTTGTTTTTCTTCCATCTTTCCCAATTATATATATATATATAGATAAATTAATTCTAGTCAAGA
GAGGAAAGACAACATATATAATTTTTAAGACAGCTTTGAACATGTGGTTTTTCCACCAAGCTAGGTTGAGAAATTACATATAAAATATTGGTTG
TCGATTGCGAAGAACTACCTTTATATTTTTAAAATTTCATTTATATTGATGTTTAAATCGAGATCAGAAGAATATATTCGTTCCAAAGACAAAAA
AGAAAATATTACCCATTTGGCCCTAAAACTTCAAAACATATTTTTCCTACTCCACTTAATTACCATTTATATAATTTCACTTTCATTATCATGATCA
TGATTTTTGACAAGTGACCCATATCTTCATATGAATCTCTCTCTTCTACTTTGGCATACGGTATTCCGAATTTGAGTGTGCTCAGCAATTTCATTA
TATTTATATATATATGTATAATAGCAGCTGATTTACTAAAAAACTGATATATATATATATCAACATGCAAAATATCTTATTAATTATAAAATCAATAAG
TATGCATCTGTACAAGAATATATGCTATGTATACATATATGCTAATATATATACATATATATAGTTAAGAATGAAATTTTGTGATGTAATTGACATGC
TGAAAAATAAAAACTGCTCAATAATTGAACTGCTTGACCATGGTTATTTTTTTTCTTTAGAATAATTAGTTACAATTCGTAGTGAACATGGAGTT
TATGTGATGATCATTTTACCATTTGTGTATATAATCATATCTGATTGGCTAATAAAAACCTAATCGTACGTCAAACGATATCTATAACTTTAGAAG
AATTGGACCGAATAAGAAATTGAATTCTGTTAATTAGTTATTAGATGGTATAATGATGTATGATTTCTTAATGCCAATATATTTAATTTTGATTACT
CTTTTGACTTTGTAAATCAAGGGGATTTATTGTTTTGTCTATATAATGTATTAGTTCAAATTACCATCATTGCATTTCATTTGTTATTCTCATAGGA
ATAAATATGATTAGTAAATCTACTCAACAATATATTATATACATGTACTTAATTAGATACATCAGGTTGTTAAAGTCGTGATAACTTTTGACTTTAT
GGTTATATATATACTATATATATTTCAATTTCTTACATAGTACGTACTAAATTGTGTTTCAATCGTAATCATAAGGCATAACAATATAATAACTGAAA
GCTTAATTACTTGCATTGGTTTAGAAGTATAACAAATTAAGCAGTCATGATAGTATCTATAATTAAATGAACCATTCCTTATATATATATATATACTA
TATAAATGTAAGTGTTCTGAGTATGTACGGACAAGAGTTAACTTAGATATACTGAGAATATTACTAGTTTCATTTTGTTAACTTTGAGTTGAAAA
TTTAAACATCGAGTAGTCAATTTTCAGGGATTTATATTTGATCAAATATTTATTAGGTACTTGTTCAGCGACATATATATATATATATAATATATGTA
TATTTTTATATAGTATATATACAGTACAGTCGTTTGTCATCGCTTTTTGTATCAGACATGACAGTAGTGTGGAAAGAAATACTTGTATTATTGTTT
GTTTTTAAACTATATGTGTATGTTTTTTTTTTTTTGATTAGTTTTTCATCATAATAATTAATCGAGACATTTTTGTTTATTCAAGCATCACCAAGAC
GCTTGAGAGGTATGACAGATGCAGCTATGGAGCACAGGAAGGAAAACTGGCTGGAAACGACACAGAGGTACGCCGTACCATTATACGTGAA
GTGATATATCACTATCGTTAATTAATAACTAGTTTATACGTAGAAAGAAAGAAAAGAACAGAAAAATGCTTCTCGACCATGGGGTCCTATTAAT
TAAATTTATCTTTAAGTATTGGCACATAAATATGTGATCGACATTTTCTCTCACAAAGAGGATGACCAAATCCTAATTTCCGTCCCAATAAAAAA
AATTAATGTAATGTATATAGGTATGTATTAATTTTTTGCATATAATATATGTTAAACTCCATGAAAGACCTCATTATCTTTATTCATATATATACAGA
GCATCTATCAAGAGTATCTCAAGCTGAAAGCAAAAGTTGAGGTTCTTAGACGAACGCAGAGGTATGTTATTCAACACTTATTAAATAAACTCA
TTATATATATGTACATAAAAGAAAGTATGGAGCTTCGATTTTAGTATCAATACACGCTTGTTTGGTGTTTGGATTTTTCTAAACAAAAAATTTGC
CAGCGAAAAAGAAAACTGAGAAATTTCATGTAATTTTCTCTTTTTCTAATAATACATATGCTTGCAAAATTAGGTTTTTAACATTTAATTATTTA
AATCTGGTATTTCTTTTTTTAAGTTTGAAAAAAAAAAAGATATTTCTATTTTTTTTTTTTTTAAATCACCAAAAATTCCTGTGAATTTGATCTATAT
ATCTATATAAAAATATATATAATAAGCACTGAGTATAACGCCATTTTTGTTCAGGAATCTTCTCGGGGAGGATTTAGAGCATTTGGGACTAAAGG
AGATTCAACAGCTTGAGCAAAAACTTGACTCGTCATTGAAGCAAATCAGGTCAACTAAGGTACATAACATGAAGCTCCAACTTTTTTATTTTT
TATTTTGAAAGAAGCTTAAAGCTCCAGCTTATTGATTACAATAATTGGAATTTAGAATTATATATAGATATCTATGGAGTGAGAGGGGAGAGCG
GGTGAAATTGCAACTTATTGTTTATTAACATATATTTCTATAATTTTTTTTGCTGGAAATATATTTATATAATAAAGTATATAATAAAATGGAGAAC
GACGGAAAATATATCTATATATAATTTCAATATTCAATTTTTTCTATACGGTTAGCATATAATTGTATATATATATATTGACTCTTGACTAATTAATTATA
ATATGCTAGGACGTTGTAAAATTAGTGAATGCAATCCTGTTAAATTTTGCATCTAATTAAAAAACATTAAGTGGATTTGCATCTAATTAAAAAAC
TTTATTTAATGCAATCCTGTTAAATTTAGTAAAATAGATAATGAACGAATTAAACAAATGAGTGAGAACAGATTGCGTTTGCATGTTTCGTTTAG
ATGGACTTCCACCACTAGAAAACTATATGTGTACCTTTAGTGCATTCTTTTCAGTTTTGGCTCATGAAAAAATATAAATTTAATTTTGTTTAATA
ATATAACAGTGTGCAATATAATTATTTAAAATTATTAAAGGAGCTAAAATGAGTTTATTTAATCTGTTCGTTACACACGTAGGCCCGAAACTATT
CTAAATTTCTCCTCAATAGTAAAAATTAGAATTTACCAAAACATATCTATTTAGGAGTTCAATTAATAAAGAATTTTCAAAATGTATTCCATATTA
TTTGTTCTTGGGAGGCTTTATCAGTAAAGTATTATGTTGAAGCATTCGAAGAAACGAATACATCGATGTAATATAGGAATGTTCATATTGTAACC
GTTATATGGACATGTATAATACGTTATTTGGTTTCCATATTTTATTTACGTTATTGGGTTTAATTAGTATATTTTAAATTTATAATTATTTTCAAAATA
AAACTGTTAATATTTTATAAGGTGTCACCTTATTTATGGTGATGGCCGTATGTTGGGCATTACTGGTGAATAGTAATAATCTTATTATTTTCAATG
AAAATAGTCATTGAATAAAATAATAACATTCTTAGTTAAAACGATGATGCCATAATGTTATATACAATTTACTGGTTATTTCTGCAAATTACTAAT
AAGTGTACATATACATTACATATGTACCTACCATGGCTATATAGGACAACCAGCTTTTCCCTTCATCAGAATTAATTCTGTCATATATATATATTTTGA
ACGGATTCATGCATCTTGCATATATATACAATACTGTGCAAAGTAACAAATCATAAAAAAACCTGTTAACCAATTCTGCTGACCAGTACTTGTAAT
AAATATTCTGCCACACGAGCATAATTATTTGACGCAACAAACGAAATTTACATTCATTGATTTTGGCATACTATTTAAACTTTCCATGTTCATTTT
TAAATTAGCATCATACTATTCAAATATAAATGAATATTTTTTAAAAAAATTATATGTATTTATTTATTTTTGAGATAATATGTTTTGAAAATATCACT
TTTTATTATAGGATGATATTTATAGTATAGAGCAATGAAAAATTGAAATGTTTCATATATTTGACTAAAAGATTTAATTTTTGATAGGAAAGGGTA
CTACTGCATGGTCAACTTAACTAATTTATGTATATATGATGCAACAGCGAGGCCGTACGCTTGTTTTACATTCAGATTTCTTTATAAATTGAAGTA

CGTACGGATAATAAAGCTATAGTTATTAATTACACTAAAAGGTGTAATACTTGACCATAATCCATATATCTTTTAAGAAATTTTAGAAAAGACTA
ACACTATGTGTAGGAGGCAGAGTTGGGTAGATGGAGAGGAGAAGATAGAGAGATGAGAGGAGACAATAAATCCAAGGGATATAGTTAGATG
GACAGTAAATCCTTAATTTAAAAATTCTTTCATGCATAAATGTAAGAATTCAGTCCTTCCTCTTTAATTATTTAAAATTACAAATATACTCTCAAA
ATATATTATTAAAAAATTACAAATAATATAATAATTTTATAAATAAATACTCTTCATCCTTTTATCATTTCCTTTCCACCAAACACCTTAAAACATT
ACTACTCTCCTCTCCCTCCCTTTTATTTTACATCCTCTCTACCAACTCTCCATCCTCCATATTCTCCATCTTTCCTATATTTTATTTTCTTATCACAA
TAAATGTTTCCATCTTAATTAATCTAAACAAATGACGATTGCTATTACACATAGTGGTGCTCAATAATATTTTATAATATTATTAAAGTAAATTATA
TGAGATTTAATACTTATAACTAATTATAACAAATGACATATTAAAGCTTAAAAATTATTAGGCAAGAATTTCTTTTGGCATTTTTTATTTAGTAATT
AGCTAGTTTGAAAAAGCGTGCCTAAGTGTAGTAACTTCAAATTGATCTACCTAATAAATTCTCAAATCTTATATTACCCTAAAAACTAAGTTGC
TCTTACCAAAATAGTTTGTAAAACTAGTAACAAACTACCTACAGAATAGGTTGATGTTGAAACAAACAAAAAAGTCAAACTAACCAGAAAAT
AGTTGTTATTAATTTTTTTTTTTTTTTTTTTGGAGAAGGTTATTAAATTTGTTGAGTTACCTAGACGATTACAAATCAGAACATATATATGTTTATTC
TTAACAACTTAAATTTTTGGCATATATAAATTATATATTTAGTTACTTCGGAAAGTAAATAATAATGAAAATCTACGAAGTAAATGTGTCACAATG
AAAGTATATACATCTATACATTATAGGTACTTCAATTTAGTTATTAACATGAGCAAACAATTATAGGAAAATTAAATAAAATTATTTATTTGGATTA
TTTCTAACTTTTTTTATAGAAATAAAAATTATATAAAATATTCTTTATTTATTTCAGATTGTAATAACAAATATTGTAGATATATACATATATAGATG
AGACTTGGGAGATATATATATATAATCTATATCTACTCATCATTCTAAACCAATATAAATATGTTCATAAACCAAAATTTAATTAATTAATTGGAAC
AAAAACAATCTTTACAAAGTTAGTCCGAAAAATTACCAAGCATGCCATAAATACTGCAGGCAGGGGTACTGGTGGCAGTAATTTATGAACAG
TAAACATTCTAACAAATTAACTGTTATAAAGTCATCCAAATAGAACTACATATATATACATTAAATGTAAAATACGTACAGACCTTTTATTGAATG
GTACTATTGTTTGTCGAAGTAATGATTCTCTGATTCATAAAAAAAAAATGTAAAATACGTTAATATGGATTTTTCTTTCAATGATTTTATTGCAGAC
ACAGCATATGATTGATGAGCTTTCTCTTCTTCGGGGGAAGGTACTTATCTCTTTCTTATATATATGTAATAATGTGTGTTAATAATATGTGCGATCA
AATTATTATTATTATACATATAAATAATGTTAGTTAGTTTTGGAAAGAGTATTATATATATGTAGGTGCTTATGTAAGCATATCGAAGTCCTTGGTC
AATTAATTAATTTTTTCTAATCAAGGAGACTCATTATTATGGTTTAATCAATTTGCAGGAAGAGACATTGCTTGAAGAAAACAATGAATTGAAA
AAGAAGGTATATTTCAATTCTCAGACCTCGCTATAAGCCTATAAGATATAGCTATATATAACAATGAGAGAGCTATAGAAACTAGAAAGAGAGA
AAGAAAGAGTTGTGAATAAATGGGCTCATCGCCAAGCTAACTAAGAAGCCTGTTAACGAAAATATATCTATAACTATTTTATGAACCTTGAAA
CTACAAGATTAATATGTTTGAAATTTTTTAAAATTGGGATAAATGTAAGTATATATGTATATAAATGAACCAGCAACCAGAAAGTGACAGGAAC
ATGAAATGATAATCAGGAGAGATCAGAGAGATTAACAAATCCTTCCTTAAATATTATGTAGCTATTCGTAACCTTACTTATTTCTGTAGTACATTT
GTAAGTGAACTCATTTCAACATTCTTCTTTGCAGTACCATGCAATGCATCTTTGGCTTTATTTTATTTATTTTTTTTAGTGACCGTTGCAGTCGCG
TGTGACCTTCTTTTATTGAACTTATATAAAAATTACTGTATATTCTTATATATTTGTAGTGTTCCTCAAAAGAGTCGCCTATATTGCATATATATATA
TATATATATATCTACATATATATGAGCGTGCAGCCACTTTAGCTTTCGGGCTGTTTACCAAAAGGAGATTAAATACCAATGCTTGACAATTATATT
TATATATATATAGGTACTTGATCTAAAAACAAAAAATAATTAATAAATATTATACATACATAATTCATAAGCTATTTGATCTATACATATACCAACTG
TAATATTATATATTATGGATAGATATACATATAAATATTGGGTTTACGTTTGTTTTCCCATTCGTTCAACACTTAGAGAAGCCTACATTAGTTGTTG
GGTCATTCACTCTGATTCCTTGTAATTTTTCATATTGTTGAGCAGTTGGAGAAAATTTGTGGATTGATTCCTGAGCAGGAAAGTGAAAATGGG
GACCAAAATGTTGGATGTACCCACCATCCAACTCAGCCAGTGGAACTCAGTTTTGAGCATTTGCGATGCAATAATAATAGTGGCTACAATAAT
ATTAATACAAATAATACTACTTCTACTATTGCATTGCAGATCGGGTACTTACCTATATCATATTCTCTCTCGTCTTTATAAATATATTTATATACAAT
CTATTTATCTAATTTATTTCCTTTTAATTTTTTTTTCATCGATATATATAGCTACGGTGGTACTGTTAATTCAGGAATAAGAGGCCAAGAAAATATG
GGTGCAGCTCATTCAATGCAAAATTTGATGGCTTGTAATTCAGTGGACCGCGGATGTTTTGATCTTTAAAAGCCCTATACCTACTAGTATCAATT
TTACACTTTGTCTTGTTTTTTTGTTCCTTGTCTCCTTACTATAATGTTTTGTTCTTAAAAGGGGGGCAACAAATAAACTGTTTCTTGTTTGTGTA
CTATATAGCTATCTTCTGCTATTGGAACTATATATATTAAAAAAAAAATTCTATGAATAGTCAAATTATATTTATATA

>b. *LOC115700576*, MADS-box protein CMB1, mRNA

ATGGGAAGAGGAAAGGTGGAACTGAAGAGGATTGAAAACAAGATAAATCGACAAGTGACCTTTGCTAAAAGAAGGAATGGGTTGCTTAAG
AAGGCTTATGAGCTCTCCGTTTTATGTGATGCTGAAGTCGCTCTTATCATTTTCTCAGCTCGAGGCAAGCTCTATGAGTTTTGCAGCGGTCCTA
GCATCACCAAGACGCTTGAGAGGTATGACAGATGCAGCTATGGAGCACAGGAAGGAAAACTGGCTGGAAACGACACAGAGAGCATCTATCA
AGAGTATCTCAAGCTGAAAGCAAAAGTTGAGGTTCTTAGACGAACGCAGAGGAATCTTCTCGGGGAGGATTTAGAGCATTTGGGACTAAAG
GAGATTCAACAGCTTGAGCAAAAACTTGACTCGTCATTGAAGCAAATCAGGTCAACTAAGACACAGCATATGATTGATGAGCTTTCTCTTCTT
CGGGGGAAGGAAGAGACATTGCTTGAAGAAAACAATGAATTGAAAAAGAAGTTGGAGAAAATTTGTGGATTGATTCCTGAGCAGGAAAGT
GAAAATGGGGACCAAAATGTTGGATGTACCCACCATCCAACTCAGCCAGTGGAACTCAGTTTTGAGCATTTGCGATGCAATAATAATAGTGGC
TACAATAATATTAATACAAATAATACTACTTCTACTATTGCATTGCAGATCGGCTACGGTGGTACTGTTAATTCAGGAATAAGAGGCCAAGAAA
ATATGGGTGCAGCTCATTCAATGCAAAATTTGATGGCTTGTAATTCAGTGGACCGCGGATGTTTTGATCTTTAA

>c. LOC115700576

MGRGKVELKRIENKINRQVTFAKRRNGLLKKAYELSVLCDAEVALIIFSARGKLYEFCSGPSITKTLERYDRCSYGAQEGKLAGNDTESIYQEYLK
LKAKVEVLRRTQRNLLGEDLEHLGLKEIQQLEQKLDSSLKQIRSTKTQHMIDELSLLRGKEETLLEENNELKKKLEKICGLIPEQESENGDQNVGC
THHPTQPVELSFEHLRCNNNSGYNNINTNNTTSTIALQIGYGGTVNSGIRGQENMGAAHSMQNLMACNSVDRGCFDL *

>d. *LOC115701144*, genomic DNA

GGTCATGGATGAGACCTCAGTCTTTAACAAATTCATTAATCAACACCTTGTTGATACATCCCAGTGGCACAAGCTCAAGATTGTGGAGGATTT
TGTGGTGTTATGATCGATCACTTGCCAAGCTCAAAACTGATGATAGTTTTGTTAGTTACAAGTCAATTAGTTCAAAATGTATATAAGTGTGTCC
CAGTGTCTAAGTTTTCAGTTAATGTGGAGCACTAGAAAGTTAAGAAGTTGATTGTTTGAGTTACTTTGTGTTCCTAGATTCTTGGATATCCAAG
TTGTAATATCCGAGTTGAGATATATATTCAATACAATACATTCATTTATGTTCTATTTTGTGATTCTTTCTTCTTGATTGATTGAGTTATTGGAGCA
AGGTGGGATTAAGTTTACTTGATCTTGACATTAATGTGATGTATTTTATCGCAACAGTATAATTAAATATATATCACAAGTAATTACTGTTTTTTG
CTTACCAAGGAAATTATTGCCCTTTTTTTTTAAACATTCGAGAAAGAGCCAGTACAGTCCCTTATGACGACCTATAGACTCCTCATGTTGTTGT
TCTTTGATTTTTTTATTTTTTATTTTTTTGTAACTTAAGGGGGGAAATCGTTTTAGAATAGGATTGATGTATACTTGGGTAACTTTTAATGATACTC
TCGGAACTCGATATTTAATTAAATTAATAGCGATATTAACACGTAAAAGGATGCTTGGTACTACTGGTGTATTTTAGCATTTTACTAAAATTAATA
AACAAGAGCATTGTTATAAGTTACTAAGGGTATTTAGTACTATATAAGTGACGTTTTATGATTAATTAGCGATAATTTTTTTAAATTAATTAGGAT
ATACTATTAAAGAAAACCCTAGAAGTTAGTAATAGATTTTAACAATTCTCAATAAAAAAATAATAGGAAGAGTTCGTACTATATTTTTAAGAGTAA
CTAAGGAGTTCGTACTGATATTTTCAAAACAAGATCGAGTTCGTACTGATATTTTCAAAACAAGAGTTCGTACAGAAATTGTTAACGCCAGGG
AAGGCATAATAAGATTGAGCAAAAGAAAGTAAAGATTAAGATTCCCATCGCCAGTACCATACTCAGTTCGACACGTATACAGCAGCCCTTAAA

GGCAGGAATGTAAACGACTACAACTGCGTGTATTTAAAAAAGTTAATCATTTTTATTATTATTATTATTATTGTATACTGACATTATTTTTGAATTG
ATCAAAGGATTGAAACTGCTTTTCTTGTGGTGATGTTACTTACGTGTGTCATGATATGGACAGTACTATATCTTTTCTGACTTCTTACATATATAA
ACAAACTACTAATAAGACAACTTGACCTCACAAAAAAAACAAACAAAGCAACCAGCACTACAAAAAATGGACTTTTAGTGAGAATTTTTTA
ATCAAAACATATTTTTTTTAATTAAATGTGATTTTTAATCATCATAAAAAATTTATTTTGTGATTAAAACATAAAATTTAGATACAAATTATCATTA
ATTTAATTTTACTAACAACCAATATTATAACTTAAAACATATATAGTCACACCAAATTATAATTTTTGTAATTAATACCAACACACATTTTAGTCAT
AATATAAGAATGATGATAATTTATAATTAGTCACAATTTTTTTCACTTTTAATCACAAATTTTATTGTGACTAAAAGTAAAATTTTTGATAGCGCA
TGTTGTGTAGTAGTAAAATTGGTTTTGTTTGGATTAAGTGTTCGAATTTTATAACAATTCAATTGTAAGGAATTATGTTTATTCTTTTGGGAGAA
AATTTTGTTGTGGAAGTGTTATCTTTAATAGAATGAAAATCCTCATGGGATACATACTCATAATTTAATTAAAGCATAATAAGGCATAAGACTAC
TATAGTTGATACCTAGATCAAACATTATCAAAGCTACGAAGAGGTGGTGAAGGCAATATTTACACCGAATGGTTGCCTTACTATTAGAAAAAA
AAAACAATAGGACAAATATAATTTTTTTTATATATATAAAAAAAAAACTAATTAATGAAAATAATAATAGTAAACCTACCAAGTACCCATAGTAAT
ATTTGCTTCTTGTCTCTCTGTTCTTTTTGGAAAATTAAATATCTATGCTAATGTTATGGTATACGGTAGGTAAGAGAGGATTAGAAAACTTTAAC
CTAAAGGCCGGATAAGAAAAAGGCATAAGGCTTATACAAGAAACTATATTTATATATATATATATATAGTAGTTTAGCATATGAAGATTACTATATAC
ATTTAAATTCATAATTTGGCTATATAGAAATATATATTTTTATATTTGAATATGGGTAGGGGAAAGGTGGAACTGAAGAGGATTGAAAACAAGAT
AAATCGACAAGTGACCTTTGCTAAAAGAAGGAATGGTTTGCTGAAGAAGGCTTATGAGCTCTCCGTTTTATGTGATGCTGAGGTTGCTCTAAT
AATCTTTTCTGCTCGAGGCAAACTCTTTGAGTTTTGCAGCGGCCCTAGGTATATATATTTTCTTCACCCATATATATATCATGATTTTTTATATTAA
GTATGTGAAAGATTTTTTATTTTATTAAAACGAATATGGCCCCGGGAAATATAGTTATCTACTGATTTTAAGGAATTTTTGTGGCTTGAAGACTA
GTAATTCTTTATACAAAGCTGATCGATCACCCAGATCAGGAACTCAGAATTTTTTTCTAGGTTATAAAATCAATTCCATCTTTAACTAATATGGT
TTTTCTAAAAGTATATATCCTTTTAATTTATATTATATATGCATCTATAATGCATGTTTTAAACAGTTTTTTTTGACGGGATTGTTAAGGTGTTAGG
ATCGAGATATGCAGAATATTCGCTCCAAGGAAACAAAAAAAAAAAAAAAAAAAAGCCCCATTTGGCCCTAAAGCTGCAAAACTTATTTTATCGC
GACTTCTGCTTTTTCGTAAATATAATTTCAGTATATTTTTTTGTTTTGGCAAGTAACCGATACGCCTATCGATCTCTCTCTTAATCTACTTTACGTTA
TATTCGTTCGGTTACTAAATTTTTACATATTCTGACACACAATATCTTTAGTTATATTTAAATCATCAATTATATATGTGCTACGTATATAAAAATTA
AATGTACAGGATCATATATGTATATAGAATGTGATATATATTTATATATATTAATGTATTTTGGGTATATGCTCTTGGGTAAATGTCTTGAGCTGCGC
GTGGACGGGGACTTATAGAAAGTACTGGCGATGATTGTCATCACTGGTGCTTTTATACATAATGAAATTTGTTCCGATCGTGACCACACCCATT
AATAAGTTTCTAATAGTTCTAACAAAATAACAATGTATAAAAATAATATAAAAACTAATTGATTACTTTACAAGCTTTACCTGCCCTAGAAACTC
ATTCTAGGCGCCTAGTGCTTAGATTCTCTGAACCAGAGTAATAACTTTCACTATTTTCAAATATGTATCTTACAAATATACAAACAATAATGTAA
ACAATACACTCTTTTAAATCCATACCCATACCAACCAAGACTCTTGATTGGAGACACAAGATCCCCTTGTGTGAATGATGAGCTCTCTTCAAC
ACTTTTCTTCAATCTGAACTCCCTTCAGATCTGGTGATAAAGATGATACTGAAATTCCTTCAGTCTGAACTCAACATCATCAAGCTCTGAACTG
CAACTTTGGATCAATACCACCAAGCTCAAGATCACCTGCCATGGAACGTCATAAAGACGAAACAAGAAGAAAGAAACGTAATCCCTTATCTC
CGATTAAAAAGCTTCCTCAACATAATACCAAGAAGTTATAATTATACCCAGCTAAACCAAGAATCCCGAAATAGATAGCTGTTACCATCTGAAC
AATTAAAACCCAAAATATCTTTGCTGACGTACACTAACAGTACAAACTCCAGACAATACACTACGAATCCCAGACGCAACTGTACATAATTTG
TCACTACATAATATACTGGTTCTTACAGACACTAGTTTGGATTCTCATAATTTGCAGGTTATCTCCACCTACTCTAACTAAACAAATTAAAGAAT
TAAAATTTAACATATGAATTTTAACCCTCATCAGTAAGAAACTAAAAATTACATGCATATGCTATCTTCTTCTTCTTTTTTTTTTTTTTTTTTTTTGA
TGAAAGTGTGTTTCAATACTTAAAACTCTTTTGTACAAATAAATAGCATTCTCCATGAAAAGGAGAAACCTATACAAGAATAATCTTTACAAGG
TGTCTTACCATTTTTTATCACTAATGGTTGTTTTCTTTGGCCATGTACATGTGATTCTATTTTTAAAAATATCTTTTACTTTGATAACTATACTATCT
ATACTTGTTGTTGAGTTTTGCCACAAAACATCATTTCTAGCATGCCAACATTGTATATAAGAGCTGCAATGACAGCAGCTAACATCATTTTTTTA
ACCTTGCTAACCTTAACTTTTCGGATCAATATTACAATTCGGTGAAGGGAATTGGCAGCCATCTTTCAATTAAGCCATTCTTTGATCTTCTACAA
GCAGCCCAAAGAGAAGGGACATTCGAAAAAGAGGTCCTGCACTATTTCTTCATCAGCATTGCAGAAAAAATACCTTGTATTTTTGACTGGTTT
ATACAGCTGCAATCTATCCTTTTTCTTGAGTCTATTTAGCATGGTAAGCCAAAAAAAAAAAATAAAACTATGTTTAGGTAAGTTTAGTCAGCTCC
AAATTATAGAACTCCAATCAACTTTGCTAGTGAAAAGGTTGAAGAGCATGAAAGCCAACAACCACTTTGTAGCTGTCATTTGAAGCTTGATTC
ATGTCAAACAACTATTTTGCTCTATCCTTTATAGCTACCATTTTGTGGGAATACAAGTTGGTTGTTGGAGTGGTCTTATAAGACCACCAATTCTC
CTCCTTTATCTACACATTGTACACCCACTTCACCCATATGTTGTCCTTCTTTTTAGCAATGGCCCAAACATACTTGAATATAACTGCCATATTCCA
ATCGGAAATGCTTCTAAAACCCAAACCTCCTGAAGATTTAGTGAAGCAAATTGTGCTCCAGGCAATTACTATTGAACCTGTAAGTATATGATTT
CCATTCCTCAAGAAACTACGACAAATAGATTCTATTTCCAACACAATCTTCTTTGGCAAAAGCATGACTTGACACCAGTAGGAATGAATGGCC
ATCAACACTGAGTTGATGAGCGCCACTCTCCCTGAAAAAGACAAGTTTTGGGTACTCCAACTTCTTATTCTTGCCACCATTTTTTCAACTAAG
GTACCACACTCTGCTGTTGAGATTCTCGTAGGACATATTGGAACACCTAAGTACTTGAAAGGTAAATCGTGTTTCTTGAATCACGAAGCATCA
AGAACTCTTTGAACCTTATAGTCACTCATATTATTGCAATAAAAGACAGTTTTAGATGGGTTAGGAAATAAACCTGAAGTTTGTGAAAATAATT
TGAGCCTTGGAGCATGAGGTAGATGCTTTTGGAATTTCCATTGCAAAAGAGTAGTACATCATCCGCAAAGGTCAAATGATTTAGCTTCAAGTC
ACCAGATCATGAAACATAAAGTCTTACCTCTTCCTAATTTTCTGCATGATGCGGGAGAGATATTCCATGCCTAAGACAGAAAGAAGCGGAGAC
ATCAGATCTCCTTGTCTCAACCCTCTCTTGGATTCAAAAAATCCATGCAAATAGCCATTAAACATCAAAGAGAACTGAGGAATTCTCACACAA
GTCATAATCAACTTAATAAATTTTGGAGGGAACTGAAAAGCGTAAAGCATCTCTTCAATAAAATCTCATTCAATAGTTTCATATGCGTTTGGAG
GTCCAATTTGAGCATATAGTTTGCTTTTTTAAACTTCCTTCCACAGTGTCGGATACGGTTTTGGCAAATCATAATGTTGTGACCAATAAATCTAC
CTTGAACAAATCCTCCTTGATTTTGAGCAATTAAACTAGGGAGGACAGTTTTTGAGCCTTGCGCAAATCAACTTTGTAGCAGCCTTGTAAAGT
ACATTGCAACACGCAATAGGTCGATAGTCTCTAACCAGGTTTGGGCACTTAGTCTTGGGAATAAGCATCAAAACCGTGCATGTTATCTTCATTT
ACTATATTTTTTCTGCAATATATTCATTTTCTTTGTTTATTATAATCCTCATAATGTTAGAGAGTCTTATATATATTAATGATATGTAAAACATAATTA
ATATGTACAATTTATAAACTTATTATACTATTAATAAGTTTTGAGATAGAACAATCTACTTATATCCTATTTATCTAATTTCTAAACCAAACTTCCA
ACATGGGCGCCCACAAGAAAACCTCCTAACACGAATTTGATTTAATCCAAAAATATTCACTACAAAAAATTATTACTTTTTTAGTGACAACTTT
TTGGTCACAACATTAATTTTTTGTGACAAAATATGACTTTTAGTAATAACAAAAAATTATTTGTGACTAAAATATAGCATTTAGGCACAAGTTGT
TACTAATTTAGTTTTAGTCACAAAAAGTATTGTGACTAAAAATACATTTAGTCACAATAAATTATAATTTTTGTGACTAATACATTTAGTCACGG
ACCTTTTAGTCACAACATATGAATAATAATTTATAGTTAGTCACAATTTTTTTTATTTTTGGTCACAAGTTTTGTTGTGACCAAAAGTAAAATTTT
TTGTAGTGATTGATTCAAAATGTAGAATTAAAAAAGTCATTTGCCATTAGGGTATATAATATGAATCAAAATGAGCCATTAATTTCAGACTGATA
TGTTCAATATTTTGGCAAGCAAAAATTAAACTATCATCTGCATATATAGATGATAATAAGCATTTCCATATATATTATATTAATATATAATGTTTAAA
AATTTAATACCCTAAATAAGCCCTTTATTCATCCTCTCAACTACGTGTAGATACCTTTCAGCAAACTTTTCTCTAGATGCTTTCTTTTAAGAAAC
TACCCCCAGTTCTCCACCGGTCAAACCAAACACACTACGCGCATTAATAATATATGTGAAATAAGCTAATTAGATATATAGTCTTATATAATTAG
AAGTAAAAACATTTTAAATTTGTTTAATCATTATTACATTACGTTATTAAGTTATCATTAAACACACTAATTAATTATCACTGTCACACTCTCTATA

ATAATATTTTCTTCATTCACTTCCTCATTTTCTGTCAATATGTGCGGGCAACGATATATTAATTGCATCACAGTGACAAAACCACAACTAATAAAT
ACTACATTGTGTTTCAATAATAGTCATACAGAGAAAGCTTTAACATACGCATGGTCGTCTTTCAAGAATTCAAGTTAGAGGAACTATAATCACA
TGTACCCTGCTGCATTATGAATTAATTTATTGTGATAAATAAATTGTCATGTGTTATGGTAGTTTAATTTGGAATGAGATATAAAAATATTTGTGTT
AAGAAGAATAGTGTACAGTAGAAATAACATTTTTTATTTGGCGATCGATTTGTTGTTTCCTACACTACCATATATTAGAAACACTTGGGGAGGT
ATATATGTTTAGGGGATTAACCACTGTACAGTACTATACATGTGACTAGTAGTATAACTAGAAAGTTCTATAAATAGTCCTTCGAGTCAGAAAAA
AAAGTTCTACTGTAAATATACTAGAAATATTTATAAATTTATAAGTTGTTAAACTATTATATGTAGTGTAAAACATATTATGTTAATTTCACATAGT
AATTAATGTATCCCAACTTCAAAATATGAACAGTTTATTTTTAGTAAGTTGGTCTTTTAATGATACAGCAGCCATATGAGTAGTATTTTATGTGAT
TATATTCACATAGAATGAATAGTAGAGTATTGAATCATGATTATATCTTCCGATCCGTACCGTAGCTATACTGACTGTATATAAAAAAAATAATGT
AATATGACACTAATTACTTTTTGTTAGTTTTATGTACAGAATTTATACATTCAGCAGTAGACTCGATCGAGTCGTATATATATATATAAATATCTGT
CTACTATTATCATTTTAGTATGAGTCCATGGACAGCAACATATAGCTTATGCTAGTTTTAAACTAATTTATAATGTGTATTATATTATTTTATATTTTT
CAGCATCACCAAGACACTTGAGAGGTATGAAAGATGCTGCTATGCAGAACAGGAAAGCAAACTACTATCAGGAAAGGACATAGAGGTACGT
ACAAATACTTACATACATATGTATTTTTTTTTTCAAAAAAAAAATAGCTAGAGTATTCCTTTCCCCTAATTAATCAGTGAAATATTTTTCTAAACAT
ACTATTAATTTTAGGAAAGTTTCAAAAAAATTTATGGTGGCAAAAATAACATTAGAACAGCTTTTATTGTATTAACGAGAGCCTAACTTTTTCTAC
TCATCAAATATAAGTTAGAGATGTACTCAATAAAATATATATTTATATATAAAAAAGATTCTCTAACTTAATATATACAATATATACTTAAACATCTG
TGATATAATAATAATAGTAGGGATATGTGCACATAATGGTAATTATATAAGTTATTATTTTTATTTAGACTGGATCTCTATATTTGTGTATAATTTATA
TGTACACACATCATTATTCCTTCCAACCCGATCCTATAATTTGTTAATATATATATGTATCGTTTTCGTGAGGTAGTATAACTTTATGATTTTTTTT
TTTTTGATAACTTAACTTTATGATATTTATTATTGTCTCTATATATATATGTACCCGTATGCTGTACTATTATCAGAATTTAGAAATTAATGCTATTTA
TTTATATCACCCCAATTTCAAGTGGTCATTATCATACATACAGAGTACCTATCAAGAGTATCTAAAGCTGAAAGAAAAAGTTGAACACCTTCGA
CGTACACAGAGGTAATTCAAACTCTTATGAGGAATTTATTATCTGGTTTGGATTTTTGCAAATTACAATCATGTAAACCTAAGTTTTTTTCAGGA
AGATTTATATCCTTGTATATATTTAAACTAAATGCATGAGAGGTAATTAATTCAACATTTATATATGGTTTATAACTAATTTAAATACGTGATTGAG
TTTAAAATTTTCTTAATATCAGTCTTGTCAACTAAAGTCCACCAAATAAACCCTTACACTAAATAATTCCTTATCTTTTTAAATCAAACTAATTAA
TATACATATATATATATATATATGGGTGTGTGTTTGGAGCTCTCATTAATTATGGTAGTATCATAAGCGCGCCTTTCCATATTTTTCTAAATGAATAG
TTTGGATAAATTGTTGTATCATTATCTTCTTTTATCATACATATGAAATTATGAATGTAAAAGTTTTTTGATATCAGAATATTTAAGTAAAATTCCC
TCACAAGGGGCAATAGCAAAAACAGTAAAATAAATAAATAAATTGAAGGATAATAGAATTGAGGTTTCCATATAATTTCTTACTCACAAAAAC
TTTGCTGTTGGCTACAGTAAATATATATAAACATCGGAAATTTTTATATATTATATTATTTTTAATCAATAGATTATGGCGTCATTATTTTTGTCTTT
ATAGCACTAAAATTATAATCCAGTATACCAAATTAAACATTGCGCCAGTTTTCTGAATTTATTGAATAATTTTAATTTATTTTAATCAGTATATAAG
TTTTTGTTTTTTGTACGTATATAATACACTTTTCAATCTTCAGGAATTAGAAAGTTGATACCGACTGTAAGTATAAAATGATATGCAAAATCAAAT
TGTATATTTTCTTGCAAGAAATTCTTGATTTGTGTTTTGGAAAAGGCTTCATTAATACCTTCTCAAATACTGGCTATTCTTTTTAGCAATAGCTTT
CTTAATTAATATGAAGATAATTTTCTTAAAAAAACAATATATGTATATATTGTCCATACATCACATCCTACCCTACCCTATTTTAAAACAAAAATG
ATGTGCAATTTTTTTTTTTTTTTTTTTCTATGAAAGGAGGTTGTTTTTCAATCATGTGAAAAATTACAAGTCATTTTTTGACTTAAAAAGTATAA
ATATACGATGCATTCAATGATGTTTTGTGTATTGTTTATACATTCAAGGAAGCTTATAAAATAGAACTACTCACTCATTTGCATTTGTTCATCCCA
TCACAATTACCACAAGTTATTCCAAAAATAAAGTGTGACGCTCTTGAGAGAAAAATAGAGAGAATACATCTTAGTATTAGCAATCCATCGAGA
GAGAAAACAAGAAAACAGAGGAAGAAAAAAATGGAACAGAATTTTTATTAATGTTCAAATTTGTTACAGCATACAAATGAGCTACTTAATTA
TAGTAGCTTGAGTTAGTCAATCACAACTAACCAACTCTATGATACCAACTAACTAATAACTAGCTAATACAAAGCTTAACTAACTTGATACTCC
CCTCAAGATGGGCAATATATATTCTTCAATCCCATCTTGTCTTTAATATACTTGAACTGCGTAGGGAATAGAGATTTAGTTAGTATATCTGCTAGT
TGCTCCTTGGAAGCCACATGAACAATCTTAATAATCCCTTCTTGGACCTTTCTCTCACCAAATGGCGGTCTATCTCTACGTGCTTTGTCCTTTC
ATGGAAAACCGGGTTTGTTGCAATATGCTGTGCTGATTTGTTATCACAATACAGTAATGTTGGTCCATTGTGATCAACCTTGAGTTCTTTTAGA
ATAGACAAAAGTCACACAATTTCACAAGTTGTATTTGCCATTGCCCTATACTCTGCCTTTGCTGAAGAACTTGAAATCGTGTGTTGTTTCTTAC
TTCTCCATGATATGATTGAATCCCCTATGAAAATAGAAAAACCTTTTGTTGATCTTTTTGTGTCTGGGCAAGCAGCCCAATCCAAGTCTGTGTA
GGCTCTTAGTTTTACTTCAGAATCAACAGCAAAATAAATTCCTCGTCCAGGAGTTGCTTTCACATGTTGAAGGACTCTAAGTGCTGCATTCAT
GTGAGTAGCACGAGGGACAAGAAGGAATTGACTCAGTGTATTTACTGAGTATGAAATGCCTAGCCTTGTTATGGTGAAACACTGAAGTTTTC
CCACGATTCTTCTGTATAAAGTTGGATCCGCCAACTTTTCCTTTTTATCTTATCCAAGCTTAAATTTGGCTTCCATAGGTGTGTTCATAGGTATAC
ATCCAAGGTGTCCCAAGTCCTCAAGAAGTTGTAAGGCATATAGTCTCTGTGATACAAAAAATGACTTTCTCTGATCTAGTTATTTCCAGCCCAA
AAAGAACTTAAGATTCCCTAATTTTCTTAGTTTGAATCGGGCATTTAACCCGATTTTCAATGCCTCCAACTGTTGTAGATCATTGCTAACAATAA
TGACATCATCAACATAGACCAACAAGAATATGAAGTGATTGGATGTATTGCTTTATAAACAATGAGTGATCAGTAGCTGAGTGATTAAAGCCCT
CATCAAACAAGGTAGTGGAAAACTTTGCAAACCACTGTCTCAAAGCTTATTTTAAACCATACAATGACTTCTTTAGCTTGCAAACAGGGTTGT
GTGGAAGCTCCCCCTTAGGTTTATAACCTTGAGGAAGAGTCATGTACACATATTCTTTGAGATCACCATGCAAAAATGCATTGTTTACATCCAA
CTTATGTACATACCAGCCTTTAATTGCAGCAAAAGCAATAACACGTTTGACAGTCACAAGCTTAGTAAATGGTGTAAAAGTATTAGCGTAGTC
AATCCCCTCCTGTTGGTTGTACCCTTTAGCAACCAACCGAGCCTTGAGTCTCTCAACACTCCCATTAGTATTCAGCTTGATTTTGTATACACAC
TTACAACCAATAACATGTTATCCTTCAGGTAAAGAAACCACAATCCAAGTATCATTGTTTTCATGAGCATCTATCTCAGTATCCATGGCATTATC
CCACTCAGGGATACCATGTGCTTGGCTAAAAAGTTTAGGCTCAAACAAACTCGAAATGGCCAAGACAGTAGCTCTGAAAGCAGGTGACAAC
CTCTGATAAGACACAACCTCAGACAAGGGATAAAGAGTAACAGATTTGCCCTGAAAAGATGGAGTAAAAGCATTAGCAACCAAGAAACAAT
GATAGTCATTTAAGTATGATGGCTTATGAGTTGATCTTCCTGTTCTAGTAATAACAAAAGGACCAGGGACTTGTTCAGACTCAATAACAGCAGC
AGGAACTTGGTCAGTAGGAGTTACTGGATTAGAAACCTGTTCAGGTAAAATAACATGGGCAGGATCCTGCGCTGGTGTAGAATTATGTGCAA
ATTCAGGTTCAGGATAGTTAGGGGTATAATGAGAAAGGACATGAACCTGGGAGAAAAAGTTATCATATGCAGTAGGAAGATGTTTAGAAGTA
ACAAAAGGAAAGATATGTTCATGAATTGAACATCTCTGGGATGGAAAAGCTTTCATTCCTGATGTTTGTGTTCAAGGAAGTAACTTGTAGGCT
TTCATTCCTGATGGATAACCAAGAAAAATGCAAGGAGCAGCTCTAGGGGCAAACTTAGATCTTGATTGACTCAAGGTAAATGCACAGGCAAG
ACGGCCAAATGCCTTTAAATGATTATATGGATATTGTTTGGAATGAACAATGGCACAAGGTGATTTACTTTTGAGATTACGTGTGGGTGTCCTAT
TGATTGAAACAAGAGATTGAAACAAGAGAGCCCTAGCAACACTGAGAAGATGTTGATGCTTCTTTTCAACCACAGAATTTTGCTGTGGTCTT
TGGACACATGAGTGATGATGCACAATCCCTAGTTTAGAAAACAATTCATTAAATCGCAGTTCCTTAGCATTATCAGATCTAAAGCCTTTAATAA
CTTTGCCAAACTGAGTTTGAACAAGTTGAATAAACTGGGAAAATGATAGATTGGGCATCAAATTTCTATTTGATGAGATTGATCCAGGTATACC
TAGAACAATCATCAACAATTGTGATGAAATATTTATAGCCCTCAGTTGTCAAAGTACTGTAGGGACCCCAGATGTCAACATGAATAAGATCAA
AACAATTCTTGGAAATGTTACGATTAGAAATGAATGGTAACTTCCTTTCTTTAGCATAGTGACAGATTGAGCAATGGAATAAAGAATTGGAATT
AGAATGAAAACTCAAATCTTTATTTAATGGGTGAATTTTTACACAAGAAGGATGTCCATCCATACCACAATGTGTGCAATTGTACTTGGATCGA

AAAGGTTGTACAACACCAACAAATTGTCCAGTAGAGTTGTTGGATGAATTTTCAGCATCACTAGACACACCTGATCCAACCAATCCAGATGTT
AGGTTCCTTTGTCTTTCTTCTTGATCGACTGAAGCATAGGCTTTGTTATTAGTGGGAAGAGGATCTCTCATTAGAATCTAAGATCTGACATTGC
TGAAAGAATTATTTAATCCAACTAGAAACTCAAGAAGTCGATCCTCTTCTTGATATTCAAGTATAACCTTCATAACACCACAACTAGAAACAG
GTTGTGGTTTATATTCACGAATCTTATCCCATGAGACTTCAATCAAGTGAAATAGGTAGTAACACTGCTTGATCCTTGTGTGAGATTCTGCATAA
TCTTTTTAGCTTCAAAAATTCTTGGAGCATTTTCTCATTAAACCTTTCATGTAGTTCAGACCAAATTTCAGCCGCATTATCGAGGTACATAATGC
TATCAGCAATGTCTCCAAAAATTGCATGAAGTATCCATTATATTACAGTAGTATTACACCTGCACCAAGCATCATAATGATCGTGTTCCTCATCT
GGCTGAGGAAACTTGTCATTAGCAAACTTTATTTGTTTCGCGCAACAAGAGCAATCATCATAGACCTTCTCCAAGTACTGTAATTCTCACTCTC
TGTCAGAATCTTAGGTACTAGTATCGCAACTGGATTATCACCATTGGAGAGGAAGAAAGGATTTGAAAGATCATTTGCAGTCTTAGATCGTGA
ATTTTCACCAAGAACAGAGTATTGATTCGATTCATTCGATTTGTTAACAGTATCGCGAGTGTTTCGAGTTTGAGCTCCACCACTGCGCGCCATA
GGAAGAAAACCAGAAAATCAACAGAACAAAGAGAAGAAAGATGATGAAGAAGACCACACAACAATAGTGGCTCTGATACTATATGAGCAA
TCCAACGCGAGAGAAAACAGAGGAAGAAGAAAATGGAACAAATTTTTTATTAATGTTCAAATATGTTACAGCATACAAATGAGCTACTTATAG
TAGCTTGAGTTAGTTACATCACAACTAACCAACTCTATGATACCAACTAACTAATAACTAACTAACACAAAGCTTTACTAACTTGATACTTAGT
TAGAGGAATTTTAGAGTGAGAAAATGAGAGTTCCTATAAAATTGTGAGAAATATTTTGTAGAGAGATAGTGAGATTACAACACTATTGAGTGT
GAGAGTTTTGAGTGTATTCGTCAAGTATTTGTCAATACTTGAGAGTGTAGTATTGTTCACCCATTTTATTGTTATAGCAAAAGGTTTACCGTACT
TTTTATTTGCCACGAGATAATCTTTGGGAAGCATTTAGTGCTTTGATTATTATTTATAAAGGACTATTTGCATTGGAAGATATCCAACAATTCTAA
ATATTCTCTGTGATGGCAGCTTTGACTAATGTTTCACATCAAAAAAGAATTTTGGAGTCCCCATTGTATTTTCCTGCAAATATTTATCTTGAAGG
GGAATTGAAATATTTTCCATTATTCCCAACAACTTGTGTCAGATTATCAAGGTTGGAAGCTCTACTTCTGAAGTAGCGTTGGTTATACGCACAA
CGTAAGTAGAAATGTATGGGAAACTGGAATTTAAGTAGTCCACTTGTGACTCTCTATGTCTTTCTTAGGAACTCACTTATAAGAGTGATTCCGT
CTAGAAGCCTGTCCAAACCTATGCTTTGTTGATAGGATTATGATATATATATATATATATATATATATATATATACATGGTCTTTCCTAGGAATCACCTA
GCCAGTACAAAGTTTTGTGAGACTCAAGAAACATGACGGGCAAAAAATTGGATATTGAGAAGTTTAGTGACAACGGTAATTTTTCCCTCTTAT
AAAGTAGGATGAAGGATCTCCTTATTCAGCAAAAGGTCTACAAGGCATTATCCAGCCAACAGAAAAGCCTAGTACGATGAAGGTTAAGATTG
GGAGGAGATAGATGAGATCGCATGTAGTACAATTCGTCTAAATCTATCAGATATCATCTTGCGAAATATAGATTCGAAAGAGTGCAAGATTGCT
AAAGAAATCTAAGAGACTTTTTAGAGAATTTATACATGTCCAAGAATCTTTCTAACAAGTTCTACTTGTGGAAGGAACTGTACTAGATGTGGG
AGCAAGAGAATGTGGATATGCTCCATCATTTGAGTAATTTCAATGGCATGATATTCTCAAATGATAAGGTTCAAAGGGAAGTTTAGGGATGAG
GAAAAAAATTGTGTTGATAATAGAAACACTTCCTCAATTTTATGATCACCTTATCACCACTCTTACTAATTGGAAGGACAAGTTGGAGTATAAA
ATCATTATAGTGGCTTTGGTTTATTTGAAAAGTAAAAGAATGAGGGAGGTGTGTAATCTCAAGCCCTTATTTTAAAAAATATGGGGAGAAGTC
AAACAAAGAAGGTAGGAAAGGAAATGATAATTAATTCCATAGGCAGATCAAATTCCAGAACTAGAATTGAGGGTTGTATTCAATGTGACAAG
TTGGATCATATTAAGAAAATTCATAGGATTTTGAAGTAAAAAATTCAAGAGGCCAGCAAAGAAGAAAAAATAATGGTGACACTGACAGCAACA
CAGCTACGAATGCCACTATAGAAGAAGATATGATGAGAGTGATTGTTAATGAATGTTTGGAGATGATATCACTGAGTAGGTGGTAGATTCAGG
AGCTTCTTTTCACATCACTTTCAATTAACAGATGTGTTACCACATACAATGCTGGTGACTTTGAAAATGTGAAGATGGGTAATTTTGCATGCCC
CAAAATTATTCAAATGAATGTAAACTATCATTTAACATTGAAGAATGAGAGGTATGTTTTGTATTTTCATCTTAATCTTATCTCTGTACATGCCTT
AGATGAGCATGGGTACGAAAGCTACTTTGAAAACAAGAATTGGAAAATTTGTAAAGTGTCAATGATTGTTGCAAGAGAAAAGCTATATTGTT
TGTTGTATGGGAAAGCTTGGAACAACAGTATAAATGTAGCAGACGGTACTTCACAAAATTTATGGCACAACGACTTGACCATATAAGTGAAA
GGGTTTGTAACTACTCGCAAAGAAGTTCCTTTTATTGGAGAAAGGTACTCTATATGATCCTTGTGAGCATTGCTTGAAGCAACATAGGGTCTTA
TTTAAGAGGTCCTCTACTAAGAAAAATGAGGTACTGGATCAAGTTTACTCTAACGTCTGCGGTCCCATTAAAGTTAAATCCTTGAAATGAGCA
AAGTATTTTTTAACATTTATAGATGATGCATCAAGAAAGGTTTGTGTCTATATGCTAAAGATGAAAGACCAAGGGCTTGGATTTTTCAACAATT
TCATGATATGGTAGAAAGAGAAATTGGAAAGCTATTAAAGTGTCTTAGAACAAACAATGAAAGTAAATACACTTCTCATGAGTTTAGAAATTA
TTACTCTAAACATGGGATTCAATATGAAAAGACGGTACCTATAACTCCTCAATATAATGGTGTAGCAGAATGAATGAATCATACCATTATAGAAA
GGATCAGATGCTTGATGAGATCATTCAAAGTACCAAGACCATTTTGGGGAGCAGCAATACAGGCTGAATGTTATCTTTTTAATAGATCTCTATT
AATTCCATTAAAGTTTTATATTCTAGAAAGAGTATGGACAAAGAAATATGTGTCATACTCACACTTGAAGGTGTTTAGATGCAAGGCATTCATG
CATGTACCAAATGAACAAAGGTTCAAACTTGACGATAAAGCAGTTCCATGTATCTTCATAATGATTTAGGTATAGGCTTTGGGATCTAGTTAAC
AAGAAGGTCATAAGAAGCAAAGATATGGTCTTTTATGAAGATTAAATAATTGAAGGCGCGAGAGAAGAAAAGAGTCAACCAGAAAAGGTTG
ACACTAAGTTATTTCCTCCAAAACAATAATGGTCAAACTATGAATTACAAGAAGTTGCTGAGATGTCCCATAGTGATGATAGTGATGCTGAAG
AAGATGTACCAACACAAGAAGACATCAATCAGGGGGAGCAATGAGTACCAGCAACCAGAAAATGAGGGATCTCACAGTTGGTTAACTAGAA
GACTAATTAAATACAAAAGATATCTATAGTTGGAGTACATTCTTCTAACTGATAAGGGGCAGCCAGAGAATTACCAAAATGTAGTGGCTCATAA
ATACAAATACAAGTGGACAGGTTCCATGAAGGAGGAGATGAATTCCCTTACGATGAACAACACCTATAAGTTGGTGAAACTTCCAAAAGGCA
AGAAGGCCCT[G/A]AGGAATAAATTGTGTTTAGATTAAATGATAATGGACATGGAAACTTGGTAAAGTACAAAGACTGATTAGTTGCCAAGGG
TTTTGGTAAAATAAGAGGGATTGATTTTGATGAAATACTTTTGACAGTAGTGAAAATATCATTAATCAGAATAATCTTTGGACTAGCAGTGAGT
CTAGATCTTGACTTTTAACAGATGGATGTAAAGACTAAATTTATTCATGGAGATTTAGAAGAACAAATTTACATGCAACAATTTGAAGGTTTTG
AGGTGAAAGAGAAAGAACATCTTTTTTGTAGAGTGTGTAAGAGTTTCTACGGTCTAAGACAGGCTCCACGATAATTGTAAAGAAGTTTGATT
CATTCATGATGGTATTGGAGCTATAAGAGATTAGCGGCAGATTCATATGTCTTCATAAAGAAATTTCCTAATGAGGAATTTTTTGTCTTATTAATA
TATGTGAATGACATGTTAATAATTTCCCAAGATGGTAAGATGATAAGTTAGCTGAAGAAAGAACTATCAGGGGTCTTCGATATGAAAGACTTG
GGGGCAGCCAAACAAATCCTAGGAATTAAAATAATTCTTAATAGGCCTTCTAAAAAATTATGCTTTTCACAGGAGAACTATGTTGAGTGAATA
CTTGAGAGGTTCAACATGAAAGGTGCGAACCTAGTTTCTTCACAACTTGGTGGCCATTTCAAACTCACCAAAAGTCAGGTCCTTCCTTTGAA
GAAGAAAAGAGGAAATTGTTGGTTATTCCTTACTCTTCACCGATTGGAAGCCTGATGTACACAATGGTTTGTATGAGATCGAATATTACTTATG
GGAGTCATAAGTTATTTTCTTGCAAATCCAGGCAAACTGCTTTGGGAAACTATAAAATAAATTTTCAAATATCTCAGAGGGACATAAAAAATAT
TTTTGACCTTCGAGAAAGGAGAACAAATCTTGGAAGGTTATGCAGATGCACATATGGCCAATGATCTTGACGGTAAAAAGTATACATCTTGAT
ATATGTTCACTTTTTTAGGGGGAGTTTGATCATGGAAGTCAAAGTTGCAAAAGTGTATTTGCTTTATCTTCCACAGAAGGAGAGTATATTGCTC
TAGAAGAATTTGGAAAAGAAATGTTATGGCTAAAGAATTTTCTCCAAGAATTGGGTTTGAAAGAAGAAAGGTATGTGATTCATTGCGACGGC
CAGAGTGCAATTGACCTAAGCAAGAATGCTACTTACCTCTATAGAACGAACCACATTAAGTCAGATATCATTGGACTTATGATGCACTTGAAA
AGAGGCTATTCCAAGTGCAAGAAAATCCTCGACATAAGCTTGATTTGTCTTTGGATGATTTCTCGATGATGAAGACACGACCAATTCTTCCAC
ATAGATTGAAAAGGTGAGAGAATTGATGGGTAATGGTTATTTTTTAACCACGGGAAACTTAAAATTGTAATTTTTGTACTTAAGGTATAGATAA
ATTTTGAACCATGCATTTAATGCAAGGTTGATTTTTTGTATATTGCTCATACATCCAAGGAGGCCTATAAAAAGAGCTCAATCCTTTGCATTTTT
TTATCCCATCACAAGCTATTCCAAAAGTGAAGTGTGAGACTCTTGAGAGAATAATAGAGAGAGTACCTCTTAGTGAGCGAAATCTGACCGAG

AGAGAAAAAGAGAGTTCCTTAAAATTGTGAGAAATATTTTGTAGAGAGATAGTGAAATTGCCACACTAGTCATGAGTGTGAGATTTTTGAGT
GTATTCCACAAGTATTTATCAAAACTTGAGAGTATAATATTGTGTACCCATTATTGTTATTATAGTTGAAGGTTCACCTTGATTTTTATTTGCCAAT
AGATAATTTCTTGAGGGCATTTACTGCTTTGATTATTATTCATAAAATGCTATTTACATTAGAAGAAATCTAAATTTTTTAAATATGTTGTGGGATT
GTAGTTCTGACTCATCATTTATATCAAAAAAAAATTTTAGAGACTCCATTATATTTTCTTAGAAATATTTTTCCTGAAGGGAAACTTGAATTTTATC
CATTATTCCCAACAATAGACACACACTGTGTCCATATTTCAAATAATTTATGACAATATACTAGTAGAGTCCCTACTTAAACATAGTCCTATAATT
CTATTTAGGAATATTCTCGGGGAGGATTTGGAGCATTTGGGACTGAATGAAATTCAACAGCTTGAGCAAAAATTGGACATGTCATTGAAGAAA
ATAAGATCAACTAAGGTACATACATACAATTATTATTGAAAAATTAAATAGTTTATTTTGTTGCCATAATATTGGGTCAAAAAATATTTACTAAAA
TTCTTTTGTTTAATTCATATGTACAATAAATTAATTGTTAATCATAATAAAATGGATAATTTACATACAGTTGAACATTTGTTGCATTTTATTCTGA
AAATTAACGTGCAAAATTCTTTATATAAGTCTACTGTTATTTTTATATCACTATTGAATTTAATTGTACCGCTAGTCATTGGCATTAATTTTGTAAT
TGAAGTATTTATTTTATACTTTAAAACGATCGAAGGAATTTTAACATTGGACAAAGAAAAAGATCTTTTATTTTTTAATTATTTTTGAAGTAGGG
CCATGGATGTTGTGAAAATAGAAATAATAATTAATAAACTATATTATATTTTTTTAATAAAAAATTATTATTATTAATGTGAGATTTATCATTATTTT
GTTATTTAGTTTATTATTTTAAAACAATTTAAAAATATATATTATTTTATTAATTTTATTGTAATTCTAAATCTCTATTATGCACAATTTATTATTTATC
TATTTATTTTCAATATTTTAAAACTTACTCCAATGAAACTAAGAGTTACTTTATTTGTGTTAAAAAATAATACAATCACAACATACCCTTCAAGT
GATCAATTTAAAATGTAGAAAACAAGTGTTTTTTCATCATTGCTTACAAAAAAATTTGGAAACCGCCCAAACCGAACCGAAAAAAACTGATAA
AAATTACAAACCGAAATAACCCGAAAAAATTACAACCCGCCCAATCAGGCGGGTTGAAAATAGGTCCAACCCGCCCAATTAAACCGAATAAC
CCGACCAACCTGACTTTGGCTTTTATTTATTTTTTTTTTTAACTTTTTAGTTTTTATTATATATAACATAAATGATTAAATATATAATAATATAAAAA
TATATACTTATCTGTTAGTTTCAAAGTCATATATATTGTGTTGTGCTTTGGTAGAATTTGATATTTTTAATTTATCTTCTTAATTTTTATTGACTTTG
AAACCAAAAAAAAAAAAAAGTTTGGCCGGTTTACAATTTTTTTTTTTAAAATTTGGCCGGTTGCACTTAAATTTTAGCAACCCGAACTTTAGA
TTGAGTTAGAAAATATATAGGATAAACCGCCCAAACCGACCGATGTACAACCCTACTAATTATACTGATTAGTCAATTCTTTTCAAAAAATTATT
TTCGTTAACAATGTTTAGTTTAAAGTAAGGAGATTGGCTTACATTATATATGAGAAGTAACTTTGCAGTTGCTTTTTAATTTATTCAAATAAAATT
TTAATTAAATAATTTAAAATTTTGATGTTATTCTTTGTTATGATTTTTAAGTAATTTATAAGTTTCTTATATATATATATTCTTTTTATTGAATTTTTTA
GAAAATTAATTATTAAATATAAAAGTTAAAAAAAATATAATTCAGAGAGAAATAAATGAAGGGAACACTTAGAGTTTTTACGCAGTTGGGGCA
TTAACGAGCTGTAGTCCAAGTGTCAATATAATTAATTATTTTGCTAGTAAAGCTTAAGGGATGACAGTAAGTATGTGCATTCTACTCTTTGCCTG
AGCAAGTATACCAAGAATTTCTAACCCTTTGCATGAAGGATTTGTTTAGTATTTATAGTGTACATGTGAAGGTCCTGCTAGTTACCCTTTTTCCT
CCAATGGGGAGCTTACAAATAATATATTCACCTTTTTTTTTTGAAAGATTGTTTAAATAATTCACTTTGGGCTTATCTGGTGAAGCCCGACACAT
AACACACCTTGTGTATGCTGACGTGGAGGCCTTATCATGTAGTTAGTTATTCATACCACATCCTATTCATGGGTAATCAATGACACATTAATTAC
CTGATTTTAAATTATCCAGTTTCTCTTTTTAGTAAGATCTTATTCTATATCTAAGATTTTGGGAAAATTATGCTTCCGAATTTGTACAGAGGACCT
CTTCTAAATAAACAAAGACATACCATTTCAGATGCTGACTCAGCTTGACGAAACCTTGCCTGAGTGAGTAGTATTGATGTTTTCTAAGAAGGC
AGAGTAGATCTTGGGCTTGGCCCAATTTATGTTATTTGGGTCCACTGATGGGCCTTAAGCTTATGTTTAAAATCCACATGTCCCATGCTGAAAA
ACACGGATAACAAATTGAAACAGTTATAATCCACGGATATGAGTGAGTACAATGTATTATTGACCTAAAAATCCTTTTCGCAAGATTTTTTTTG
TCATGATGAGAAACAAAAGTTGAACATTGAAAAGATCTAATCTAATACTCTAGAAGGAAGGGTAAAGTCTCGCCACACGTGGAGCACAGTAA
AAAAGGAACCCCATTTTCACACCTTGGGTGCATCACAAGAAATATAGGAGTCATGCCCCCCGTGTCGCACCATTAGATCACTAAGGAAAATAT
GAGGGGCCTAGCTTTCATGCATGGCATCACACAACATTGGTCATGAAGCGAGATGAAGCTCCCTTCTGCATGGTGTTGTGTCGCACGAACCA
CCAGTGAGATGGGGGCCTGACAATGTCTCACTCCCTCACACCATCCTAATCCCCTGCATCGTGTCATGCCACACCAGCCTTATGTAAGACAAG
GATCTCACCTCTTACTGATGTGATGCATGAGTACTATTTGGAGGAGACTATATACAACTCGTCTTTCTCACCTTAGGCCCACTCCCCTCAATGTA
GTTTGTCAAGAGGTTCTATGATGCCAAAGTCCGTAAGGCATACCCACTAGCAGGTGTGACACCTATAACTCCAGTCATCGGGGAAGAAAACT
AAGCCCAGGTGATGGAAGTACATATAGACACGAAATCTGAGGCTAGTGTCTTGCTAACAACCCTCACAAAAAACAATTGAAGGCCACAATGT
TGTCCATCTTTGATATGGTACCCCCTACTATGAGTCCCACAAGATCCAAGTACATCGCATCACTGACCCGACAAAGAAAAATGGTTTTACTCA
GTATTGTGGAGGCAATGAATTACCTAAGGTGGAAAAACCAAAGGTTGGAAGAAACAATTGCAGACATGCAAGAAGTTCTGAATGACCTTTTA
CACGGATATTGAGATGCTCCCTTGTACAAGCGTAATGCCAAATGAGCCGAGAACCTTAAGGTTAGGCTAGAGTAATATCTAAATCGTCCACAA
TAACATGCCTTAATCTTGTCCACGAAGTGGTGCGGCCATGCAATGCCAACTTTAGACCTACTGGAGGCGAAATGTCTCAGTCCAACCCCAGA
GGACCTTGGCGAAGGCCACTCCCCAATATTTGAGGGACAAACTAAATGGAAAATCATAAAGGCATACGTCACCCATTGCGCCTTAAAAAAGT
GGAGATAGGAAAAAGTCCACACCTCAAGGCCTCCAAAATAAACAAAACAGGAAAAGAAAGAAGTTAAACAAAAAATGGAGGATTTATGTA
AGAAAGTTTCCTTAGTTGCATCGGAGGAATGTAGCTGGGAAGACAACGAATTAAATGAGGAATCACCATTAACAAGAGAGATCCAGGCTGG
ACCACTCCTTCTCAACTTCAAAGAGCCAAACTTCTCATCATATGATGGAACGACAGATCAAAAATACCACTTAGATGCCTTTAACGAACTGAT
GACTATGAAAAGGGTCTCAAGAAAAGCAAGGTGCTAATGTTTGACAATGGAAGGTGCAACATGCCAGTGGTTCAAATGCCTGACTCTCAGC
TTTGAATCATTATGGAAGCAACTCACTAATTGCTAGCCTAGCACTATGCCTCGTCAGACTATACCATCCAGCACAAGTATAGCTATGTCAAACA
AGGAGAGAATGAAAGCTTGAAGAACTACATCAAGTGCTTCAACACAGATGCCATAAAAGTAGGGCGACTGACCAAGGATGAACAGATAATT
GCCATAGTGGATGGTGTATACCCCAAGGGAAAGATGTGGAACAATATGCTTAAAAGAGAGCTAAATGACCTCAAGGATTTTAATGAAAGGGC
AAAGAAGTAAGGCTTTGAGAGGATAAGAGATGACATGAATAAGAGGCCAAAGAGAGAGGAAGGTTTATGTCATCTATGATACACCCACTTTA
TGAAATTGGCTGATTCAAGGGAACACATCTTCTTGACACTTGACAAATGACACTAATTTCCCATCAAAACACCCATAAGGATTGATAGATTTA
GGAGGGATGCCAACAAATTCTATAGATTCCATAAGCACTTTAGACAAAAAACTAATGAATGTTCCCACCTCAAGATTGAAATGGAGGAGCTC
ATATGAAGAGGATACTTAGATTAATATCGTCGAGAGAACCAGTGAAATGAAGAACAAGAGCAAGGGCAGTGGCAACAACATCGTGAAGAAG
CACCACAGGTGCAAGGAGAAGTAAGAATGATATTTTGGGTGGCCTAGGCTAGGAGGAGACTCCTAAGAGAGTAAGGATAGGTACGCTATTGA
GGCAAAACAAGCTCAATACACCATGAGCATAGACAAAAGGCACCAAAATTTTTTAAGGTAGCAGATGCCCCATCACATTCAATGAAGAAGAT
GCCATTGGTGTCCATTTCCCACACAATGAACCACTAGTATATATTGTACAATTGGAAAACATGAGATGTGACAAATTATTTATAGCTCAATACAT
CATGAGCCTAGACAAAAGGCACTAAAAAATTTTAAGGTAGAAGATGCTCCGTCACATTCAATGAAGAAGATACCATTGGTGTCCATTTCCCAC
ACAATGAACCACTAGTATATATAGTGCAATTGGAAAACATGAGATCCCAAAAAGTATTTATAGTGTAACGTCCCCACTTCAAGCCTCAATTGGG
CCTTCCCCACGAATTTATGGCTTTTATACACGAGTATGTCTTGCTGACCGCTTCCTAGTCTGATTACTAGCCCTATAGACCAACACAAGTGTTTC
AAGCATACTTTGTCCTCACTCGCACGCTTCTTAGAAAAACTTCTTAGGAGGTCACTAATCGTGAAATTACCACAAGTCAAGCATGCTTAACTA
TGGAGTTCTTTCATGATGGGCTACCAAAAAACAACATGCACCTTATTAATATAGGTAGTACAAATAAATCCATTTAATTCCATTGTAACTGTGTA
GTCCCATGCCTACACAATCTCATAATCATAACACTTGACCTTCCTTAGGTGATGTGTGATTGCACAGCTTACCTAGTATCTTCCCTTACAAATTA
CGAGACTACTGACAGCCACAATCAACACCATTATAGGGTCTAACATCCTTGTCGACAATTTGGCTGGGTCAAGGCTTTAATACTTTTTGTAAC
GTCCCCACTTTAAGCCTCCAATTGGCCTTCCCCCACAGATTATGGCACTTAGACACGAGTACGGCACTCTGGCTGCTTCTTGGACTGATGACT

AGCCCTACAAACCAACACGAGTATTTCTAGCGTGGTTTTTTCTCACTCACACGCTTCTTGGAAAAACTTCCCATGAGGTCACCCATCTTGAAA
TTACGCTAGTTTAAGCACGCTTAACTGAAAAATTCTTTCGTGATGGGCTACCAGAAAATAAGATGCACCTCAGTAATATAGATAGTACCAATTA
ATCCATTTAATCCCTCTTCAGGTGTACTCCCATACCTACATAGTCTCATAGTCATAACACTTGACTTTCCTCAAGCGATGTGGGTAATTTGTGTA
ATCCCACATTGCCTGGGAAAAGTTAAGTATGAAAAGACTTGAAGACAAGATAGACTACTAGTTGTCAGAGATAGACTATGGCAGCTCTGTTG
ACCTATTGTATAAAAAGACCCTTGAGAAGATGGGGCTAGGCCTGCAACACTTGAAATCAAGAAAAACCCCATTTACGGCTTCACGAGAGACT
CTATCCTTCTGTTGGGCATGATAGGACTAGCATTCACAATGGGATAGAAACCGCATAAAACAACCATAATGGCAATTTTAATTGTGGTAGATTT
TTCTGTTGCTTTCAATGTCATACTGGGCATACCTTCCCTGTATCGATTCAATGCTGTGACCTCAGCTTATCACCTAGCCATGGAATTCCTAACAA
CTGAAGGATTAACCATGATGAATGGAAAGCAGAAAAGGCAAGAGAATGCTACAACACTTCTTTGAACATTTCCATAAAACTTAGGGTGGACA
TGACTATGATAATGAGTGAAGAAAACCGACAATGAAGATGCATGACCAACCTACAAAAAATGTGAAAAAAGCACCCATTAGCAAAAATCTTA
GAGAAAAGGCAGTGAAACCATTTACTCAAATGTCGTCAAGTCTCCATACATGAAAGAGGTGAAGTACCAATGCAATTTTAGCCTTGTGGCTT
GATCCGTCTTTACATAAGTTATTAATATATAAAGAATCAAGGACTGTACTAATGACAATTTCCAATAGTAGTAGTAAATATTTCAAATTTCTATTT
CATTCAAATGAGTAACACCTATCAAGAAGGGGTATCCTCGATGAGCTTCAACCCATGCATACAGAGTTACTCAGAGCAGTTATCAATGGTATG
TTAGTATATATGTATTACATCTACATCTCCTACTTGACTATCATCCTCACATTTCTATTTTTCAAACGAAATCAGTAGTCATAGTATTGAAGAATGT
CTTAATTGTCATAGTATTGAAGAATGTCTTAAGTGTCCATCACAATAAATAGCGCTACACATTTGGGAGAAAGAGGAGGTAAGAATAAAAAA
AAATCTTGTACACGCAAGGACTAGCTACCCTTAGTCACGAAAAGTATAAAAGATGCTTTAAGAAATTCCTTACCCCCAATACAGATAAAAAAC
TAGGCCTGAGGCGCCTAAAAATATGAGGGCCTTGAAAGACGCTCAAGAAAAATAGTACCATAAAATGAGGACACTTCTCGAAAAATTCTTAA
AAGCAGGCACAAAACTATCAATAAACATAAGAGGTTGAGATAGATTCATGGAACTAACACTCCATAATTAAACAAGCAACAAGAGAGACACA
AGAACTAGAAAGCCCATAGTCCTCATTACAAAAAATGACAAACCCAAAGAGAGAGATCTGGCCAAAAGAGCCCATACTCGATCATCGAAAG
AGTTGCACAAGATTGATGACCCTCGTAAAGGAAAAGAGTCATAAAAAAAAAAAAGAAACATAGCAAAGTCCAGTACAACAATTTTAGAGTTG
ACACAAACAGCCTCGGAGCACAAGTGGCCAACAAGGAAGAAAAAATATAGATAGCCCCAAAGGATGACAACGTGTAAAGAAAGGAGTCTT
TTTCTCTAAAAAAATAAATACTTTAGTCACCCAATGAGAATAATAAAAGATAGCTCAAGCCCAACGACCTCATGGAAAACAACACGAACAAC
TTCACAAGGGGATGGAGGGCCAAAAGTGGATCATCTATGAGACACCCTTTCAACAGCAAAAGGATAGGTTGGGGTAGCACTTTTAAATATTT
TATAAGGTTTTGTCGAAGGTCAAAGCCTCCTAAATGCGAGCGCTCTCCTCTTGACCATGAAAGTATTCATCTAAGTCATAAACCAAGTAATCTA
AATGCTCTACTTCCTTGTCAAGCATCTTTTTAGATCTTTACACTATTTTGATAGGATGGAGGGTGTCGTTTCTCTCTCGGATAGCTGACTCAGCA
ACCTTTAAGGCCTCGGCAAGTGCACTTTCAGACCAATTCTTGACTTCACACAAAAAGGTCACCTTATTTTCTACTAACTCCTTCTTTTTCTCAA
CAATAAGAAACTTGGCCGAAAGCTTTTCAACACAAGAAGAATGGCATTGTACACTCAAGAAAGCCTATTCCAAAACCGTGTGCTTTTTTCCC
TCATATCAGTCTTGTCACTCTAAGGATATTAGGACCCTAGTGGAGGATATGAAGAGTTGGCTTGGAGACCCTTAAGGTGGTATGAGCCATGATT
GAGGCTTGCAAATAATATAGCAAAAGATAGAATAAGTTTTGTATAAAAATATAAAGAACAAACATAAGAAACTCTACGGAGAAGAAAAAGGT
TGATAGCATGCCATAAAAAAAAAATTCTATCCCAACTGACTATTTGGACTAATGAAGTACATACATCCACATCAGTACAACCTTGGAGCCATTTC
CAATTATCTTTTTCATAGTCGGACATAGTGTTACTAATGTCTTGGGAGGATTGAGGAAATAGAGTATGCGCAAAGGAAGGTTCCCCTTGGGCA
AGATATGGAGTAGATCTTGAAGAGTATCTCGAAGGAAGGGAAGCATAGGTTGCTACCGACACTTGAAAAAAATCTTGAGGATCATTAATCAC
TATCAGATCTGGGGGCTCCAGACTGATCTCCTTAGGAGGACTATTGTACATGTTAGAGCTCTAGTGACCTTGATATTGTAGAGACCTAAAGTGT
TAAGGAGAAATTACTTCCACATTTTGAGCCCACAAAATATCATCACCCAAAAACTCAGAAAGTGGCCAGGGCATGACATGAAAAGCAGAACT
TGAGGATCTTCACTAAACATTATCCAAAACCTCAAGGGAGAAGCCATCATCAGAGTACTAGAAGAAGCACCTAACGACTCCCCCTTTGTCAG
CTGAGGCATGGGGGGAGGGATTTCTTCAACAAATTAGATGCCTTGGTTACAACTCTTTCAAAGTTGAATGACGAGGAGATCCAACCTCATCCT
TAGTAGAACAAGTTTTTGATAAAGAGGCAAAATCTGCCTTAGTTGCGTGAACTGCTCTTCAACTATTCACTTCTTTGAAAGAGGCAGTTGGGA
TCACGACCTCAACGTCAGTGTTATCAAAACCATCGTCGTAATAGTCAACGGGAGAAGGATCTTCTCCTGCCTCTTTGGATAGTAGAGTTTTGT
GTATGTTTTTCAAAGTGATCTTGCTCTTTCAATCAGTGAGGCCATACTTCTTCAAATTGGAGGGTGTCAAAAGGTTGGCACACATCTGATCCTC
TAGAGGCACGCCAAAGAGCTTCTGTGGCCCCTTTGCGAAGAATTTTGTTGACAACTTTGATATGTCTCTAACCTCTTAAAGGAAACTTTCAGA
CAATCATGTCAAGAGCAAAGTATAATATAGAAAATGAAGATCAATGAAAAAAACTTACTCGCGATCATGAAAAACTCCTGCTCCACTTAGGCA
CACCATCAACAAAGAAGAAGTATGTCCCCCAATGCCTTGTGTTAGATACACACCTAATAACTGTGGGCATTTTTGGAGAATCCTTTTGAAGAA
AGAAAGTACCTCATTAACCTTTGTTAATGGAAAGGGGATACACATAGTGGACCTCTTTTGGCATAGGCTCACGATTAGGTATACCCTTGTACGC
CAAGTAAAGGCAAGTAGATGCTGACCAACTATTTAGGTGGAGTTGAAGAGAAGCAATACAAATCTAGTTGATCATGTTCGTAAAGAACCTAT
GGAGAGGTAGGTTAATCCCTGCCTTCAAGACATCATTGTTAAAGGTGAATGTTCCCTCAAGGGGAAGAGAAGCCCACCAAATAGGCTTTGTA
GGCATAATCACTACATGCACTCAATTGTAGTAGATGCTTTTGAGTTTCTTGATTCGCTTAGGGGACAACTCACTAAGAACATTGTCCTACAAAA
AATTGGTGTTAAGATGCATCGCGGGGAGTGCAGCATGCCTCATGTTTTTCCTAAAGGACGTATCCACACAACATTTCAAGTTTTTCATCCTATG
TGCAGTAGTCTTGTGAGATGAATTGAGAATCACAAGACCAATATTTTTAGAAGGTTGCCTGGGGATTGAAATAGGCCCTTAAAGGATCCCTCA
AAAGACCCTCACCAAAGGGTAGAAGAATGAAGGAAAACCAATCTCGAAGCTAGGATGGCCCCTCCCAGGTGAGCTTTTGTGAGAAGGCAG
TTACGCTGGACGGGTGATGCGAACATGGGGCATAAACTTCTCCAAGGTCTCCAGTCTCACCTTAAGGACCAAATATGAATATCATAGTTGGTC
TAAGTGCATTAAGGTATCAATAAATATAAGATGACAACCCAATTCAATGGAGATTATGTTAGCTCGAGTTCTCTCAAGTAGTTCCTTTTGCTCAC
ATAATAACTCTAAGTGAGTTATCAGAGCCTCCCTCAATCTATGGTAGATAATGAAGCGATCCTCAATCCAATCATCTGAGTCAAATGACAACAT
ATCCATTGGGACAAGTCAATTAGGAATTGAGCTACTAGCCTCAAAAGACATGGGAAAGAAGCATAGTAGTAACACTAAAAGCCTACACGTAA
CAAGTAGAGTGGGAGTCAAGGAAGTTTCAGAATTAAGTATACGAATAGGTAGAGTTACCTAAATCCTACTTTTCTCTCATCCACTTGCTACCTA
AGAGAATGAGGCCTAAAGGAAAGGTTACCATGAAGGGCCATCACTAGAAGGGTAAACCAATAAGTGACCCATATGAGGAGCGAGGGTTAAC
ATGGGAAGAAATTCGGTCCCCAAAACCCATGCACATGAATGCATGCAAAAAAAACTTCTTGCCAAAGATGATCTGGTTGGAATCAAACTCGA
GACTTTAGCTCCTTTGCCAAAGACTTAAAGTTAAATCTTCTACCATTAAGCTAAAGTAAAATGTGTGAATGAAACACTCGATACCACAAAGTG
GTATTTATAGTAACAAGGAAGAAGATTCTCAATCCTTTAATCAAAGGGCAATCAATAGTGGAGATTAACACACTTGCTTCCATCAAGATCCATG
ATGTCAATGAATGGTCCATACTCTCCCAAAAAGATACTGCTAGGATCATGGATGATGTGTTTCTTGAAAGGTTGAAGCATGGAGTAGTAAAAC
ATAAAGAGTACAAGAGTACACAAGAACACTTGAAAAGTGCACTCATAAACTCATGGGCAAAAGTTGACCCACAAATTCTTTGAGTAAGGATT
TCAAATTCATGAAGAGAAATCAAAGTTAAGCAGTGAATAGATATAATCTTACTCTTTGGGGAAGGGTCATAAAGGGACCCTATTTTTCTCACAT
TGGGTGCATCATGGGAAATATAAGGGTCATACCCCCCTTTTGTCGTGCCATTAGATAACCAAGGAAAATGAGGGGCCTCGCTTTTGTGCATGG
CACCGCGCCACATGGGTCACAAAAGCAAGACAGGGCTTGTTTCCGTGCATGGTATATCTTGCTCCCCTACATTATTCAACACCACATAGACCA
TTTGTGCGATAAGGATCTCACCTCTTAGTGAGAAGATGCATGACCCTTTAGGGGAGATCCCATGCAAGTTGTCTTTCTCACCTCAACCCCACT
CCCCTCAAAGTGGACTTTCAAGAGGCTCTATGAAGCCAAAGTTTGTAAGGTGTAGGTCCCAAAAGTGAGTCTCGACATTCGGTTGGATGAGA

GGCACCATGAGGATAGGTCTGCAAGCACACCCATGACGCAAACAAAAGATGAGACGAAAGAGTGAAGGTACAAGGATGTACCTACGATATA
AGGTAGTGGGAGCATGATCCCAGGTGTCAGTATAACCTGACACCCAACCACCACTACTTTAATTCTAATGCTCGAACATTTTTTCCAAAGCCA
TTTGCTAGATAGTACATGGTCCCTTTAGGGGGCCACTTTGAGCATCTCAAATGCAACAAATAAAAATTAGAGCCCCCTATAAATACCTCTTCAC
TGCACTTGAGAAGGAGTTGGTACCTTGTGTGTGGAAGAGCTGTGATTTGAATACAACATTCATTTAATACTTTTCATACTTACTCTCACTTAAT
CTCAAATCAGCCTCTACTACTTACTCTCATTTTCTCATACTTATCTAAGTCAATTATAACTCATTATTTCTTACTATAACATTGTTGACGAGTTCTT
GCTGTTAATACATAAAATGTAATTTCCTTATAAAAAATTTATGATACATTATAAAATTTGAAATATTTGCTTAATATGGTAGCTCATTATATTACATG
ATAATTTATTAAATTTTAAAGTAAACGTGAAGGTTACTAATGTATAACATAATAACTAATTTGTATATCAACATGTTTATAAATAAATTTTAAAAGT
AACTAACTGTTATTACAAACAATATGATATAAAAATAAAAGAAGAATATACATACAAAATAGATGAAAAATAGCAAGGACAAAGCCCCCTCTA
AAAGTACAATCACTTGAAACACAAGATATATTTGGAAAAGTATGATCAACCTTATTTGTAGATTTTTTAACAAAAATATAAAATAAAATTATTTA
AAGAAGATAATATATCTTTGATATCTTTAATAAATAGACAATAAGGTAAAATTATAGTAATATCACTATAAGTAGCATGAAACTGATTAGCACAAT
CAGTCTCCACAACCACGTAATTTCACCTCTTGGTTTTAATCCAACTAAGTGCTTCTTTCAATCACATTATTTCAGCCATATCAGGTGAAACCTTA
CCCTCCTAACTACCATACTTAGCTTCAATTAATCATCTACTAGAGTCACGGGCAAGGAAATCGTAACTAAACTAACCCTCTGTTGCAAAAATAA
CATCATCAACGTTGACCTTAATCGTTTCTTCTGCTGGTTTATTCTATAGTACAACCATAATATGAGTCAAATGTCCATTCAAAACTAGATTAGTG
TCTTTACTTTGTGCTATTAACCATTGATCAAGTGTATTAGTTGCCAATGTTATCACATTATGTACCAAAGGATTAATCTTGTTCCACACCACTTCA
ATTCAAATTCGCCAAACCACCCAACATAACAAGCATATTCTCCTCGTCATTTCTTTTGATTTCATCTACATATATGAAAGGACCAGTCAGCAAA
CACCCCCAACTTCCTATTGGTTTCCTCACATGATCCTTATTCCAAAATTCTTAAACAAATTAGCACTAAAATAATATATCTAAACCGGTTTTAGG
TTCGCCAAGACATAATCGACAAATTGAATAAAAGTGCACCTATATCATTTATAGATGACTTCTAGCAGGTAAAGAGATAGTGGATGGATGCCAA
AGTTTGCATTTTGTTGGTATTTTCAGGTTCCACAATTTCCTCCAAAAATACTATTGTAATTTGTGTCCCATTTTTTAAGCTCTTGAAGAATATTAT
AGGCAAACCTAATTAAATAATACCCTTGCATTTCTTTACTCCAATACAACCATTCTTCTTCACTGAATATGTGAAAGGTGTTTGCATGATGAGAT
TAAGATCTCTTTCCAAGAAAACATCTTTCAACAGAACCTCATCCCACTCACGATTGTCAAACTTCTTAGGCTCTATACTGGTTGCTTAAGTAGC
ATTGGATGTTGAGTATGCACATATGGATCAACATCATTTTGTAACGAGAGGTTTATCAATATATCCACTTCAAAACCTAACCCTATTAATTTTTCC
CCACCTTGTCTAAGTAATGGCTGAGATTCTAAAATAGATCTCTAGACATAACTATGATTATCACCAAATTTTGTTGTCATAAAAAACCGGTAGG
ATAGTATCTAGCTCTAAATAACTTCCCTACCATGCTCTCACTATTTTGAATAAGGCACCAAGCTTCTTTGCTTAATAAAAAAAAATTAAGATCTC
AAAGATTTCTAAACCCCATGCCCCCTTGACTTTTATAAATAGTCATTTTATGTGAACTCATCCAATGAATAGTATCCTCTTTGAATTAGAGCTTC
CCCAGCAAAATTAACTCATCATTTGTTGTTAATCATGACACATATCCTATGAAAGAAGAAATACATTCATTGCTTCATTCGATAAAGCCTGAACT
ACCTTCTTAATGAGTCTTTCTTTACCATCTCAAGACAATAGCTATCAATCCCATTTTTGTATTCACTTTCTCACCATTTCCTTAAAAATACCCAAT
AACTCACTCTTCTTTCTTCCTAACATACATAGCAATCTTAAATAGTTGTTGTTCTCACCAAACTCTTT[A/C]ATATCCATCAATGAGAAAAATCC
ATTTCATTTAGTCGTATCTATATTTGTACAAAAAATATGGAAGATTTGGCAAAATTGAGTGGACTGAAGCAACTCAAAAACACTAAGTAATTTT
ATCACAAATGTAGTAGCCTTACAATATAAACAACTATCATTGGTGAAAAGCATATGAAAGATTACTAGGTTTTATAGTGAGATTTCTCTCACCTA
GAAACTCAAGACCAGACTCCTCTTTAAAGGACACGTGTATTCAGATTTCCAATAAAAGCTGAATGTCCATGAGAGACTTCTCAAAGTTTAGA
CCTCGCTCAGGATAAAACCAGCGAGATACTGCGAGTGCGACTTAAGTCGCATCACGTAACCGTAATTCTCATCATGCTGGGTAGATCCAACTC
GCATGTGTCAGAACATGTGCGGGTGTCCCCTCTATATTTCCACGACAAGTATAGAGACCAACTCCCTATGATGCAGTTTGGTCCCAACAACCT
AATAGCCCTGACAGACCGATATAAATTCACATGTCCAGATTTTACTAGCTTCAACATGCGATATCTACAAGGGGCGATTACCTAAGGACGCAGA
CGTTCCAAACGTACTGGCGATCCTGCGAGCTCAACAAGCGGAACATGAGCGATCAATTCAGCGGATCACGGAAGGCGCATCGTTGATGAACT
TAGTAACTGTCACACATTTATTAATGTTAACGTTGTTTGAGTTTAATTATTGCAATTCAATGTGTAAATAATGGATTAACAATGAATGTGATCCTT
ATGTAACCGATTGGACACCTACTTTGTATATAAAGGGGTGATCCACCATGAAAATGGGACTACGAATCCTTTGTGACAGTGGACTAAGCAGAT
GGTAATCTGACTGAACCACTATAATTCTCTGTCTCGTTTACATTTTCCAGCCTTGTTGTTTGTTCTTGCATTCCCAGTTGAGTACTCGCCATTCT
AGGTTGAATACTCACACTTGTCATTTCCCTAAAATTCTCTTTTATATCAATTAAATAATACGTTTTGGTGTTGCGAAATTTACTCGACAACATTT
GGCGCCGTCTGTGGGAACTCGACTAAAAGATTTCATTCACTTTAATTAACACTATTCATCATGGCTGGAAATCACGCTAACGGAGCTAACAAC
GGATCCATCAATGTTGGAATGATGAGCGTACCATTGCCTGGAGCTGGAGTGCCACCTGTAGACGTCATCAACGGCGGAATAGGGAGGAGCAA
CATCAGACCTCTCAACCTATTCCCAAAACGACGGCGGCCCTCAAGTCTGGAGACACGCCCACACCACCAGCAACCATGCATTTTCCCTCGTC
ACCCGGCGGTAGTGTCCGAGGAATGGTCCAACTCACCACCGATCAATATCTTTGCAATTCGGGATCAACTGCGGGCACTACAAACCGAGGTA
GATGGATGAGTCGAGCTCGAGGCCCTCCTCGAGTTCACGCCATTCGCGACAACCCTCGGTAACGGTTCCAGGGCGTCAAACTAATGGGTATG
CGGGGAGCGAAGAACTGACCACGAAGCTCGGACTTGAATCACCCGATGTCAAGAGATCAATCTGCAAGGTTTCCTAACCGGGCGCACAAAT
CGGCGAGGATCTGGCGCGCAATCCTCGCGATCGTCCATCTCGAGACAACGACGCAGAATCGGCCTCTTCGTCCTCGCGATGGCGCCTTTCCG
GCGGTATACGAGGTCGGCTCTATCTGAACACAACGGGGAGGGCGTTATCATGTACATCAAGGTGATCCGCGTGATCACCTCAGCAGCTTTTTC
GGCACGCTCCGCGCCCGCATAACATCAGAGCGCTCAGTGATCCCCATGTGGGCGTCGTGGTGTCAACACGTAATAACCTTCTATCGCCGATCG
CGACTACCGGGATTAATATCCCAGCAAACCTTGCCGCGGTGGTCGCGAGCCCGTAGTCGCGGCAACTCCAGCCCGCGACGGAGGAATTGAC
CAAAGCATGCTTCTACGCAAGCTTTGAAGATGCTCGGCGACGGAAGCCTATATACAGGCGCATGGCACTTCACCTTTCTCGCAGAGGTGCGG
AAGGTCCAACTTCCAAAAGGGTTTCGTTTATGGAATCCCTCAAATATAAAGGTACTTCTAACCCGTTAGATTACTTAGAAAAGTTCAATACTAT
AATGGAAGTGGACCGAGTGTCAGCAACTTGCGAAGTGTAGCATTCTTCGCGGCAACACTTTCGAGGAGAATGCCCATGATTGGTTCACCAAT
TACGAGGCATCGATGTCGGCGTGGGAAAACTTCGTCGACTTATTCCTCACACATTTCAGGCCACAATGGCGTATAGGCCACCCTATCACGACT
TTGGCCAACATCAAACAAGAACACGGTGAGTCTTTCAAGACTATTTCTGGCGTTTTAACGCGAAGTAACAAAGGTCGAAGGCCCGAGTCTT
GGCTTGTTTGTATGTTGATTACAAGTATCTTGCCAAGAAACTTCGGAAGGAACTACAAGCCGAAGGCTGGAATCCTTAGTAGAGTTCTTCGCC
ATGGCCGAACCTCACAAGAGAATCGAGAACTCTCGTAGAGTTAGAGAGGGGAAGAACAAGCGGCGATCACCCATGCAGTGGTCGGCGCTCT
CGTCCGCTCAGGGAAGAATTCAGGGCGAAGCCCAAGAAGGCGTAGCCAGCGCAGACCACGAAGCCGAGTTGGCTAAGTCACCCCAAAGAA
GGAGTCCTCGTAGAAAAGGAAGGAGGACCTGCGCGTCAACTATCACCGAACCTAGGTACCTCGAGAGACCATATCTATGCGATTGAGGAAA
GGAACGGCGTCTTCAAGAAGCCGCCCCCATCGGAAATCGCGACCGGGAGGATCCTAAAAATTCTCGTAAGTACCACAAGGACATTGGTCATA
CTACTCTTGAATGTTGGTCTTTACGGGATGAAATAGAGGAACTAATCCAAAGAGGGAAGTTCGACAAATATAAAGAAGCGAGGAGGACATCC
CAAGCGGATGGCAAAGGAGATAGCCCAAGCGAAGTGGCTCGAGAACACCTCGCAATATCTTAACCATAATCGGAGGACCGCACATCGCGGT
GACTTCTGCGAAATCGCAAAGAAAGGTATGCGGAGAAGTTAAGGAGAGGCGGATTAACCAACGTGAACAATCTTGGTGAGCGGCCGGAGA
AGCTCTTCAAGAAAGAATGCGACGACATCGATTTTTATGGAGAGCGACGCGAGATGGATCCACCACCGCACTCGATGCACTAGTGATAGTCC
CCAATATTGGTGGAGACAATGTCCATCATTATCTTGGTCGACAATGGGAGTTCGCGTGAATTCTGTGAATTTCCAAGCCTTCAAGCAAATGGG
ATTGCAAGAAAAGGATTTGCGACCTATGACATCAAGCATCTATTGCTTCTCGAGAGATGTCATAGCAATTAAAGGGATGATCAAGCTCCCAAT

CACTTTGGGAGCGCCCGTGCTTGCCAAGTCGATGGTGACCGCGATGTCGACCAATACTCGGCATATAATCGCATGTAATTGGTCGACCAATTC
GAAGGAGATGAAGATCATAACCTCGATCTATCATCGGACGATGAAGTTCCCTACTCCCGTTGGAGTAGGTTCGCATCTTGAGAGTCCAATCGG
AGCTCTCGAGAATGTTATAACGCGAAATTCCAAACTTCGAGAAAAAGGGCGGTAAATGTTATCCACCTTTTGATGCGCCACCTCGCCGGAGA
TCCTCCGAGTCGAGGAGGTACCGCACATGGACCAACCGACTTGGATCCAGAATCCTCGATCACCGCCGCAGCCAAGCCGCGGAAGAGACAA
TCGAGGTACCTATAGATCCTATAGATAATAACAAGGTTTTAAAATTGGTTTTAGATTAAACATCTGATTCGCGAGAACAATTAACGACCTTTCTA
AAACAAAATCTTGATATTTTTGCCCGAAACATTCAGATATGGTCGGGATATCTCCCGGAGTCATGTGTCATCGCTGAACATTGACCCGAAGTGC
GAGGTGTCCGGCAAAAGCGCCGAAAATGGACCCCGCGAGGTACCAAGCGCCGAAGAAGAAGTTGACCGCCTCCTTGCTGCGGCTTTATACG
GGAGTCATTTTACCCCAACCGGTTAGCTAACCCAAGACTTGCGTGCAAAAGCCTAATGGCTCATGGCGCATTATGCGCTGACTTCTGAGATCT
AAACAAAGCTCGTCCCAAAGGCAGCCTTTCCTCTTCCTAGCATTGACCAAACTTGTCGATGCCTTCGCAGTCCGCAGCGCTTTTAAGCTTCAT
GGATGCATTACTTCGGGATACAATCAAATCCCAATGTATGAACCGCATTCAAGAACATACCTCGCTCATCATCGATCGAGGATTATACTTTTACA
AAGTAATGCCTTTTGTTTAATAAAGCAGGTGCTTAATTTATCAAAGGCTAGTAAATATGATGTTCGCAGATCTCATTGGAAAGACAATGGAGGT
ATATGTTGACGATATGCTCGTGAAAATCGCGAACATGCGGAGGATCATATTACACACTTGTAGGCCATGTTCGACATATTGCGGCGATATACAA
GATGCCTAAACCGCTGGAATGCGCCTTTGGAGTGGGTTCCAAAATTCCTTGGTTTTATGGTAAATCAACGAGGAATAGAAGCCAACTCGCGA
AGATCGGGGCATTGGTAGAAATGTCGCCACCGACTAAGCCAAAGGAGGTTCAAAGCTCCACGGGTAAGGTCGCGCTTTAAACCGCTTTATAT
CCGGATCCTCCTCGACAAGTGTAAAGAGTTTTTCCGACTTTGAAAGGCAACAAAAGGTTTCAATGGAACGAAAAATGAAAGCGAGCTTTCC
AAGCTTTAAAACCCATTTGGGGCAACCTCCAATTTTATCAAAGCCATTGTCAGGAGAAGTTTTGTCTATCTATTTGGCTAAGCTCGAGATACGC
GATTAGTTCAAGATCTAATCGCGAGGACCAAGGGCGCCAACGCACTCTGTATTACGTCGATGGCGATTATTAGGCAGCCGAGACGCTGCTATC
CTCAAATGGAGAAAGCGGCTTTCGCCTTTGATAATATCCTCAAGGAAATTCGCCGACCTTATTTCTAGGCTCATAGCATTGAAGTTTTAACAAA
CTTCCCTTAAGGCGGGTTTTAGCGAAACCGGAAGCATCGGGAGATTGTTAAAGTGGGCTTATGGAGTTAAGTCGGTTCGATGTCAAGTATAAG
CAGAGAGCTGCGATCAAGGGTAAGCCTTGGCGATTTTATACTTGAATTCCCAAGCACCGAGGTATTGGTTGTAGAGGGTGGAAATGACATTGT
CATGGCGAAAGCAAAGAAGTATGGACATTGTATGTCGATGGAGCCTCGAATAGCGAAGGTTCGGCGGCGGGATCTTGTTAATCGAGTCCCAA
TAATTTCAAGGTACCGCGGCGCTACGTTTTGAATTCTGCATCTAACAATGAAGCCATTATGAGGCTCTAATCGCGGTCACGGCGGCCCTCGAG
ATGAAAGTCGGTATCTGAAGCTTCGTGACTCTCAATCGTGGTATGCCAAGTGAATGGAGAATACCTAGCAAAAGTGGAAGATTGGTTAAATAC
TTAGCCATCGTTAGGTGAAATAATCTGAAGTTCAAAATGTAGTTGTGTCTCAGATAAGGTGTGCTCATAATGCCTGGTGCATTGGCCGATTGGC
TGAACCAAAGCCAGAATTACTCGATGTAATTAGGTAGATGTATTGGCTCCGCAACCATAGATCGAGAAACAATCATGGAAATCGATGATGTTG
GAAGATTACTGGATGGCTCCTATCCTCGCATACCTTGAAAGGGGCTCTTGCACGATGATAAAATAGAGGCGAGGAAAGTGCGGACGGTGGCG
GCCGATTATGTGATATATGACCAAAGGTTGTATCGCGAGAAGTTTTAGTCAACCACTTCTCAAATGTATCGATTGGAGAAGACTACGGTTATCT
TGCGTGAAGTATGCGAGGGATTTGTGGCAATCATGCGGAGGTAATTCCTTGCCTTAAAATCATCGCGGCAAGGGTATTCTTGCGTAATATTGCG
ACAAGATGCCCTCGCATTTGCAACAAAGGTGTGATAGATGTCGTGAATAGCCACATACACACCGGCCCGAGTAACCTCCATTCTATCACAAGT
CCTGGCCCTTTGCAATATGGGAATCGATTTAATCGTGAGTTACCCGAAGGGAAAGGCGGAGTCAAATATCTTTGTAATCGCGGATTGATTATTT
CACAAAGTGAAGTGAAGCAAAAAGCACTTCGCAACCATCAACGCAACAAAATTCGCGAGTTCGTCTACACCTCCATCATTTGTCGTTTTGGC
ATTCTGCATAAACTCATTTAGGACAATGGAAGCTTGGTTTATTTGTAAAGAGATCGCGACGACTATCGACGATTTGGGGATTAAAAAGCTTTTC
ATGAGTTGCTTACCCTTAAAGTAATGGATGGTGAAGCGTTAATAAGATAATTAAACATACCATAAAGGGAAAATAAGAAGGTCGCAAAGGGG
CTTGGCGGATGAGTTATCGCCAAGTCCTATGGTCTATAATCGACCCTTGATCTACAAGCTGGCGAAACACCCTTCTCACTTTCTTCGGGTGCGA
GGCCATGTTGCCGATTGAATTAGGGCGGGGTTCCCTGCGAGGGATATGTTCAACATCTGCATAAAACAACGAGAATCGGCTATTACGCCTTGA
TCTTTTGGAGGAAAAGCGTGATAAAGCGCAACTACAAAATGCGGCTTATCAACGGCGACAGCCAAGATGCTTTAACTCCAAAGTGAAGGTA
AAAACCTCGCGAAGCGGAGACTTGGTCCTTAGAAGAATAATGCCAAACACCAAAATCCGTCATGGGGGTTTTGGGGATAATGGGAAGGACC
ATACCTCATCGCGAGATCAAATTGGTGATGCGACTTATCGACTCGCAACACTTTCGATGAACTACGCGATTCCACGAGCGTGGAATAGCGAGT
TTGAAATTTTATTATCAATAATATTAGCATTATTGATTATGTTAATTCTTGTACTTATACTTAGATATTTTACGTTCAACAAGAAAAACCCTAAGTA
TAGGGGTATATGCCGAATGTACTATTGATTATATGAATAAAATTACTTGCTAAATTTTCAAGTGATTTAAATTTAGTTTACCAGCGTTACAAATTT
TCCTACTTATTACACCAAGGCGTAATTAAAATTCGCAAATATATATAAAATTGCGATTACACAATTTCGTAATCAAAGTTAAAATATATATAAAAT
CGCTGATGCGCGGGTGTCTTGAGTAAATATTAAGGATAGCTATCCCGAGGGATAGAAATTTGTCCAAAACAAAGGTAAATTCGTCTAATCTGA
AAGTGCAGTACCCTTGTGCCGCATATATATAAAAAAAAAGTGAAATAAAAAACAACATAAATCGGAAGATAATTAAGCATCACGGGCGGCGA
GAGTAGTCCCATCGAGGAGCGCCTCGTCGGCGACTTTCTTTGATGACCTCATTCTCGACTTCTTTAGACTATACGCCCTCGACCTCGTCGAGA
AGGTTGCCTCCCCACCTTGAGTGCGAGTAGGCGACACGGCGAAGGCCTCGCCATCTCGGCGGACTTCGGCTCAAGAAGAGAGAGTCGAAG
TCGCGGGACTTGGAAAGAGTGGTATAGATGTGTCGGACACTTGCTGATCATCTTGCTTCTTCCCATTCTCTTTCTCGACCTCCAAATCGCGAG
CCATCTCCGCGATCGTTGTTTCGCGACTTCTTGACCTCGAGCTCGCTGTTCTTTCGCCTGTTCTGAGTTCCTCGAGCTCCTTCTCCTTGGCCTT
GTTGAGTTCCTCAGCTCCTTCTCCATCCTCGCCACATCCCCTTCGGCCTTTTTATATTTTCACGAAAATGAGCGTTACGCCTCTTCGGAAATCG
GCTTCCTTCGAAGGAGGGGCAGCTTTTATGAACAAAGCCATGGACTGCGGCCAGTTCGATGGATCGAGTAACGAGATCCTCGTAAGGGATCT
CGACCAAGAACTTTTCCCTGCGATGGCGGGAAGTCGCGAGCGCTGAACAGGAGGGTCAATGATCGACTTTCTTCCTTTGTCCGAGTGGTCTT
GGCCAGCTTCTTGGAAGAGTCCGCGACAAGCAAAGCCATCACATCGCTTCTTCCTCTTCCGGCAACGACCGGTCGAAGTCGTCTGTTCCACC
CTTTGGTTTGATCTTTCGTGATGAGGGAGACTTCGTAAAAGTCATGTCCGCGAACATGAATGAAAAGATAAGTATAAATCACAATCCTACCCG
TCGGTTTATAACAAAAGACGAAGTTACTCGAAATTTGTCAAGGAGTTTGCATGAAAGACGCTTTGTCTATTCGCTCTTTTGTCGCTTCTCGGAT
GGTTCTTTGTATATCTGGCTCCGTTGAAGACTTGCGTTCGAGGAATCACCGATGTTCTCAACTCGCAGCTTGTAACAATAATCGCTACCGTGAT
CTTGTACCGGAAGGCTCTCGAGAATTTCTTTGCCTCCTTGCTAGTCGCGTCAAGAGTCGGTCGAGCTGGTCTATCTGCGATAACAACAAAAA
GCAAGTAAGCAAAAGATTTCAAAAGCAAAGCAAAAAATGTCTAAGTCAAAAAATACGCGACATACTGGGTCTGCGATTAAAGTCGGTCCTA
ATCCCAGGAACCTTAAAGACGTAAAACCACTTCGACTTCCAATCACCCATGTTCGACACCATTCCCTCAACACCGTTTATTTCAGAAGTCGCC
CATTTGCTAAAGCAGTAGAAGCCATTATGGAGACCTACGCTCTTTATGTCGTAGAAATAACTGAATTCTTCTACCGAGGGAGCCCTATGAACGT
ACTCCATGTACATCGCATAGAAAGCCAGGACAGTGCGATAACTGTTGGGAGTTAACTGAAACGGCGATACGTCATATCGCCTGAGAATGGCT
GCGACGAAGGGATGAAAGGGGACCGATACACCACATCGAAGGATGGGTATTGAGATTACCACATCCTCTGTAGGGATGATCGCAGGGTTGGT
GAATGGAAGGTGCGCTCTCTGAGATGGTTTAGGACGTTGAAACGCAAGTCGCAGCAAATTCAACCTCACGAAGGTGGTGAAGTCTGATTTG
GCAACCCTCGAGACTAAATCCTCGCACGCGAAGGGCTCGTTCAATTGGGAGTCGCCGACCAAATCGTTCCACTTCTTCCGCTTCCTCCTCTTC
CCCACCGACTTCGCTGGTTTGGAGTCATCCATTCTCCTCCATCTCCTCGACCTCGATGTCGGGAGCATGCCTCGTATGTATAAGATAGTCGTGT
CGACAACTTGTGGACTCGCTGTCTCGGATATCGATGGTCCTGTGTTCTCGTGAGTTCTCGCATTCATCCTCTCGATGTCTACGTAAGACATCGG

ACCTAGTTCTATTTGGCGCGGCCTCCATTTCGGAAATTTCCGTAGGAGAAAGCTGTCTCTCCTGGTCCGACTCACCGTCAAATGCGTGGCCTC
TGAGCCGAAATGCCGGCTATCCGATAAATCGCTCATCCGAAGATAGAAGATCTTTTTATTGATGGATGTGTGAAAAATAAAGTAAGTGATCTCA
CCCGATAGATTATAAGGTGCATTCGACAGAATGTTCACCTCCATGCGAATCTGACCATCCCGTCAATGTGCGAAAAGATACAACTATCTTGGGC
AAGTAATTCCATCCTCGATTAAATGGTATACCTCGAGAAGCGGTGGAGTTTCCGGTGACTCAAGAGGTATGCATTTTCGAAAGCATAACCCTG
AAGTTACTGGGAGACCCCCACCATTCGAGACTACCTATCAACCAAAGCGTGCGATCATTCGAGTATTAAGCGCACGTCAACAAATGGCTGGG
TGTATCCGCGAGATCTCAAAGGCATATAATCGCATATATGGCCGTTAGAATACTGTGACACACCCACCATTTGGAATGGCAATTAATACTCTCAC
AACTCGGCCCACGACATATAAAAATCTTAAAAGATCTAGCTAATAGTCTAATGGAAGTAAGTCTTGGAAATATGCGATTATTCAAGCTGTTCAT
CTGAATACCCGCGAACATTCAAATTGTTCATCCGAATATCCACGAGTATTCGAGATGTGAAACAATACCTATGAATTGTGTGCGGTTATGTCAG
TTTATAGGTCCTATTCTATGGATGTTACCCAGTTTCACACATAACCTCATGAACACCCACTAGCATACATTCCTAAAAAGCCTTTAAAACACCC
AAACCCAGAAAATAAAGCATATTTTCCCCAAAAGCAGAAAACGAAAATGCAGTGATTTAGAAACTAACCTTTAGTTCTGATTCCTGCGATTGC
TCTCGATCACTTCTGAGATTGAAAAAGTTGGGGAAAAAAGGGCAGAAAATTTGGGACTGTATTCTTTCGAAGTGTATGCACTGGAGGGAAGT
TAAGAATAAGAGGGAAAATGGGAAATTTGATTCGTATCAAAGCGCTTAAATACCCATATCCCTTATTAATTGGGATTGTGACACGTGGCAGCC
AGAATGCCTAAGGTCGCCCAATCTAACGGCCAACTATGGCCACGCTTGCACGAAAACCGAAGAGTTTTGTGACGTGGCACCCTAAAGATAAT
AAAAACTAATAATTACAGCCGTCATTTTTCGAAATCCTCGATGGGACAACCAAAAGGTCACCTCCTCGTGACAACCTTTCACCTGTCTCTCGA
GTAATAGTTTCCCTCAGTGACCGCTCTTGTCACATCGCGAAACTAGGGGCATGTGTTATAGTGAGATTTCTCTCACCTAGAAACTCGAGACCA
GACTCCTCTTTAAAGGACACGTGTATTCAGATTTCCAATGAAAGCTGAATGTCCATGAGAGACTTCTCGAAGTTTAGACCTCGCTCAGGATAA
AACCAGCGAGATACTGCGAGTGCGACTTAAGTCGCATCACGTAACCGTAATTCTTATCATGCAGGGTAGATCCAACTCGCATGTGTCAGAACA
TGTGCGGGTGTCCCCTCTATATTTCCACGACAAGTATAGAGACCAACTCCCTATGATGCAGTTTGGTCCCAACAACCCAATAGCCCTGACAGA
CCGATATAAATTCGCATATCCAGATTTTACTAGCTTCAACATGCGATATCTACAAGGGGCGATTACCTAAGGACGCAGACGTTCCAGACGTACT
GGCGATCCTGCGAGCTCAACAAACGGAACATGAACAGTCAACTCGCAGATGCGGAAGACGCATACGTTGATGAACTTAGTAACTGTCACAC
ATTTATTAATGTTAACGTTGTTTGAGTTTAATTATTGCAATTCAATGTGTAAATAATGGATTAAAAATGAATGCGATCCTTATGTAACCGATTGGA
CACCTACTTTGTATACAAAGGGGTGATCCACCATGAAATTGGGACTACGAATCCTTTGTGACAGTGGACTAAGCAGGTGGTAATCTGACTGAA
CCACTATAATTCTCTGTCTCGTTTACATTTTCCAGCCTTGTTGTTTGTTCTTGCATTCCCAGTTGAGTACTCGCCATTCTAGGTTGAATACTCAC
ACTTGTCATTTCCCTAAAAATTCTCTTTTATATCAATTAAATAATACGTTTTGGTGTTGCGAAATTTACTCGACAACACTAGGGCCCCCTAGTCA
CCTTACAGTCATGAATCAATCCTCTCATCTCATAAGCTGAATTAGGGTAGAGAAATCTTCTACACAAATCAAGGATAAGTATGGTGAAATTGGA
TCACCCTGTCGAACCCCTTATTAGAACAATTTGCCCTAACACAACCCCCTCATACTCTTATGTAAAACTATTGAAAGGCACTGCTAAACTATTT
GAAACTGTTTCTACTAAAAACCCATGCGCCTTAACATTGCTAAAATGAAAGGTCATTCAACTCTGTCATACGCGTTACTCATATCCAACTTAAG
AGTCATATAACCCTCTTTCCCTTTATGCTTCCTCTACAAATAATGCAAGACTTTAAAAGAGACCATGATGTTATCTGTGTTCAAATGGCAAGATA
TGAAAGTACTTTGAACTCTAAAATTATACCATGAAGTAAATGATTTAACCGATTAGCAAGGACCTTAGACACCACTCTAAACAACACACTACA
TAGAACCATTTATTATAAATCTCCCATTCTTTTTGGATTTTTATGATTTGGAATTAGGACCACTTTAGTATCATTAAGTCTCTTCAGAAGAACATC
TGTGAGGAAAACATTATGAGCACTCGAAACCATATCTTTACCAACAATATTTCTGATGAAAGGCTAGATTCATGCCATCAAGTCTTGGTGATTT
ATCAATATGTTTTTTTGAAAGAGTGCATACTTTACCTCCTCTGACGACAATGATTTAATCAATACTTGGTTCTGGATGTTTGTAATATAAGGGTT
TATTAATTCCACAATCACTTCCTAGGTAGATTCTGAGGCTGGGAAAATATTATTAAAGTAATTGAGCAATATTAGATAAACAATTTCTCCAATCT
ACCCAATTTCTATCTTCCCTTTGTAACAAATTAAACTGATTATTTCTACGTCGAGCACTAGCAGTAGCATGAAAGTACTTATTGTTTTGATTTAT
GGCTTTCAACCAAAGTTTTTTGATCTTTTTTTTCCTAAAAACTTCCCATTGATGAAAGACTTCAAATAACAGCTACCTTTGCTCCTAGTATAAC
TAGATCCCCTCTTCATCAATTTTGTGTTTCAAACTCTTGAACTTCTTGTTATAATTCTTTATACAATCTTTGAATTTTCTATTGGTTTTTTACCCCA
TTCCCGAAGCTCCTCTCAACAAAAATGAATTTTATCTTGTATCAATACATCCATATGAGTATCTAAAATATTTTGGATAGTTTGTTCACACATTGG
GTTACTAATACAAGAATTTTCAAACCGAAATTTTGAGATTCATGAAGTAGGTAATATAAAATATAGCTATAACAGTTTAGTTGTATGATCTGAAA
AAGAGATTTCCAAGTTAAATAATTTGGCAAGAGGTTATAATGTAAGCCAATCAAGATTTACTGATGCTCTATCCAACTGAATATCCACCTATGA
CACTTTACCCTTATCCCTCTCCCAAGTATATTTATACCCAGTAAATTCCATACAAATCAGACTAAAATCTAGAAAGACATGTTGAAACCCTTCCA
CCAACCAATTCAAATAAGGCTAGCCACCTTGTTTATCTCCATGGCTAACTATAGTGTTGAAATCCCCTATCATGGCAAACACCTTTTAACATGA
AGCTATCTTAACAAATTCCAAGTCTCTCTTCATATAGTTTGGTTTGGTTCACCATAAACCCATGTTTGCTGATATGAACTATATCCTTCAGTAGTA
ATTAATAAATCAACATAATTGCGTGAATAACCCAAAAGATGAACTTCCTCATCAAACCTCTAGAGAATTTTTAAACCCCCTATTTGACCTTGAG
CATCAGTTATAAAACAACCTTCAAATCTCAATTGATGCTTAATGGATTCAATATTTACCTTATAACATAATGTTTAACATAAAAAAAAAAAAAAT
TAGGCTTCTTTTGAACAGAAAGTTCTTTTAGGAATTGAATAGCTCGTCGGTTTCAAAGCCAATGACAGTTCTACGACAATCAATAATCTAGAA
AAGCCCTATCTTGAAGGCCCACCTCTAATGCATGTTTTGAATTTTCAATTGTACAGTGAAGTGAAACCTCCTCTTCATAGCCTAATAAACTTTC
CATACCCCCATCATCCTGATTGTTGGGCCTACCTCTTTTTTATCTTTAATTACAAAACCCTTTTGTTTTACAAAACAAACATGCCCACTCCTTCC
TTTTCTCTAGAAGTATAAAATGAATTTCATTAACTTCCTAACTTGACACCCCCCACAATTTACTTGTTCCTCAAATTCTATCTTGACTCTTTCCC
ATAATGAGATCATGATCACCTTGATTTGTACCAATTCTTCATTCATAATTTTATTACCATTAGAATCTCATCAATTTACGATATTTGTATGATCCAT
GTCTGGCAAATAACTGACCTACTCTAACTAAACACATATTTTCGTCCGCAAATATTTTTTTTTCTGATTAAATGATTCTACCTGTGAGGTTACAC
TTTAATCCAGGAGCCATAAGGATTCACAATCTTCTTAATTGGATTTTCAAATAAACTTGGTAAAAACTTTCGTCAGGCCCCATTAATCCACTCA
TATAAATGAATGTCAGAATATTCTCATATTTCAAATAAGCCTAGAACTCGTCACTTCTTTCTTGTTGTATATATCTTCATTCTCCTTCAGAAAGGT
TTATGGATATTAAGTGTAACACAAATCCTAAAAAATCCCTCCAAATACTAATAAAATTATTTTTATTAGTTTCAGCAAAAACACCAAGTTAATTA
CCAATATTTGTTATTACCCTTTCAGACATGAATCCTTGTTGTAGTTCAAAAATTTGTACCAAAATATCCAACATATCAATAACAACAGGTCGTGA
ATTCTTACCTCCTTTTAATCGTTTGATCAAAAACTATTTCCAATTATAGATCCATGGACTATCTACAATGACTATCCTAATATCAATTTCATAGCAA
AACTGAAATAAGTATCGATGATGTTCTAACATCTTTATAAAAGCCTCCTTTAGCTAGTTTCCATAAAGAAGTCAATTGGTGTTTCTTAGTGTTGA
AGTCAATATCACCATTCGTCAAAATCTACCCCACTAAACACATTCTCATATCCACACCCTCAGACCACTGAAATGATTGATAAGAAACTACATC
CTCTACTTCATCTTTAAAATGAATATTATCATATTGTGATTCTAAATTTGATACCCCTCGATTATTAGAAACTACATTATTAAAAGAAGAATCAAA
GGATACAACACTAAAAAAACTTATTTAAGAAAAAAACTATATCAAAAGACAAGACTTTATATTTAGCATTTTATTAAGAAAAAATTCTAATTAT
TTTTTCATTGGTGTCATATATTTAACAAATCTTTAATTGGATATTTATTAATACATCTAAAGAAATATATTTAAATAATTTATGAACATATATAGCTA
GCAAGTAAGGATTGTAATGAATAAAGTCATCGAGTAAGCACTATACATTAAATAATAGTAAAATGAAATTATTTCATTAATTTATTTTGCAGAA
TGAGCATATGATTGATGAATTTTCTCATCTTCGAGGGCAGGTACATGTATTTTTTATTCTTTGTGTATATATATATATATATATAAATGCTATGTTTG
TGGATGAGTACTATATTTTCAATACTATTGACTCATTACAAAGGTTTGAGTAATTTGCAGGAAGAAATGTTGATGGAAGAAAACAACAATTTAA
AAAATAAGGTATGTTTCAATTCCCTAACCTATACCTAGTTGTATGATAAAATTAATTATGAGTGAGAAACCTCAAAGAAAAACAAATAGACCTT

TAAATAAATTTGCTCACAGCTAAAATATAGCAGAGAAAAGTAGGTATGTTTCCAAAAAAAAAAAATTAGGAGTATATTATCCCTCTTCTATATGA
AATATTCTTAGTAGTGGACCCTTCATGCTAAAATGATTGGACTATAGACTCCTTACTTATTAATTGCCATCCAAATGATTTTCCAGCCTAATCTTT
TTAATATTCATATACGTTGTAATGATTTAGGACCACTCGTAAATTTGTGATAAATGTCAATGATTTATAATGCCAAAAAATGGCCATGTTAGGATT
TATAATAAACTCAAGGAACATAAAATATCTCAAAACCTAATCATGTTACTAAAAATGCTTTAATATAAAAACTGTAAATCATGAAATCCTTGTTA
AATGAATAGAAAATGATGAATACAAGAGTGAAAGCACTAACCTCAAACCATGAATAGAGATTGCACCGAAGGTTTTGATCTTCAAATAATACA
ATATTTTTATGTACTTTTCTTTGACGAGGAAAGATAGAATACAAGAGTCTTATTGTTTCTTGGGGACCACTACTTACGTATATAGAAAGATTATA
GAATTAGTTTTGGATATTTGTAATTTAATTCTTAATGAAATTTTAAATTAACTTATAGAATCTTCTACACATTATTTCCATGATAATTTAATAACCTT
TATTACTTATACCATAATTGATCACTTAATTTTGTATCAATCTTTATTAAGCATTATACATTATACATATTTGGCAACAAATATAATTTTTCTCTTAC
AATCTCCCAACACTGTTACAACATCTTAATATTTACATACTTGACATTACATTAGACTCATAGTTGAAGCATAGGTACATTTTTGCATATCTTGAA
TTTCAAAAAACTTTTAGTGTGTTAATTAAGATTGAATTTCTCTCCTTTTAACAGCATGAGTAGCACCTGGTCTACAATCTTGCTTGCCAAACTT
TTTTAGTACTTTAGTGATATTGCTCTTTTGTAATAATCCAAGAATACCTCGAGAATGGTCTCGATGTATTTGAATTTCCAAAACAAAAGAGGCAT
CCCTAAAATCTTTCATCTCAAAATACTTATATAGAAATCTCTTAATTTCGTGTGATAAACATATATCATTAGTGAATAGTAATATGTCATCGACATA
TACAACAAGGAATATATACTCACTCCCATTAAATTAGTGATATACACAATATTTATCTCAAAACCAAAAGAGATAAGTACTTGATGAAACTTGT
GATACCATTCATGTGAAGCTTAGATTTTTTCATTTTGCAAACCATATTCTTTGGTTCACGTAACAAAAAGTAGTTTTTTTTTTGTTTCACCATATA
ATTTTTTCTCATCAATATCACCATAGATATATTACATCCATCTAATGTAACTCAAGATTAAGATGAGCAGAAAGTTCCATTATCATCTAAAAAGAG
TCTTTCGATGAAAGTGGAGAAAAAACCTCTATAATCAATGCTTTCTTTTATAGTATATCCTTTAGCTACAAGATCTGCCTTACACCTCTCCACAT
TACCATTTGCATCCCTCTTTGATTTAAATATCCCTTTACAACCAATTAATTTTACAATTTCGAGTAATTGGACAAATTTCTAAACTTTATTGTCTT
GCATATAGTATATACTCTTCATTAAAGAACAAATAAACTTTTTAGAATTAGAACTTTTTGTAGCCTAAGGAAAGTCGATTGAATAATATTCCATT
ATTCCATTGTCACATTCTTTTTTTTTTTTTGAAGAAATATAATACAATCATCTAAAATTACTTCTTACTTCTCTTGTGGATTATCTTAATGGCTCTT
GTTCTTAAACATGTTGAGTTTGTTCTTCTAGAACAATTACCTCATGTTCAAATGGGGGGTTTGTTTAACATATATTGTGTTGTTAAGGTTTCATA
TTTATTTCCTTATATAAATGATATGTGTGGAAGCTTGAAAATTTTCAAAATGATAGTTGCAATAGCGTTTTAATTCAATTACTCAAAATAAATGTG
TCTAATCTTATTTATCCCGAAACTCCACATCTTCAAATAATGTTGCAGTTCCCGTCTCAAAAATATTTTAAATTGTGGCTTATACCCCTAAGATCA
TTAAGAATAGGCAATAAAATAGTTGCTCACTATTTTTGAGTCAAATTTCTTTTCATGTGGCTTATATGCTCTTTCTTGAAGTGGACATCCCTAAA
TGTCAAAACTGTTTTAGGCTAGGATTTTGACCTGTCAAAAGCACATAAGGTGTTTTGTAACTGTCTTAGTTGGTATCCTATTAAGAATGTTAGT
TGTTGTCTTTAACACATCTCCCCAAAGAGTAACATATCCTAAAGAGTCCCATTTTGTCATTAATGTAAAACACTCAAGCAACTAATAATATCTC
AACAACTAATCATGCTACTAAAAGTGCTTCATTATGAAAACTTAAATCATATTTCTATAAATTAAACCTTTTGTTGCGAAAATTATTAATTACTAC
GCAAGTGCACGCAATCAAGTAGTATACTCACGCAAGTGAGGTCGAACCACAAGAAATTGGATTAATTACTACTAAACTATACTTATAATTCTAT
TTGGCAAATCAAAAGTATTTATGATTGAAAAAGGAAGAAAGAAATTCAAGAAACTTAAAGGAACAAGTGAATATTAATCAAGATGAGAGAAT
AGGGAGACGAATCCTGTTGTTAAGTTACCAATGTTAATGTCTAATTGCTATCCTTCTCTTGAAGTGAATGACAGATTATAAATTAACCTAGCTCT
TTTCAGATCTTCTAGGTTCTAAATCTCATGCTCTCTAATTAATCTCTTAATTAAACTAACATGAAATCAGCATTAAGCAATAATCTAAATGTCACA
AAGGTTATGTAAATACTTTCGTTTCACATCAAAACCTAGACTATCCAATTTTAGCATTCTCAATTCTCACTTTTCTGATTTTGAATTGAGATCATA
AAACATGTAAAAGGAGTAGTGTTCACAATCAAGATGGAGGAATGGCAAGTATTCATTAACTAGAAAAAACTTAAACAACATTCATCATTCTCC
CTAAATAGGAGTTTAGTTCAAAAAATCCATAATCAAATCCATAATTAACATTGAAACAGAAAATAACATAGAAAAATTAAAGAGAAGAAAAA
GAACTAGTTGAAGCAATTGATCCGGGTTGCCACAAGCCGCTCTTCTAGCCTCCTCCTTTGTTTCTAGGGTTCTAATTCTGAATAATCCCTAATT
TTTACGAACTCTCACTATTTAAACCAAGTCTCAACTTAAAAATTCGTGAAATTACGAAACTGCCCAAAAATCCGAGTCAGCGGGTCCCGTCA
AGGACCAGAAGCTCCCGTCAAAGTGACGGGATTAAACTTTGAAACTTCAAGATCCTCTCTGCCAGTTCCCGTCGCGACTGGCATGTTCCCCA
TCGCGATGGGAGTAAACTTTGACTCCTTGAAAATCTCTCTGCCAGTTCCTGTCGCGACTGGCGAATTCCCCGTCGCGACTGGAACAGGCAGC
TACACCAATCTTGGATTTTCTTCTCGATTCTTTCACAACTTTTCCTCATTTCTTGAACATACTTTCTTTAAATGCCCGAGGACCTGAAACCAAA
GAATCAAGCGTAATATAGCCCTAAAACTAGAAACAAAGAGTGAAAACTAACCGAAAATATGACCCGAAACTTAGACTAATTTTAGCCTAACA
CCTTTGCTAAATAAAAAAAAGATGATGAACACGAGAGTGGAAGCACTAACCTGAAACCATTAATAGAATTTGCATAGATAGTTTTGATCTTCA
AACAATATACTATTGTTTGATGTATTTTTCTCTAATAGGGAAGAAGAGAATACAAGAACCCTAGTGCTTTTTAGGGACAACTACATATTTATAGA
GCGATTATAAAATAAGCTTTTTTTTATGAATAAGAATCATTATATTGTTCAACTTCCAAAAATATTAACCAACCACGTACAACCATATCCCAAAA
AAAAATTCAAAAACTATACACAATTTTTGACATAACAAATTGAACCAATCTCTATCTATTGTATGAGCTTTTTTATTACAAGAGTAATCATAACT
TGTATATCTTTTTCAACCATCTATAATGTGTTAATTATATGCCATATTATATGGTACCAATATACATCATTCCCAACTCTCGAAATTTGGTAGAATA
AAGCCGCAAGAGTAACACACATGATACTCCTCTTAGTAGCACTCATCTTCTTTGCTTTATGAATCCACCTAATTAATTAGAATAAATCCACAGA
AGCAGATTTTTATTCCATTCAACACTTAACTTCCTACAAACAAGTCTTCCTATACTGACAATAAAAAAAATAAATGACTTATGCTCTCATTATCA
ACAACACATAAAAGACAAATTTGATCAATTAGAGGATCAAACTTTTTTATTTGTTCTCTTGTAAAAGACCATTTTATGCTTCGTGATACTCAATC
CATCCTAAACCACCTTATGACCCACATCAAGAGATTGCTAAAAAAAAATAAGTCATGGCCACTTTTAAAGCTATACCTAGGTCAAAAAGTGTG
GCAAATCAATCTTGGTCCTAAAAACATCCTTGACATCAACTAATCTCTTTTAATACAAGCTACATTCAGTTGGTGGCTAGTATTCCAATCCATTA
CTGTCCATCAAGTAAACGCTATTAATCCCTTTAATAAACAATGAATCCTTTTTGGTGGCAATTTCCTACACATACTTCCCGATTGCAGCCACATT
TCAATCAAGGACCTTCCAAAAGCCTAAACCTCATGTATTCTTTGGCAAACAAGCATTTTTCCTAGCCACTAAACCTGGTCAGTAGTGTCGGCT
GGGCCTTTTCAAAGACAAGCCCTAAAAATGGCTTCTACATCCTTTAACAAATTTTTTGGGATGATCATTATTTGAGCCCAATACGAGTGTATAG
TTAAAAAAACAGAGCTGATAAGTGTAACTCTTCCCATGTAATAAGATAAGTTTCTCAAACACAACACTCGGATTTGACCAACAATTTTCTCAA
GGATGCCATTACATTCACTAGCTGATATTTTCTTTGAACAAATGGGAATGCTAAGGTACCTGAAAGGATGATACGCTCTTGTGTATCTTGAGAC
ATCAAGCATTCAGTTGACTTCAAATTCATGCATTCCACTACTATTAATCTCTAATTTTTCTTCTTTTGGCAGTAGCCCAGAAGGTTAAGAAAGT
GACTTTAAACCCTTCAACATTAGCAAAATTGAGAGAAAATCACCATGGGAAAATAAAAGGAAATCATTCGCAAAACACATGTGATTCAATTTT
AAAGTAGGCATCTATCATGGAATTTAAAACCTTATCAAGAACCCACTTCCTTCAGTATTCTATACATGTATTCCATGCCTAGAACAAACAATAG
AAGAGACATCAAATCTCCTTGTGTCAAATCCTTTTAATTTCACCATTAATCAATAAGGAGAATTTGGGGGTTTTGATACATGCCATAATAAGATC
AATGAATTTTCAAGGAAAATGAAAATTAACAAACAATTCTTCAATGAATCCTCATTCAATGGTGCCATATGCTTTCCTCAAGTCAGTTTTAATC
ATGCAGCTAAACTTGCAATTCTTCCTTTTATAGTGCCTAATGAGGTCTTGAAAAACCATAATATTGTGAGCTATATATCGCCCATGAACAAATTG
ACCTTGGTTCTCCGTGACAATTTTAGGGATGACATTCCTTAGTCTTGAACAAATGACTTTAGTAGCAGCCTTATAGATCACTTTACAATAAGAA
ATTGGTGAGAAATCTTTAACACTATCTGGACATTTGATTTTAGGGATGAGTGTAATGTTTGTTTTATTGATTTCTTTAAGAATCTTCCTCGTGCTT
AGGAAATTATTCACAGCCTTTTTGACATCTTCTACCACCAGCTCCGAATTATCATGATAGAATGAACGCCCAAACCCATTTGGACCAGGAGCC
TTCATACCAGGTATGGAAAAAATGGCATCTTTCACTTCTTGTAAAGTGAATAGCTACTGTAGAACTTGAATGTGCTCCGATGAAAGTACAGGA

CCAACACGCACAACCGATTGGAACACCTTATGTCTATTGCTCATTGAAGTCCCAAACAAGTTCTGATAGAAATCCAAGAAAGCTTGTTGAATC
TCCTCTGCATTATCAAGCCAAATATCACTCTCATCCCTGATAGAGTTAAATATGTTTGAGTTCTCTTGGATTTCAAAGAAGCATGAAAATGGAA
GTATTCTCATCGTCATGTTTTACCCAATTCATTTTTACCTTTTGAGCTAATAACAAATTGTGAGCTTTATGGTAATGAGAATACTTATCTCGTGCC
ATTTGTTCTTGTTCTATCAAGTCTCTATTCTTAGGATCTCTATCCAACTCTTCCTAGATAGCTAACATAACTTGTTTAGCTTGCATATTAGCCTTGT
GAATCTCATTAAACTCATCCATGGTTATAGTTTGGAGCACTTTCTCAAGCCTCTACAAACACCCCACCAATTGATACATAGGAGAACCTACAGT
AAGGGCCATCAAACTTATCCTAATCTCTTCTTGATAATTTCTTGTTCTTGTCCACATGCTTAAGTATCTAATGGCACCCTTCAGAAGCTTGAATC
TTTATAGAAAGGAAATGAGGATAGGACTATGATCAAAGTCCCCTTTCAGAAGAAAAAGTTCTTCTAAATTCAAGAAAGAAACTATCCATTTTG
AGTTCACCAATGCACGATCAATGTTAGAGAAAACTCTCTCATCAGGCTGCTACTTGTTATTCCAAGTAAAGAATCATCCTGAATATTTCAATTC
ATCTATTTGATAATGCATCACCCATTCCTTAAACCTATTAGATGGCTTCTTGTGGTTCCTTCTATCAGCTCTTTCATCATAATTCAAAATCTCATTA
AAGTCACCCACTATCATCCATGGTTCTTCATTTCCATTCTTAAGGCCTTTCAAATCTTTCCAAAGCATATCTCTTTCCACTTCCTCATTAAAGTC
ATGCATACACAAAACTAATATGGAACTTTACTGACTTCTAATTTTTAAGCAATACAATGAATGAATTCACTTGTACACATTCTTATATATAGAAT
ACAAACAAGGATTGAAAGCCATAATTATTCTCCATTTATCTAACCAAGGGTTATTATTAGTGAAATACCAACAAGGAAATAACTTTAAATAAAG
CTCACCCATATTCTTATTCTTTACTTTGGTTTTGAGCAAACTAACCAAATCAACATTCTTTGAGTGTATTAATTGCCTAATGTCTCAATGCTTGTG
CTGGCTGTTAAACCCCGCAAACATTCTAAACTAGAATTCTATCCATTTGACGTAGAAGGATCTCCCCCATATCTAGTCTTAACTATCTCTTAATC
ATTTAAGCTTAATTTGTTCCTAGAATAGCAAATTGATTATTTATTTTTGTTCCAACCACATCTTGTTGTTGTACTTTCTTCCCTTTTTCCACCACC
TGAAATCCATCTTCATCCAGCACTGGTTCCATGGACCTCACTTCTGCTTCTTCTTTTTGAACCCAAGTTTATTTCGTTTCATTTTTATTCGTATAA
CTTTATTTTCATGATCCATATCCGAACATATCTTGCATATAATAGGGATTCATTCATACTTCGCACCCAATTCAACATCTTGATCCAATTTATCAGT
AAATGAAATTTAGTTGGAAAGTCTTGAGCAAGGCTTGCCTCAATCAAGATTCTTGGGAATAAAAGTCTGTCTCTATACTTGGTGTTGTTGTGC
ACATGAATTGGGTTCCCAATTTCTCCAAGAATTTCGAACTATGAAGGCTCTGCCCAGTACTTGATATCTAATCCCCTCACCTGGATCCACGTTA
GAACATAATCAATTTTCCCTGTAGTGAAATCGTCAAATGTATTCCATAGTTTCATAATAAAGGGTTTCTTATCAAAGAAAATGAAACTGTTGGA
TATATTTTACTAGGATCTAGATTTACTACCATGTATGTTTCATTAACAGCCTAATATGAATTCTAAAACAATGAAATAAACACATAAGAGTTTAAG
AAAACCTTACATTGGTTGCAGCGGAATATAATGACTCCTTCCGTTCAGATCTCTAGCCCTTGATTCCTTTACAGAGTGAGCATCACCAAGATCT
GAACCTGGATCTCTTTCTCTCCTTCCTTTGATGCTGAATCTCCTTCTTGTTGGTTGATTTTCCACAGTCGTACACACTATGATTGAGATACCACT
TGATGTGTGTGGGCACTCACTCATTCACTCAAGGATTTCGAAATTTAGAGAAGAAAAGAAGAGAGATGGGAATGGCTAGAAATTTTCTGGGA
AGGAATGAGATGTATCATCTTTTTCCTGAAGCCATCACTACCTATTTATAGGTAACCACCTAGGTTTAAGTTAGAATTATTTGGCATTAAAATAA
TGAAAAAATAAATGATAAAAGCCTACAATAGTGGTCGGCCTGGGCTTTGGATAATGGGCCTCACTTATGCAATTTTGCTGTTTTATCATTTCTG
CATCTCATTTTCTCAAAAATACCAATTTTCAAATTCAACCATTCAAATGCCAATTCTAATTATTTAATAACTAAAATTAATTATTAAATAATATTGT
CATTTAATATATTTATTAATTAGACATATAAAGTCTCTTAATTAATAAATAAACCTAGAATCTCTTTTCTTTACGATTTCGCCATTGCTTAGTGAAA
ATTCACAAAGTAGACATAGTCTAACTTTAGAATTATAATTGATTAATCAAAATCAATTAACTGAGTCTTACAAGCAGTATGGTCTCAACTAGTAT
GGGGACCATGGGTCTATATATCCGAGCTTCCAATAAGCAGATCAAGAATTTATATCTTAAACTCACTGACATATTAATTCTTCGTTGAATCCACG
CATAGAACTTAGAATTGCACTCTCAGTATATAGAACGCTCTATATTCCACGATATAGACACGTCATTAGTTATCCATTGTTATAATCCTAATGTGA
TCAATGACCCTCTAATAGATGATCTACATTGGAAAGGCACTAAGTTACCATTACACCTTCAATGTATTTTATCCTTAAAACACTTAGCTCCGTAT
AAATGATATTTCAGCGAAGTGAAATGAGATCTCCACCATTTATCTCTGTTTAGCCAAGCTCGAAGGATATCATCGTTTTACTTCTAAATTCTTAT
AGAAGTTATAGACTCCATATTTATGTTAGCGCTCCCACTCAATTATACTATCATGTTCCCAAAATGTACGTATCACCCTGACCCAAAAGTAGGCT
TAACTAACAAATCAAAGAACATGTATAATATTCTTGAGATCGAACCTAAACATATCAGAATTAAGATCATTTGATCTAGGATCAACAGGTGATAT
TGAATTGAATAGATATTACGGTAAATTTTAATATATCTAATCAAAGTTCAATATCGGTCCCTTCTGATGTATATTCCATACATCCGATGCTGGTAA
ACTTTGCCAATGCCCTGGAAAGGACATAACACTTATCCAAGGTGTAAGAATACCTACCGCTGATTATCATGTCAGTCTAAATCCAACGAACTG
ACAAATCAGGGAATAAACTTTCGATCATATAATTAAGATTATATTCTACTGTGTTGACAACACTATAATTATTAACAAATTGATATGTTCTGGACT
TAAATAGAATTCATACATTATGTACATATAATCATGAAATAAATCATGTGAACCATGCAACATAAAATGTTATTTCTGATCTTTATTAATAAGTAA
ATCCGATTATATTGAAATAAGTTTTATTTAAAGTATAAAACCCAACAGAAACCTCCATCAAGCACTTTGTCCCTCTGCGCCAAGTTTTTTAATTT
TATAATGAATATACCATGAGAAAGCATACCAACCTTATTCTCTTTATCTTTCCATAATCTTCTCACAAATCTGTCAAGCACACTTATGGGTGGATT
TGTAGTAGCAACATACCCAACAATCGAAGGCTATCAGTAGCTCACCTTATCTTCAATCTCTGTTGGAAGGATTTTCACTTTTGTTGTCCTCTATT
TAGGTGACTCCTCAGCTTCCTAAGGTTCATCATAACAACAATTGAGTTTCTTTACTAGATGCTCAAATCCCAGATGAGCTGATTCATCTTCTTC
CCTAACTCAACATCATGTGCTGTGCTGTTAGCTAGACTCAACCAATCTAAGAAATCCTTCTAAATCTCACTCTACTGACGAATGATCTCCTACA
ATTGTTGAGGTGAATGAATTTTCCCCAAAATCGCTTCCTCTTCTTTTCTTTTCACTTGGATCTTCAACTCTAAAATCCCTAAAATTGTATCTAAG
GATCTCATTTGTATGACTCTATCCGAAAAAGAAGGCCCTGTCTTCTTATTGTTGGCCTTCCATGTATCTTTCCCCACATTTCTTTTTCCTTTAGCT
ATTGTCATCGTTGTTTCCATCGAAAGAAACGAAGGAAGAGAGAGAAAAGATTCAATAAAGTATTTTATAAATTAACTTATGCTTTCTACACAAT
ATTTTCATGACAATTCATCACCCTTTAATACTTAGATGTCATTATACATTATATATATCTTACCACAAAAGGAATTTTAATCTAATATATGTTTCAA
ATATTTTGTTGCACTTGTAATTACTTTTTTTCATACTCTTGGTAATAAATTGCTTGAACAATATTTTTGGCACTAGAAACTATACAATATTTTCAA
AATTCATAAGTGGAACCTCATCAACACAACCACATATGTAGCCACAATATTTTGTATTCTTTCAAAACTAGATCACCTTAATTGGCTTTAGAATA
ATTAATTTATAACGAGTTGGCAAATAAACTCTATCTCTGAACAATTTTTGAATCACAAAATGGAAGCAAGAATGATGCCAACTTCGGTTCATTC
CAAGAATGGATCTATTTTCAAAGATGCATTATGCCTTTTTTTTCTTCTAGTGAACATGTTGAAGAGGATGACCTAATCATGCATATTCACAATGG
TCTTGGTTCAAAATATGTAGTCTATGATGGTCAATGTAACGCCTTTGCTTTAAGCCTCCAATGGGCAGCCACCCACAGACTTATTAGTTTAATAC
ATTGGTGTTTGTTTTTGATTACGATAATGTGAGTGCAGTAGAAGGGTGTTGGAGAGGAGAATACTACAAAATTTATATAAATCTAATTTTCATG
AATTCAGTAAGGCTCTAGAAAACCATAACAAGAACGAGATATGATAGATCTAATAGAGGATAAAATAAATAACTATTATTTGTGCATACTGCTG
ATACAATCCGAATTCACCACGAGCAAGTTCTTCTTTTCTTTGAATGCTTCCAAAATTCTATAACTACTTGTCTTCCAAACTATGGCAGTTTAGA
AAGATTAAATAGGACGAGTGGTCTCCTTTTTCAATGGTAGATTAGATCACACTTTGGAAGCTCAGATGATGAGTGTGAGATCTTTTTAGTGAA
ATAAAGAACACAAAGATTGTGTTCTTCACTATAGCAAGCAACGCACCAAGGGAAAGAGATAAAGAGAATTCTCTTAATTTCGATCAATTTTTT
TACAGTGAATAACTTAACCCTTTTTCAGTTCTGTCAAGTCTTTATTTATAAAGTTTAATAAGTGACAAGCAAGCAAACCCTCTACTTGTCTTAG
GGTATAATCTTTGGGGCTATCACACATATATAAAGATCAATACAGTAATAATGTTTTAAAAAAAATAAAAGAAAAAGAACAATATCATTATACCA
CTAGAAATAATATTAAGTGGAAACAATATTGTGATTAAAAATTTCATTAACTACTAAAAAAGTTTGTGAATAAAAGTATTAATTATAATTTTTTTT
AACTAAATGTACTTGTAAATTAGTAACAATATTATTTTATATTTAATATAATATTTCAGTAACAAGTAATATTTTATTGTGACTTAAAGTAATATTCG
TTCACTAAAAAATATTTATATTGTGAATAGACAAAATATATATAGCACGATTTTTTAAATGACATACTAAATTGTAGTTAGTAGGCTTTCCATTAA
TTTGCAATACTAAATATGTATAGTTTAACTGTCGTTATAGTAGGCTTGAATCTTTGAATCCTTTTTTCTGCACCTTAAAGTTAACAAATTTATGAA

AACTAGTACCTACTATCAATATGTATCATCATATAAAAAATTGTGCTGTAGTTTTTGTAAGTGTCAAAAATAGAGATATCCATCCTTACCTATAAA
TACAAAAATATTGTCCTTAATATAAAATTTTCGTATACATCTATCTATGTATTATGGTGTTTTAGGTTTAAGATTTGAATCACATATCTTACTCTATA
ACATTTCATTCTATTACTCTCTTAACATACTTCATCTATTATATCTAGTAGTTTCCAATATTCATAAGTCAAGTCTTAGCTTTATAAAGATTCTAATT
AAATACTCATTGTTTCAAAATTTTCGTATTGTGCACAGTTGGAGGAAATTTGTGGATTGTTTGGTAAACAAGGTGATGATCATGGGCAGCACA
ATGTTGCATATACCCACCAATCAGCTGAGCAAGTGCAACTCAATTTAGAGCCTTTGCGACACAATAATACTACTATTGCATTGCAGATCGGGTA
CGTAATAAGATTCTTTCTCTTCATCTCATAGAGATAGTATTTCTCTATACAATAATTTTGTATCTTCTGGTTAATTAAGTTGTCGTCAATATTATTTG
AATACAGTAGCTACGATGGTACTGCTAATTCAAGAATGATGAGTGGTCGTGGAGAAAATGTGGGTGCAGCCCCTTCAATGAAAAATTTGATGG
CTTTCAATTCAGTAGACCACGAATGTTTTGATCTTTAAGGCCCTAACCGATGGAGCTAGCATTACTTTTTATATACTATCCGTTTCATGTCTTCTT
AATTAATATAATGTATTGTTCGTAAAAGAGAGCACAGCTATCTTTAGCTACTCATTGGGAATTACATATATGTTAAAAGATTTAAATATTATTATAC
AATATATATACATTTATTTATGTTATCTATGTAGTGTGGAGTCATTTCATCATGAAGTAGTACTCTTACATTTTGAGGGTAAGTTTTGATGATAAGT
TTAACTTTTAAATAAGGTCTAACAATTTTGCTGAAAAAAAAAAAAATCTAAAATCTACCAGTATGTATATCAGGCCAGACCTGAAATAAAGTGG
GCCATAAGAAAAAAAAAATATTTTTGGACCCTTATGTTAGAAAAAATATGGTATTTTTCAAAGTCAGTAAAATATATAATTTTAAAAAGGGAAA
AAAAAAATTTGGACCCTTGAGCTAGGTAAGCCCTAGGCACAGGCCTAGCCCGCCTATGCCCAGGGCCGGGCCTGATGTATATGTATAAGGGAT
ATTTACACACGGTACTGTTTTTTTTATAAAACTTTCTTTTTTACTGTTACACGCATATATTTTCTTTTTTACTGTATAATCTACATATTGCATACTGA
ATTAGTATAAAGTTTTTTCCCTTTTATGTTTTTTGTCACTTCAAGATATGTTTTTTTTATTATTTTTTAAAAAAAAAAATAATAAAAGTTGATGGAA
AAATCGATTGTCACGTCAGTGACATATATATATATACATATATATTTTTTTTTTTAAAAAAAAAAAACTAATTATGAAAGTTTTATTGTATTTAATAG
TAAAAAAGTTTTGATGTGATGGTGTTGTCATTTTGTGAATTTTTTTTTTTTTTTAAAAAAAAAAAAAAAAAAACTAATCATGAAGAGCTTAATATA
CATCTTCCTAGTTTCTACACATTATCTTTTTCTTTTTAAGTATAATAGCTTTTATTACTTATTTTTCATTGTATTTGAGCTCTCTCTCTACTATTCAC
CTTCTTCTTCATTTATTTTCTTTTTTTTTTTTTTCTCAAATCTATAATATTACCCAAATAAAAAGCTATCTCTCTCCTTTTGTAGTATGTAACTAAAT
AAAAACAGCATGTGAAGGAGGAAGAAGAAGAAGAATGAGAAAGATGAGAGTGTGGTTCTGTGTGTGTGAGAGAGATGGAGAGCTTGCTCT
GTGATGAACTTCTTGTAGAAATCTTTCAGAAACTCC

>e. *LOC115701144*, PREDICTED: Cannabis sativa truncated transcription factor CAULIFLOWER A, mRNA

TATTTGCTTCTTGTCTCTCTGTTCTTTTTGGAAAATTAAATATCTATGCTAATGTTATGGTATACGGTAGGTAAGAGAGGATTAGAAAACTTTAA
CCTAAAGGCCGGATAAGAAAAAGGCATAAGGCTTATACAAGAAACTATATTTATATATATATATATAGTAGTTTAGCATATGAAGATTACTATATA
CATTTAAATTCATAATTTGGCTATATAGAAATATATATTTTTATATTTGAATATGGGTAGGGGAAAGGTGGAACTGAAGAGGATTGAAAACAAGA
TAAATCGACAAGTGACCTTTGCTAAAAGAAGGAATGGTTTGCTGAAGAAGGCTTATGAGCTCTCCGTTTTATGTGATGCTGAGGTTGCTCTAA
TAATCTTTTCTGCTCGAGGCAAACTCTTTGAGTTTTGCAGCGGCCCTAGCATCACCAAGACACTTGAGAGGTATGAAAGATGCTGCTATGCAG
AACAGGAAAGCAAACTACTATCAGGAAAGGACATAGAGTGGTCATTATCATACATACAGAGTACCTATCAAGAGTATCTAAAGCTGAAAGAA
AAAGTTGAACACCTTCGACGTACACAGAGGAATATTCTCGGGGAGGATTTGGAGCATTTGGGACTGAATGAAATTCAACAGCTTGAGCAAA
AATTGGACATGTCATTGAAGAAAATAAGATCAACTAAGAATGAGCATATGATTGATGAATTTTCTCATCTTCGAGGGCAGGAAGAAATGTTGA
TGGAAGAAAACAACAATTTAAAAAATAAGTTGGAGGAAATTTGTGGATTGTTTGGTAAACAAGGTGATGATCATGGGCAGCACAATGTTGCA
TATACCCACCAATCAGCTGAGCAAGTGCAACTCAATTTAGAGCCTTTGCGACACAATAATACTACTATTGCATTGCAGATCGGTAGCTACGATG
GTACTGCTAATTCAAGAATGATGAGTGGTCGTGGAGAAAATGTGGGTGCAGCCCCTTCAATGAAAAATTTGATGGCTTTCAATTCAGTAGACC
ACGAATGTTTTGATCTTTAAGGCCCTAACCGATGGAGCTAGCATTACTTTTTATATACTATCCGTTTCATGTCTTCTTAATTAATATAATGTATTGT
TCGTAAAAGAGAGCACAGCTATCTTTAGCTACTCATTGGGAATTACATATATGTTAAAAGATTTAAATATTATTATACAATATATATACATTTATTT
ATGTTATC

>f. LOC115701144

MGRGKVELKRIENKINRQVTFAKRRNGLLKKAYELSVLCDAEVALIIFSARGKLFEFCSGPSITKTLERYERCCYAEQESKLLSGKDIEWSLSYIQST
YQEYLKLKEKVEHLRRTQRNILGEDLEHLGLNEIQQLEQKLDMSLKKIRSTKNEHMIDEFSHLRGQEEMLMEENNNLKNKLEEICGLFGKQGDD
HGQHNVAYTHQSAEQVQLNLEPLRHNNTTIALQIGSYDGTANSRMMSGRGENVGAAPSMKNLMAFNSVDHECFDL*

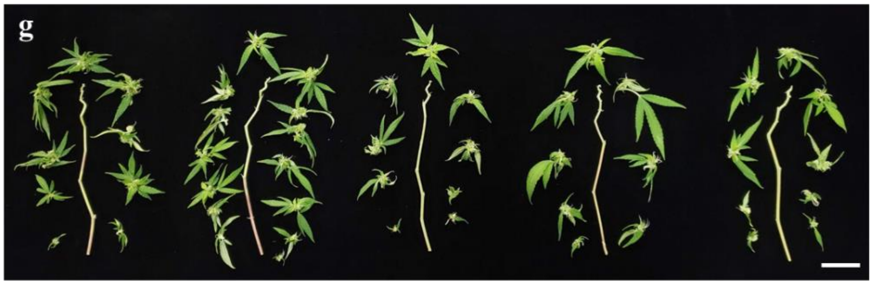

F4-063 F4-106 F4-566 F4-735 F4-928

Figure S1. Additional sequence variation in the targeted region from 33,283,738 bp to 33,422,574 bp on chromosome 8 for cloning of *QId.ibfc-8L*. a. *LOC115700576* gDNA sequence. The *LOC115700576* sequence is located between 33413255 bp and 33421815 bp in the NCBI Cannabis Sativa.cs10 reference genome. The start codon, the stop codon, and splicing sites of the intron are highlighted in yellow and with red letters. The highlighted sequences at the 5' end and 3' end served as primers to amplify *LOC115700576* in DMG12 and YMG26. The sequence differences in the promoter region are highlighted in red and square brackets. b.

LOC115700576 mRNA sequence. No difference was found in the coding region between the DMG12 and YMG26 allele. c. *LOC115700576* is annotated as a transcriptional regulator of MEF2-like/Type II subfamily of MADS box family. d. *LOC115701144* gDNA sequence. The *LOC115701144* sequence is located between 33,319,405 bp and 33,379,536 bp in the NCBI Cannabis Sativa.cs10 reference genome. The start codon, the stop codon, and splicing sites of the intron are highlighted in yellow and with red letters. The highlighted sequences at the 5' end and 3' end served as primers to amplify *LOC115701144* in DMG12 and YMG26. Two SNPs that did not result in amino acid substitution are highlighted in red and square brackets. No difference was found in the promoter region between the DMG12 and YMG26 allele. e. *LOC115701144* mRNA sequence. No difference was found in the coding region between the DMG12 and YMG26 allele. f. *LOC115701144* is annotated as a transcription factor CAULIFLOWER A F-box protein. However, the deduced protein from the coding region does not match with any F-box protein or any other known proteins. The deduced protein from the coding region of *LOC115701144* is a hypothetical protein (GenBank accession no. XP_030484710.1) in Cannabis. g. Image of dissected branches for the five critical recombinants (F4-063, F4-106, F4-566, F4-735 and F4-928). The scale bars represent 1 cm.

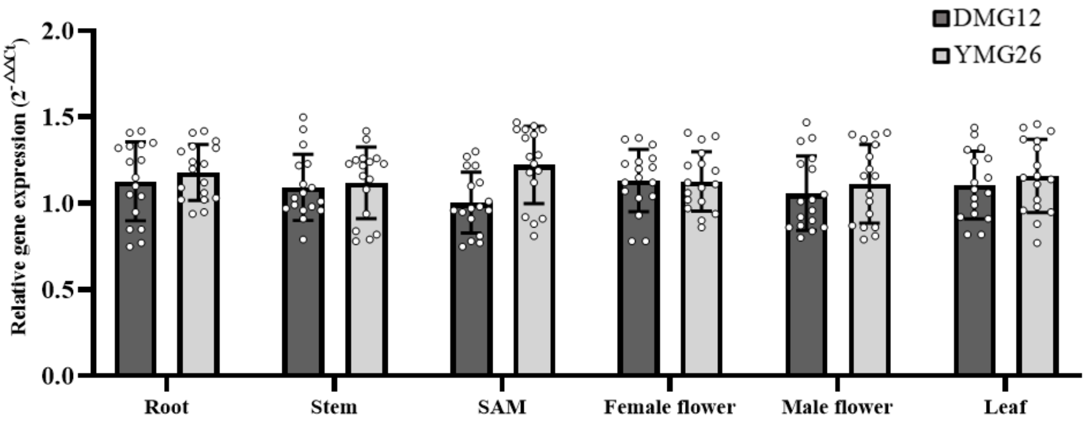

Figure S2. Transcript levels of *LOC115701144* in different tissues in DMG12 and YMG26. The RNA samples were collected one week after anthesis from different plant tissues, including root, stem, shoot apical meristem (SAM), female flower, male flower, and leaf. Transcript levels of *LOC115701144* were analyzed using qRT-PCR and calculated using the $2^{-\Delta\Delta CT}$ method, where CT is the threshold cycle. The mean transcript level between the two alleles using a two-tailed unpaired Student's t test; the bars indicate the standard error.

a.

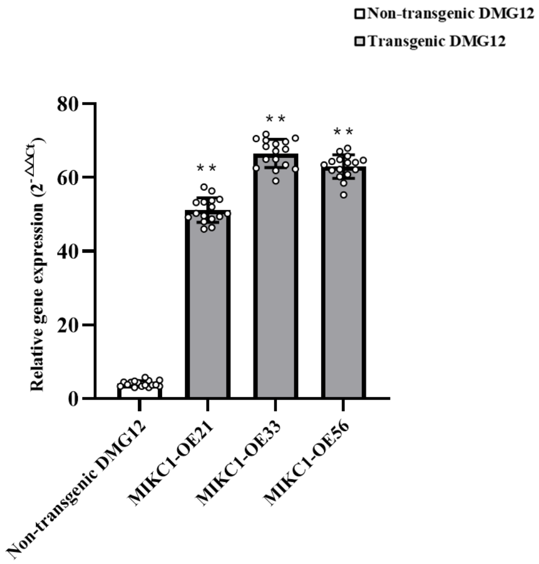

b.

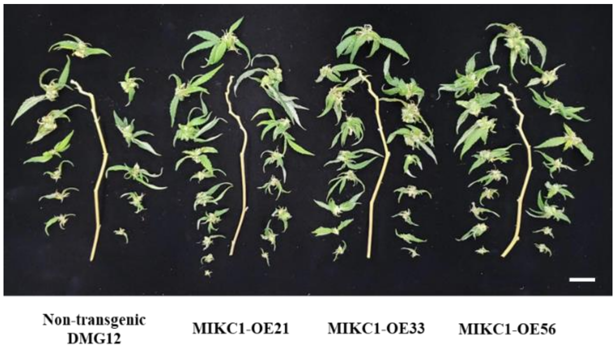

Figure S3. a. Transcript levels of *CsMIKC1* in non-transgenic DMG12 plants, MIKC1-OE21, MIKC1-OE33 and MIKC1-OE56 transgenic plants. The RNA samples were collected from seedling leaves. Specific primers MIKC1-rt-F1 and MIKC1- rt-R1 were used to amplify overexpressed *CsMIKC1* in the host plant DMG12. The transcript levels in the leaves were calculated using the $2^{-\Delta\Delta CT}$ method, where CT is the threshold cycle. The mean transcript level was analyzed (n = 16), and the ANOVA with a Tukey's HSD test shows no significance in *CsMIKC1* transcript level between different transgenic plants. b. Image of dissected branches of non-transgenic DMG12 plants, MIKC1-OE21, MIKC1-OE33 and MIKC1-OE56 transgenic plants. The scale bar represents 1 cm.

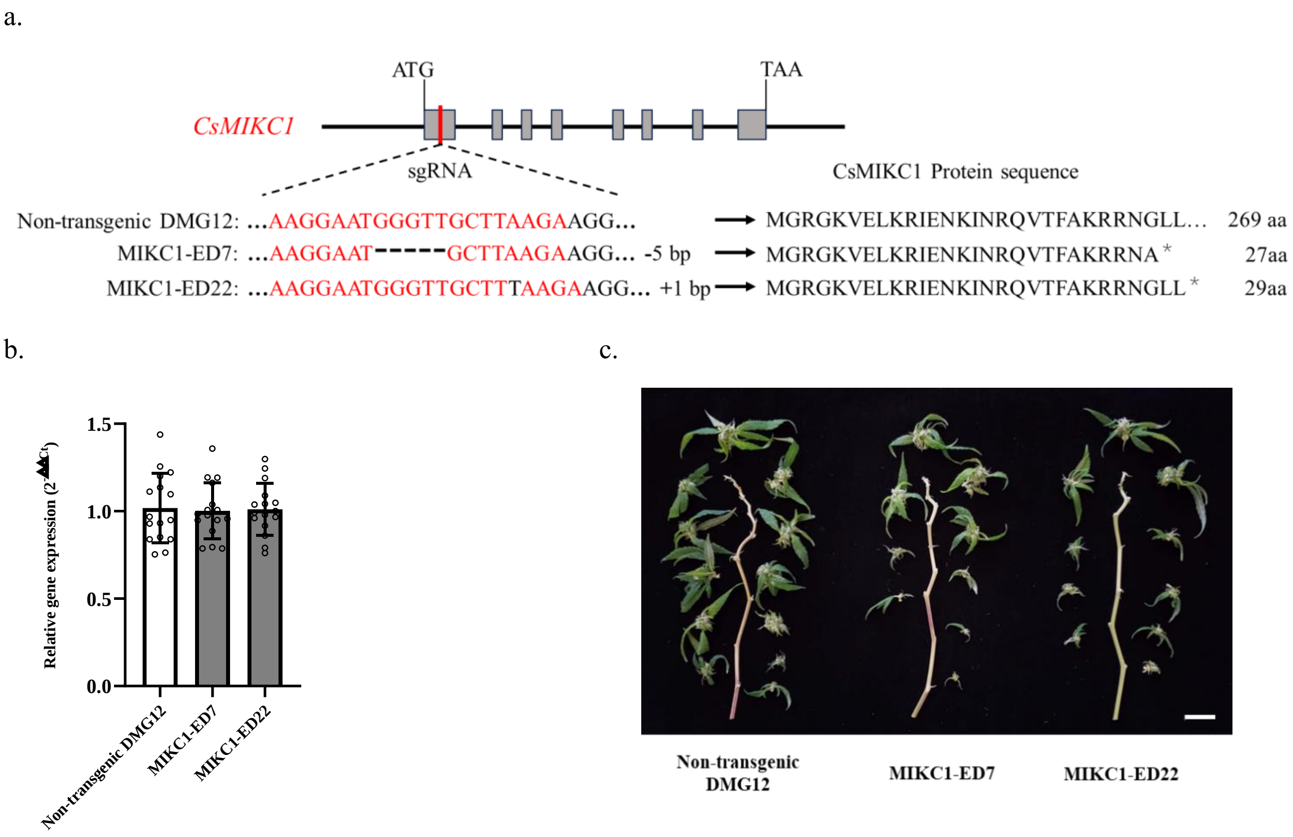

Figure S4. a. CRISPR/Cas9-mediated mutations in *CsMIKC1*. The position and sequence of the gRNA used to edit *CsMIKC1* are indicated. Edited sequences (highlighted in red) include a 5-bp deletion in the MIKC1-ED7 plant and a 1-bp insertion in the MIKC1-ED22 plant. Protein sequence alignment indicates a predicted loss of 242 a.a. starting at position 27 in the MIKC1-ED7 plant, and a loss of 240 a.a. starting at position 29 in the MIKC1-ED22 plant. The symbols '-' show the nucleotide deletion. The symbols ' * ' represents early termination of translation. b. Transcript levels of *CsMIKC1* in non-transgenic DMG12 plants, MIKC1-ED7 and MIKC1-ED22 transgenic plants. The RNA samples were collected from seedling leaves. Specific primers MIKC1-rt-F1 and MIKC1- rt-R1 were used to amplify expressed *CsMIKC1* in the host plant DMG12. The transcript levels in the leaves were calculated using the $2^{-\Delta\Delta CT}$ method, where CT is the threshold cycle. The mean transcript level was analyzed ($n = 16$), and the ANOVA with a Tukey's HSD test shows no significance in *CsMIKC1* transcript level between non-transgenic and transgenic plants. c. Image of dissected branches of non-transgenic DMG12, MIKC1-ED7 and MIKC1-ED22 mutants. The scale bar represents 1 cm.

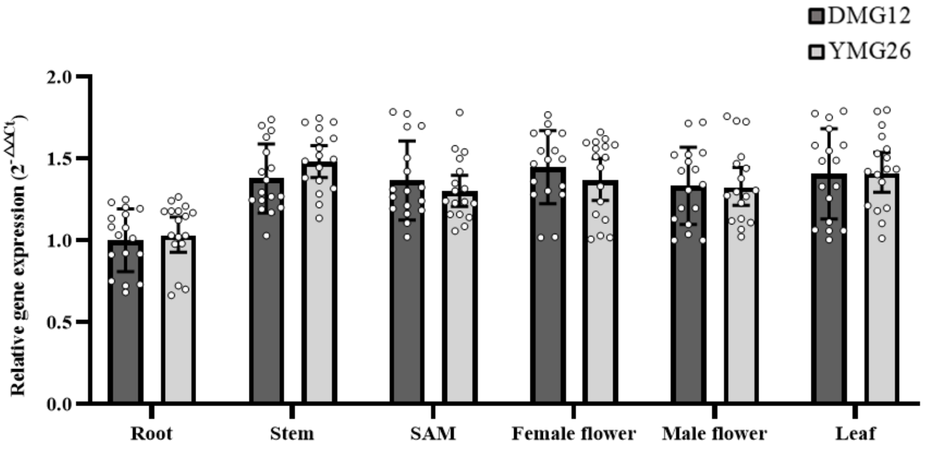

Figure S5. Transcript levels of *CsBPC2* in different tissues in DMG12 and YMG26. The RNA samples were collected one week after anthesis from different plant tissues, including root, stem, shoot apical meristem (SAM), female flower, male flower, and leaf. Transcript levels of *CsBPC2* were analyzed using qRT-PCR and calculated using the $2^{-\Delta\Delta CT}$ method, where CT is the threshold cycle. The mean transcript level between the two alleles using a two-tailed unpaired Student's t test; the bars indicate the standard error.

>a. *LOC115722185*, PREDICTED: Cannabis sativa protein BASIC PENTACYSTEINE2, genomic DNA

TTTCTTTTTCTTTCTCTCTCACAATTGTAGGGCTTTTCTCTCTCCTCTCTCCTCTCTCTTCTCTCTCTTCTCTCTCTCTCTCTCTATCTATTCTC
TCTTTCTCTCTCAGGTTTCTCTCTCCTCTCTCTCCAGAAGGGCAAAACCCTAAAGAGCCGTTTACACCCACCCAACTCTCTAATGGATGATGAT
TCGTTGCGTAACTGGGGTTACTATGAAGCGTCTTTTAAAGGTCATCTTGGCCTTCAGCTCATGTCTAGCATGTCAGACCAGCGTGATACCAAA
CCATTTCTACCTGGGCGTGATCCAACTACAGTTATGGTTAACTCCAATGGTACCTTTCATACCAATCATCCCAGGGATTGTGTTGTATCTGATGC
TCCTGTCCCAATGAACTATGGGAGGGATAGTTGGGTTAATCATAGAGACAAATTTTTGAACATGTTGCCTCCAAATACCAACTATGGTGTTCTT
CCGGAAACATCTGGTGCTCATTCGATGCAGATGTTGCAGCCACCTGAGGCTGCACCGAGGGATGAGAGGGCGGTGGTAGGTAGAATTGAAG
AGCCTGTTGTTAATAAGGAGAGTGGCCCCTCAAAGAAAAGACAGAGTGGGGGTACTCCTAAAACCCCGAAAGTGAAGAAGCCAAGGAAAC
CGAAGGAGAATAACAATGCTGCTCTTGTTCGTGTTAAGCCTGCGAAGAAGAGTATGGATGTTGTCATCAATGGGATTGATATGGACATTTCTG
GAATCCCAATTCCAGTGTGCTCATGCACTGGAACTCCACAGCAATGTTATCGATGGGGCTGCGGGGGCTGGCAATCTGCTTGTTGTACAACAA
ATGTGTCTGTTTATCCTTTGCCAATGAGTACCAAACGGCGCGGTGCTAGGATAGCTGGTAGGAAAATGAGTCAAGGTGCTTTCAAGAAGGTTT
TGGAGAAACTTGCAGCTGAAGGTTATAATTTTGCTAACCCAATTGATTTGAGGTCTTATTGGGCAAGGCATGGTACCAACAAGTTTGTCACCA
TCAGGTAGATTTACTTTGTTGGTATCTTTAGTGAGAACTCAATTGCATCTGGTATGCTTCACCTGTATATATATCTATGGCCTCTTGCAGAGATCT
TATGCTCTTCTTTTGTAGTTGTTGAATTTTCGATGTTTACTGATGATACTTGTTGTATTTGAATTGTTCTTTTGTTAAATTTTCCATTCGGCATATG
ATAAGGGGCTGATTTTTTTGGCCACTAAGACTTTATGCTGATGAGTTGTCTTTTACTGGATCGATTCTGGATAATATCGATTCTTTTTCTTTTTAA
TTTAATTCGATCACAATTAGCAATTCAATACTTTCTTGCTGTTTTGGAA

b.

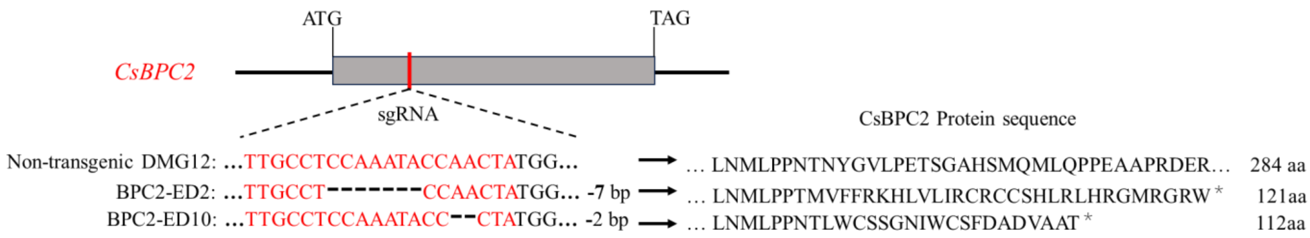

Figure S6. CRISPR/Cas9-mediated mutations in *CsBPC2*. a. *LOC115722185* gDNA sequence. The *LOC115722185* sequence is located between 56503735 bp and 56505825 bp in the NCBI Cannabis Sativa.cs10 reference genome. The start codon, the stop codon, and splicing sites of the intron are highlighted in yellow and with red letters. b. The position and sequence of the gRNA used to edit *CsBPC2* are indicated. Edited sequences (highlighted in red) include a 7-bp deletion in the BPC2-ED2 plant and a 2-bp deletion in the BPC2-ED10 plant.

Protein sequence alignment indicates a predicted loss of 163 a.a. starting at position 121 in the BPC2-ED2 plant, and a loss of 172 a.a. starting at position 112 in the BPC2-ED10 plant. The symbols '-' show the nucleotide deletion. The symbols '＊' represents early termination of translation.

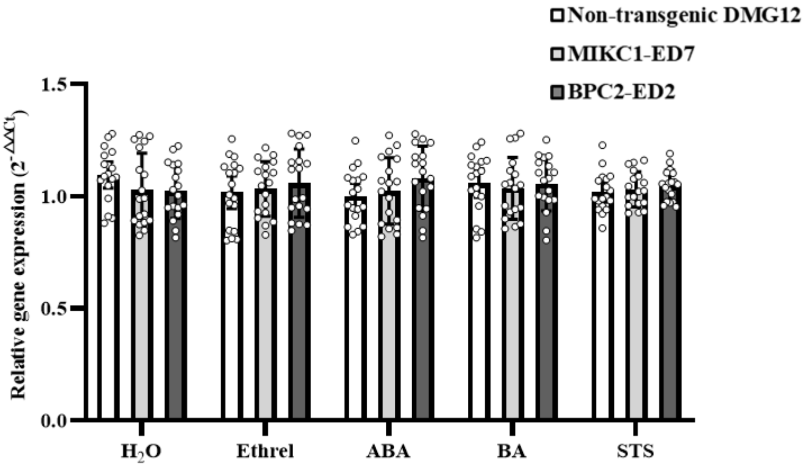

Figure S7. Relative expression levels of *CsBPC2* in non-transgenic DMG12, MIKC1-ED7 and BPC2-ED2 treated with H_2O, Ethrel (synthetic ethylene), abscisic acid (ABA), Benzyladenine (BA, a kind of synthetic cytokinin) and silver thiosulfate (STS, a ethylene inhibitor). Gene expressions were quantified by qRT-PCR with eighteen replications and calculated using the $2^{-\Delta\Delta CT}$ method, where CT was the threshold cycle. Data are presented as mean ratio ± SEM.

>*CsCOL2* (*LOC115714019*), mRNA

ATGGAAAAAATATGTGAATTTTGCTCAGAATTTAGGCCAGTGATTTACTGCAAGGCAGATGCAGCACACCTCTGCCTCTGTTGCGACGCGAA
AGTTCATTCCGCGAATGCCCTTTCGAGTAGGCATTCGAGGATGATACTTTGCGATTCTTGCAGATTTAGGCCATCTAGAGTTCAGTGGTTAGAT
CAGCAAATGTTTGTTTGTGGGATCTGTGATGGCGCCGCGCTCCACGAGGCCTCGGGAGGGCTTCAAAAGCGAGCCATCGGAAGCTTCATGG
GATCCCCTTCTGCTAAGGATTTTGCAGCCTTGTGGGGTTTTAAATTAAGTGATAATGATGATGATGATGATAAACAAGTTTTTGTGGCCTCTTCT
TCACATTCCTCTGGTGATACCAATGTGGTCAATCAACAAGTTAAGATAAAAGATGAAAAGCAGAGAAGCAGTAGTAGTTTTATTATGCAACAA
ATTATGGACTTAAAAAGGCTTCAACTCACTCAAGTGAGTAAACCTTCACCAACAACAACAACTACTAATACTAATGGCCAAGAACAAAGCAA
TGAAGAAAACGTTTCCAACAACACTTCACTGAACTCCCAAGATACCGGGACCGATTTTCGAGAGCTGAAATGTGATCCTTTCCCATGTCCATT
TTCAAAACCTGATCAATCTCCTTCATCTTCAAATGTTGGGATTCTTCCTTTGAATGGTGGAGAGCCCTTGTTGTGGCAATGTAGAAGTCCAGTT
CAGAGTAATCAGTTATGGACTCAAAATATGGAAGACCTTGGAGTATGTGAAGAACTTGTTTGGAAGGATGATTTTAATATTCCAGATGTGGATT
TGACATTTCGAAACTTTGAAGAACTATTTGATCAAGATCCAAACAGAGCTTTATTGCTTGATGATAAAGATGACTCATACTCTTCTATGGAAAA
GGGTTTGTCTCTTGATAAATTTCATAATCCTCACTCAAGAATCATGGAGGATGCTTCAGTGGATTCCTCAATTTTTGACAACAATTCTAATCTAG
CAAGCATGAACTATATGCCTTGTCCAGTTCAACCATCTTATTCAAACATGTCTCTTTCAGTTTCAAAATTTAGTGTCGATAGTGACGGTGGTGG
TAACAACCGTGGTGAATCATTGGACAGCGGCCTTTCCCCGTACTTTGGAGGAGCCGAGTCTTCTATGAATTCTCCTGATTTCGATTTCGAAGC
TAGAGAAAATGCCAAGTTGAGGTACAAAGAGAAGAAGAAATCTCGAATGCATAGACATGATAACCAAATTCGATACCCATCTAGGAAAGTAA
ATGCAGATGTGAGGAAAAGAGTAAAAGGGCGTTTTGTGAAGAGAGAAGGCTATAATTCCGATAGCGCCGACGTCGCACGAAGCTACTAA

>*CsCOL3* (*LOC115697429*), mRNA

ATGGTGAATAGTCCCAGATCTCGAACGGGTAACAGTGTGCCCTGTGATTTCTGTAGTGAACAGGTTGCTGTTCTCTTCTGTAGAGCTGACTCT
GCTAAGCTATGTTTGTTCTGCGACCAACACGTTCACTCAGCCAACCTTCTTTCACGTAAGCATCTCCGATCTCAGATCTGTGACAATTGTGGCT
CCGAACCCGTCTCCGTCCGTTGCTCTACCGAGAATCTTGTCCTTTGCCATGACTGTGATTGGGACGCTCACGGAACTTGTTCTGTCTCCCCGG
CACACGAACGGAATTCCGTCGATGGATTCTCCGGTTGCCCATCAGCTCTCGAGCTCGCCTCGGTTTGGGGTTTTCATCTTGGGGAAAAGAAAT
CGGGTCGGCCCGATTCCACAGTTCAGAATTACGAGTTGGGTATGGCTATGCCTGAGGCTGAGGACACATCTTGGTCTGGAGTGAATTTTCAG
GACCTTATTGTTCCGACCGGGAACGGCCTTGTTGGAGAAGAGGGAAAGAAACAGAGGGGAATGGGTGTTAGCTGCTGCGGGAAGAAGAAG
CAGGTGATGTATAAGCAGTTGGTGGAGTTGCTTAAGAGGGATTTGGTGGCCCCTGAAAATGACGAATTTGGCGATGATGGTTGCGGAGGAGA
CAACGGTTGTAGTGGTGGTGGTGGTGTTGAGAGCTTAATGCCGGAGAATTCAAATCGGAGTAGTTGGCAACGGAATGCTGAAGCAGCACTC
AGTTTGGGTGATGGCTGTGATGCTGTTGATGATGTTGCTTTTGCAGCTCAGCCATCTTCAACTCCATTCACGTCTCTACTAATGATGCCAATGC
ACTGTGAATTGAAAGAAAATGATGGCATTGACGATGGTGATTTGGCAATGTGGGATACCAACCCCAATGGTCCAACCACTCAGATATGGGATT
TCCATTCAGGGAGGTTGAGGGTTAATGAAGAACCAGAAATTTCATACGGGGCTAATGATGGAGGGTTTATGATTAAGAACTTTTGTGATCTTC
TGAATTGCCCTGTTGCACCAGATGACATGACTATCAATAATAGTAGCCAAGGAGCAGCAACCTCGGAGAGCAACAATCTACCTATTGGAAAG

GGGTCATCGTCTAGTACTTCAGCTTTGGGTAAGCACAAAAACGAAGAGTGTAGTGCGTCGAGAGAGGTGTATTTCATGGAACAACCTTTTCT
TGTGAGAAATGACACTATGAAAACAGGTGCAACATCAAAGGAAGAGTTGGAGGTTTTGGCTCACAACAGAGGCAATGCTATGCAACGTTAC
AAGGAAAAGAAGAAAAACAGAAGATATGACAAACACATAAGGTATGAGTCGAGAAAAGCAAGAGCTGATACTAGGAAGCGTGTAAAGGGT
CGGTTCGTGAAGGCTGCTGAAGCAGCTCCAGACGGCTAA

>*CsSOC1* (*LOC115706939*), mRNA

ATGGTGAGAGGGAAGACTCAAATGAGGCGCATAGAGAATGCCACAAGCCGTCAAGTGACGTTTTCTAAAAGGAGAAATGGGCTTCTGAAGA
AGGCCTTTGAGCTCTCAGTTCTTTGTGATGCTGAGGTGGCTCTTATAATCTTCTCCCCAAGAGGAAAACTCAATGAATTTGCTAGTTCCAGCAT
GCAAGCTACGATAGAACGTTATAGGAAGCACACTAAGGACAATCAAACTCACAACAAATCTGTTGAACAAACCATGCAGTTGCAGCATTTGA
AACAAGAAGCAACTGCCATGATGAAGAAGATAGAGCTTCTTGAAATCTCCAAACGGAAATTTTTGGGAGAAGGTCTGGGTTCATGCTCCCTT
GAAGAACTACAACAAGTAGAACAACAGTTGGAGAAGAGTGTAACAAAAATTCGAGCAAGAAAGAATCAAGTTTTCAAAGACCAGATTGAG
ATACTTAAGGAAAAGGAAAAAGCCCTAACATCTGAAAATGCAAGGCTTTCTGAGAAGTATGGTATTAAACCAATGGTTCAAGCATCAAATAAT
AATAATAATAATAATAACAATAATAATAATAATAACCATAAGCAGATAGAGGACTTGAACAACACAGAAAGTAATCCCAGTTCAGATGTGG
AGACTGAACTATTCATTGGGCTACCAGAAACAAGATTAAAGCGTCTACTTCCAAAGAAATGA

>*CsFLK1* (*LOC115716317*), RNA

ATGGCTGAACCAGGTGAACTTCCGTTAAACCCCTTGGGTACCCTCGTTGCGCCTGTTTATGGATCCGAGACCGGTCATTCAGCTGCCGGAAA
GCGCCGCCGGGAGGAATATTCTCCGGGTGTGATGTCACCGGTGGGCGATGGAGATGAGTCGGCATCCAAGCGGCGGGTCAAGGGCCAGGAC
GTGCTGTTCAGAATCGTTGTTCCGTCGAGGCAGATTGGCAAGGTCATTGGTAAAGAGGGTTGCAGAATACAGAAGATTCGGGAGGAGAGCA
AAGCCACTATCAAAATCGCCGACGCTGTAGCGCGACATGAAGAAAGAGTGGTTATTGTAAATTCAAAGGATAGTGACAACTCGGTTTCAGAT
GCTGAGAATGCTCTACAGCAAATTGCGAGTTTGATTCTGAAGGAGGATGATAGCACTCCAGAGGCATCCAAAGTTGGGGCTGGACATGTGGC
TGCGAATACAATCAGGCTTTTGATTGCTGGGTCGCAAGCAGGCTCTATAATTGGGATGTCTGGTCAGAATATCGAGAAATTAAGGAACTCATC
TGGTGCTTCTATTACCGTTCTGGCTCAGAATCAGTTGCCTTTGTGTGCTTCTGCTCATGAATCTGATCGAGTTGTACAAATATCAGGTGATGTTT
CTGCAGTTTTGAAGGCTTTAGAGGAGATAGGCTGTCAACTAAGGGAAAACCCTCCTAGACAAGTCATATCTATCAGTCCAGCTTATAATTATAC
TACAGTCCGACCGCATCCACCTTACATGGACCCATCATCAGCTGATTATGTAACTTTCGAGATGGTAATATCAGAAACCCTGGTTGGTGGTTTG
ATTGGAAGGTGCGGCTCTAACATATCAAGGATTAGGAATGAATCTGGAGCAACTATCAAGGTTTATGGTGGGAAAGGTGAACAAAATCAGAG
ACAAATTCAGTTTGGTGGGAGCACCCAACAGGTTGCACTGGCCAAGCAGAGAGTTGACGAATACATATATTCTCAGTTGATCCAACCAGCTG
GCGCTCAACAAGCAAGCAACAATACTGAGACAATGAAGGGTGGACCCAAGAACTTGGACCATGAATTCAGTTGTATACTTCATGATAAATAT
CCTTGTATTTTCAAGTCTTGCATTGCAGTTTGCTGGCCGATGCGGTTTCCGCCTCTACCTTGA

>*CsMBP21* (*LOC115709018*), mRNA

ATGGGAAGAGGGAGAGTAGAGTTGAAGAGAATAGAGAACAAAATTAACAGGCAGGTCACATTTGCCAAGAGAAGAAATGGGCTTCTCAAG
AAAGCTTATGAGCTCTCTGTTCTCTGTGAGGCTGAGGTTGCCGTTATTATTTTCTCCAACCGTGGGAAGCTTTATGAGTTCTGTAGCGGCTCCA
GTAGCATGGTGAAAACGGTAGAAAAGTACAAGAAGTATAGTTATGGAGGACTGGACGCTACTCAACCTTTAATTGACTCTCATGAGAACAGC
TATCAGGAATATTTGAAGCTAAAAGCAAGAGTAGAGGTTCTACAGAAATCTCAGAGAAACCTTCTTGGGGAAGATCTCGGACCGTTGAATAC
AAAGGAGCTCGAGCAGCTAGAGAATCAGCTGGAGATATCTTTGAGGCATATTAGGTCAACAAAGACCCAGTGCCTGCTTGATCAGCTTAATG
ATCTTCAACACAGGGAACAATTCCTACTTGAAGCTAACAAAACGTTGAGAAGGAAGTTGGAAGAAAACAGTACTCAAAATCCACTTCGACT
TGGATGGGAAGCTGCAGCGGAGGAACACAATAACAATGCCTACACCCACCTTCCTTCTCAAACAGATCAAGGCTTCTATCAGCCTCTCGGAA
ACAATTGTACCTTGCAAACTGGATACAGCAATTCGATGGGCCCAACTGAAATGAATGGTGGAGCCCCGGCCCAAACTTTGAACGGGTACATG
CATGGATGGATGCTTTGA

>*CsAGL19* (*LOC115716986*), mRNA

ATGGTTAGGGGAAAAACTCAGATGAAGCGAATAGAAAATGCAGCAAGCAGGCAAGTGACCTTTTCGAAGCGTAGAAATGGTCTTTTGAAGA
AGGCGTTTGAGTTATCAGTTCTCTGTGATGCTGAAGTTGCACTTATAATTTTCTCTCCAAGAGGAAAGCTCTATGAATTCGCAAGTTCTAGATG
CTTCAGCATAAACAAGACCATTGAGCGGTATACTCAAAGGAGAACCAAGGATGGTGGCAGCTGCCTCACCTCTAATAAAACTATTTCTGAAG
ATGATCATCATGATCTACAGGCTGCTGTAAGTAAGGAAGATTCCTTTAGCATGGCAAAGAAAATTGATCATCTTGAAGTTTCTAAAATGAAGCT
TTTGGGGGATGGATTGGAGTCATGTTCCTTCCAAGAGCTACATCAAATAGAGAATCAGTTGGAGAGAAGCTTAGCTAAAATCAGGGCAAGAA
AGAATCAACTGTACAGGGAGCAGATTGAGAAACTTAAGCAAGAGGAGAAAACTCTACTGGAGCATAATGCTAAGCTGAGGCAACAGGTACA
GTGTGGGATGTTACAAAAAGTTAGTTCAGGAAGTGAAGAGAAGGAAGATCAGGTGTTGGTGGTAGTAAATAATAATAACAATGCTCATGATC
ATCATCGTCATAACATGGACGTGGAGACGGAGTTGTTCATAGGCCTACCTGAAAGAAGAAAATAG

Figure S8. cDNA sequences of the 6 homologous genes which were commonly demonstrated to promote flowering and inflorescence development in dicot plant species. The start and stop codons are highlighted in yellow and with red letters.

>a. *LOC115707890*, genomic DNA

ATAAGTTACACAGTAAAGTGAGGCCCAATTTTAGTGAGCAGAAGCAGAAACCCAATACCCAAATAAAACCCTCAGACAACAAAACAACGCA
CAGTGAGTTACTGGCTATGAAACTCGCGGGACTCAAATCGTTCGAGAACGCCCACGATGAGTCCATCTGGGCGGCCACGTGGGTTCCGGCA
ACGGAGACCCGATCCGCCTTGCTTCTGACCGGTTCCCTCGACGAGACCGTCAAGCTATGGAAGCCAGATGACCTAATCCTCCAGCGAACCAA
TACCGGCCACTGTCTCGGAGTGGTCTCCGTCGCCGCTCACCCCTCCGGCCACTTTGCCGCATCCGCCTCCCTCGACAGCTTCGTTAGGGTTTT
CGATGTCGACACTAATAACACCTTAGCTAATCTTGAAGCTCCTCCTTCCGAAGTCTGGCAAATGCAATTTGATCCCAGGGTATACTAACACTCA
AATTACTTTTCTTTTTTTATTTATTATTTATATATTTTGTTTGACAAAAATATGTAAAGAGTAGAAGAAAATAACTTTCTTTAGGAAACTCAGTGT
TTTGTGAAAATAACGTGGACATATTTTATTAGCAAAATAACCACTTTGGTGGCACAAGAGGAGTCACTCAAAAATTATTTGGCAAATTTTATTT
GACCTTAACTGCAACATTCCGTGATTCAATGGGGTTATTTTACAAATTTTAATGCTGCCTTAAAATTAAAATTACACTATAGACCTTAAATTTAC
ACCATGTGTAAATTCAATATGTTTATTTTGTAAATTTTATGCTGTTAGTTTACCAAATTTTTCACATATATATGATGAACTAATTAGTATTGGGGTT

GTGTGAAGTGTGAACAATTAGGTTAAGATTGATTGAGGAATGGGGTTTGAAAGTTCATAATTAACCCATTCTTGATATGGTATGAATATATGTTT
TATCTATAAATGCTTTTTTCAGTTTTGTTTTCATGGATGAGTGTCTGCATTTGGTTTAAACATATTCTTTGACATTATTAGTAATAGAATAACAATA
CTGAGCTTTGGATTGTCTAGAAACCTTGTTACTCAAACAAAACAGTTATCAGTTTAAAAATTCATGGAACAATATCTATTTTACCAGGGCACCA
TCCTAGCAGTTGCAGGCGGGGGCAGCGCATCAGTGAAGCTTTGGGACACAGCCACATGGAATTTGGTTGCCACTTTATCGATTCCTCGGCCT
GAAGCAGCAAAACCATCTGAGAAAAACAGCAGCAAGAAGTTTGTTCTGTCAGTTGCTTGGAGTCCGGATGGAAGACGGCTTGCTTGTGGCT
CAATGGACGGAACTATTTCTGTGTTTGATGTTGGCCGTTCCAAGTTTCTTCACCACCTGGAAGGCCATTTCATGCCTGTACGCTCACTGGCATA
TTCTCCCACCGAACCGAGGCTGCTGTTTTCAGCCTCAGATGATGCCCATGTACACATGTACGATGCTGAGGGTAAGACTCTTGTTGGGGCCAT
GTCGGGGCACTCTAGCTGGGTGCTGAGTGTGGATGTTAGCCCAGATGGGACAGCTATTGCAACAGGTTCAAGTGACAGAACAGTGCGGCTAT
GGGACATCGGCATGAGGGCTGCTGTCCAGACAATGAGCAACCACGCCGACCAAGTTTGGGGAGTGGCCTTTGGACCAGCCGGAGGGCCTG
GTCGGCTTGCTAGTGTATCAGATGATAAGAGTATATCCCTATATGACTTCTCCTGAGAAAAGTCTCTCCTTGCAAATGAGGACTAAGAAGATTT
GTGATAGTTTAGAGTCATTGCAAATTCTTGTGGTTTTAAATTTTATCGAAAAATTGATTTCATACATTGTTAAATTATATAAAGCACTTCTAATAT
TTAATTTAAAATTGCCTAAAAAGAGGATCTTACTCCCATTGTGAAATTTGCCTGTATTCATGCTACTAGCCGAATTGTTGGCTTATTGCATTCAT
CAATATCATCAAATGTTCCATGAAA

>b. *LOC115707890*, PREDICTED: Cannabis sativa VERNALIZATION INDEPENDENCE 3, mRNA

ATGAAACTCGCGGGACTCAAATCGTTCGAGAACGCCCACGATGAGTCCATCTGGGCGGCCACGTGGGTTCCGGCAACGGAGACCCGATCCG
CCTTGCTTCTGACCGGTTCCCTCGACGAGACCGTCAAGCTATGGAAGCCAGATGACCTAATCCTCCAGCGAACCAATACCGGCCACTGTCTC
GGAGTGGTCTCCGTCGCCGCTCACCCCTCCGGCCACTTTGCCGCATCCGCCTCCCTCGACAGCTTCGTTAGGGTTTTCGATGTCGACACTAAT
AACACCTTAGCTAATCTTGAAGCTCCTCCTTCCGAAGTCTGGCAAATGCAATTTGATCCCAGGGGCACCATCCTAGCAGTTGCAGGCGGGGG
CAGCGCATCAGTGAAGCTTTGGGACACAGCCACATGGAATTTGGTTGCCACTTTATCGATTCCTCGGCCTGAAGCAGCAAAACCATCTGAGA
AAAACAGCAGCAAGAAGTTTGTTCTGTCAGTTGCTTGGAGTCCGGATGGAAGACGGCTTGCTTGTGGCTCAATGGACGGAACTATTTCTGTG
TTTGATGTTGGCCGTTCCAAGTTTCTTCACCACCTGGAAGGCCATTTCATGCCTGTACGCTCACTGGCATATTCTCCCACCGAACCGAGGCTG
CTGTTTTCAGCCTCAGATGATGCCCATGTACACATGTACGATGCTGAGGGTAAGACTCTTGTTGGGGCCATGTCGGGGCACTCTAGCTGGGTG
CTGAGTGTGGATGTTAGCCCAGATGGGACAGCTATTGCAACAGGTTCAAGTGACAGAACAGTGCGGCTATGGGACATCGGCATGAGGGCTG
CTGTCCAGACAATGAGCAACCACGCCGACCAAGTTTGGGGAGTGGCCTTTGGACCAGCCGGAGGGCCTGGTCGGCTTGCTAGTGTATCAGA
TGATAAGAGTATATCCCTATATGACTTCTCCTGA

>c. LOC115707890

MKAGKSNAHDSWAATWVATTRSATGSDTVKWKDDRTNTGHCGVVSVAAHSGHAASASDSVRVDVDTNNTANASVWMDRGTAVAGGGSASVK
WDTATWNVATSRAAKSKNSSKKVSVAWSDGRRACGSMDGTSVDVGRSKHHGHMVRSAYSTRSASDDAHVHMYDAGKTVGAMSGHSSWVSV
DVSDGTAATGSSDRTVRWDGMRAAVTMSNHADVWGVAGAGGGRASVSDDKSSYDS

d.

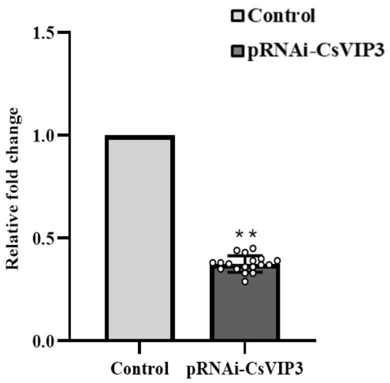

Figure S9. Effect of pRNAi-CsVIP3 vector on *CsVIP3* relative expression change. a. *LOC115707890* gDNA sequence. The *LOC115707890* sequence is located between 50441750 bp and 50443740 bp in the NCBI Cannabis Sativa.cs10 reference genome. The start codon, the stop codon, and splicing sites of the intron are highlighted in yellow and with red letters. b. *LOC115707890* mRNA sequence. c. *LOC115707890* is annotated as a WD repeat-containing protein VERNALIZATION INDEPENDENCE 3 (VIP3). d. Relative fold change was investigated three days post agroinfiltration. Leaf material infiltrated with disarmed *Agrobacterium* were set as control. Significance is determined by paired t-test, ($p<0.01$) is denoted by **. Error bars represented SE.
